# Reduction of Metastasis via Epigenetic Modulation in a Murine Model of Metastatic Triple Negative Breast Cancer (TNBC)

**DOI:** 10.3390/cancers14071753

**Published:** 2022-03-30

**Authors:** Jessica L. S. Zylla, Mariah M. Hoffman, Simona Plesselova, Somshuvra Bhattacharya, Kristin Calar, Yohannes Afeworki, Pilar de la Puente, Etienne Z. Gnimpieba, W. Keith Miskimins, Shanta M. Messerli

**Affiliations:** 1 Department of Biomedical Engineering, University of South Dakota, Sioux Falls, SD 57107, USA; jessica.simcox@coyotes.usd.edu (J.L.S.Z.); mariah.hoffman@coyotes.usd.edu (M.M.H.); etienne.gnimpieba@usd.edu (E.Z.G.); 2 2-Dimensional Materials for Biofilm Engineering Science and Technology (2DBEST) Center, Sioux Falls, SD 57107, USA; 3 Cancer Biology & Immunotherapies, Sanford Research, Sioux Falls, SD 57104, USA; simona.plesselova@sanfordhealth.org (S.P.); somshuvra.bhattacharya@sanfordhealth.org (S.B.); kristin.calar@sanfordhealth.org (K.C.); pilar.puente@sanfordhealth.org (P.d.l.P.); keith.miskimins@sanfordhealth.org (W.K.M.); 4 Functional Genomics and Bioinformatics Core, Sanford Research, Sioux Falls, SD 57104, USA; yohannes.tecleab@sanfordhealth.org; 5 Department of Surgery, University of South Dakota Sanford School of Medicine, Sioux Falls, SD 57105, USA; 6 Department of Chemistry and Biochemistry, South Dakota State University, Brookings, SD 57006, USA; 7 Department of Biology and Microbiology, South Dakota State University, Brookings, SD 57006, USA

**Keywords:** TNBC, 4SC-202, metastasis, cancer stem cells

## Abstract

**Simple Summary:**

We investigate the use of the small molecule epigenetic modulator 4SC-202 as a potential cancer therapeutic or adjuvant for Triple Negative Breast Cancer (TNBC). Epigenetic modulation involves alteration of the cellular phenotype without altering the genotype. Here, we investigate how 4SC-202 affects tumor growth, the ability of tumor cells to migrate, and the growth of tumors in vivo. This study demonstrates that 4SC-202 kills tumor cells, reduces their ability to migrate, and decreases metastasis of the tumor cells and tumor burden in a highly metastatic murine model of TNBC. These preclinical studies provide critical information on the potential efficacy of 4SC-202 as a potential therapeutic or adjuvant for TNBC.

**Abstract:**

This study investigates the effects of a dual selective Class I histone deacetylase (HDAC)/lysine-specific histone demethylase 1A (LSD1) inhibitor known as 4SC-202 (Domatinostat) on tumor growth and metastasis in a highly metastatic murine model of Triple Negative Breast Cancer (TNBC). 4SC-202 is cytotoxic and cytostatic to the TNBC murine cell line 4T1 and the human TNBC cell line MDA-MB-231; the drug does not kill the normal breast epithelial cell line MCF10A. Furthermore, 4SC-202 reduces cancer cell migration. In vivo studies conducted in the syngeneic 4T1 model, which closely mimics human TNBC in terms of sites of metastasis, reveal reduced tumor burden and lung metastasis. The mechanism of action of 4SC-202 may involve effects on cancer stem cells (CSC) which can self-renew and form metastatic lesions. Approximately 5% of the total 4T1 cell population grown in three-dimensional scaffolds had a distinct CD44^high^/CD24^low^ CSC profile which decreased after treatment. Bulk transcriptome (RNA) sequencing analyses of 4T1 tumors reveal changes in metastasis-related pathways in 4SC-202-treated tumors, including changes to expression levels of genes implicated in cell migration and cell motility. In summary, 4SC-202 treatment of tumors from a highly metastatic murine model of TNBC reduces metastasis and warrants further preclinical studies.

## 1. Introduction

Despite an overall decrease in breast cancer mortality in the past twenty years, successful treatment remains a formidable challenge, largely due to breast cancer’s molecular, histological and clinical heterogeneity. Triple Negative Breast Cancer (TNBC) in particular has been difficult to treat. TNBC, so named because of its lack of receptors for estrogen, progesterone, and over-expressed HER2, which are important targets for anti-cancer drugs [1], has higher rates of metastasis and death than other types of breast cancer [2]. In this study, we explored the possible therapeutic benefit of a small molecule benzamide dual histone deacetylase (HDAC) Class I and lysine-specific histone demethylase 1A (LSD1) inhibitor, 4SC-202 (Domatinostat), on tumor burden and metastasis in a highly metastatic TNBC murine model (4T1). 

We have previously demonstrated that 4SC-202 is both cytostatic and cytotoxic in the pediatric cancer medulloblastoma [3] and a rare childhood brain cancer known as Atypical Teratoid Rhabdoid Tumor (ATRT) [4]. 4SC-202, also known as Dominostat, is a targeted HDAC inhibitor which inhibits HDAC Class I including HDAC’s 1, 2, 3, 8 and KDM1A/LSD1 (Lysine (K)-specific demethylase 1A). Additional studies by other investigators have also showed cytotoxicity and reduced cell proliferation in vitro in a number of different cancers including in colorectal cancer [5], urothelial cancer [6], cutaneous T cell lymphoma [7], and a highly metastatic pancreatic cell line [8]. In terms of the potential mechanism of action, our prior studies have suggested that 4SC-202 has an effect on cancer stem-like cells [4]. It has been shown that aldehyde dehydrogenase (ALDH) is a marker of cancer stem cells (CSCs). ALDH high cells have a role in metastasis and in the 4T1 syngeneic mouse model of breast cancer, inhibition of ALDH activity suppresses stem-like cell properties [9].

Based on our previous finding in ATRT cell lines, as well as other findings in a number of different cancers, we hypothesize that 4SC-202 will significantly reduce growth of tumors and rate of metastasis and may have an effect on CSCs. The questions addressed in this study include examination of the effect of the dual HDAC/LSD inhibitor 4SC-202 in TNBC on migration, tumor growth and metastasis, as well as addressing the cellular and molecular mechanism of action. 

We also demonstrate that dual inhibition of HDAC1 and LSD1 with the orally bioavailable epigenetic modulator 4SC-202 in the highly metastatic murine model of TNBC 4T1 reduces tumor burden. 4SC-202 treatment was compared to the vehicle or the broad spectrum HDAC inhibitor Vorinostat, which is used clinically to treat cutaneous T-cell lymphoma. 4SC-202 significantly reduced lung metastasis as compared to the vehicle or Vorinostat treatment and induced significant changes in metastasis-related processes such as cell migration and motility, suggesting that 4SC-202 has the potential to serve as an adjuvant to traditional chemotherapy. However, more pharmacogenetic preclinical studies need to be performed to examine the efficacy and safety of 4SC-202 in combination with standard of care chemotherapy.

## 2. Materials and Methods

### 2.1. Cell Culture and In Vitro Assays

The highly metastatic murine Triple Negative Breast Cancer (TNBC) breast cancer cell line 4T1, human epithelial TNBC MDA-MB-231, and the human non-tumorigenic human breast epithelial MCF10 cells were all obtained from ATCC. These lines were used within the first five passages and grown in DMEM containing 10% Fetal Bovine Serum (FBS), penicillin/streptomycin solution, and Amphotericin in 5% CO_2_, as described [10]. For viability experiments, equal numbers of 4T1, MDA-MB-231 and the normal human mammary epithelial cell line MCF10A were plated on white flat bottomed 96 well plates, and 24 h later, treated with 4SC-202 at concentrations ranging from 0.1 nM to 10 µM (Selleckchem). After 72 h of drug treatment, the viability of cells was assessed by staining with Sytox Green (Thermofisher, Waltham, MA, USA) as previously described [11]. Fluorescence was measured using a Cytation 3 Imaging reader (Biotek, Winooski, VT, USA). Colony assays using concentrations of 4SC-202 ranging from 0.1 nM to100 µM were performed as previously described [11]. For Aldefluor experiments, 4T1 cells were treated with 4SC-202 at concentrations ranging from 1 to 100 µM and then ALDH activity was accessed using the ALDEFLUOR kit (STEMCELL Technologies, Cambridge, MA, USA) by flow cytometry using the BD Accuri™ C6 Plus Flow Cytometer. 

### 2.2. Scratch Assay

Cell migration was measured and analyzed using the scratch assay, as described in Liang et al., 2007 [12]. Relative migration of cells was assessed by measurement of the area of scratch which remained cell free. Briefly, 4T1 cells were plated on 60 mm plastic tissue culture dishes, and a scratch was made with a p200 pipet tip in both the control and drug conditions. Concentrations of 4SC-202 were applied at concentrations ranging from 0.1 to 100 µM 4SC-202. Cells were imaged using EVOS XL Cell Imaging system (Life Technologies, Carlsbad, CA, USA) at 0 and 6 h, following application of drugs. Images were analyzed with EVOS software, and the area of scratch was quantified.

### 2.3. Spheroid Models

4T1, MDA-MB-231, and MCF10A cells were plated on ultralow attachment 96 well spheroid plastic black microplate plates with clear bottoms (Corning, Corning, NY, USA) and treated with 4SC-202/vehicle (DMSO) 24 h after plating. Seven days after plating, luminescent cell viability assays (Cell Titer Glow 3D cell viability assay, Promega, Madison, WI, USA) were performed using a 96 Microplate GlowMax luminometer (Promega).

### 2.4. Three-Dimensional Scaffold Model

4T1 cells and MDA-MB-231 cells (0.4 × 106 cells/mL) were embedded in a plasma three-dimensional (3D) scaffold engineered through the cross-linking of fibrinogen into fibrin as previously described [13,14]. Briefly, plasma was mixed with 4T1 or MDA-MB-231 cell suspensions prepared in RPMI complete media followed by encapsulation into matrices that were fabricated using calcium chloride (CaCl_2_) as a cross-linker and trans-4-(aminomethyl) cyclohexanecarboxylic acid (AMCHA) as a stabilizer, respectively. At the end of day 0, 4T1 or MDA-MB-231 cells seeded within the 3D matrix were treated with 4SC-202 at a range of concentrations (1–100 μM) for 3 days. Three-dimensional matrices containing 4T1 or MDA-MB-231 cells treated with DMSO (at the same concentrations as in the 4SC-202 treatments) were used as controls. 

### 2.5. Flow Cytometry Studies for 3D Cultures

After 3 days of drug treatment, 3D matrices containing 4T1 cells were enzymatically digested with collagenase (20 mg/mL for 2–3 h at 37 °C). Cell viability (survivability) was evaluated by using a Live/Dead Blue cell stain (L34962, Thermo Fischer Scientific, MA, USA). Pre-labeled 4T1 cells with DiO (10 µg/mL, Invitrogen, Waltham, MA, USA) were identified by gating live cells with a DiO+ signal using an FITC channel. Counting beads (424902, Biolegend, San Diego, CA, USA) were added, a minimum of 10,000 events were acquired to collect and interpret data using BD FACS Fortessa and FACSDiva v6.1.2 software (BD Biosciences, San Jose, CA, USA). The 4T1 cell counts were always normalized to a predetermined number of counting beads, and the data were analyzed using FlowJo^TM^ program v10 (BD Life Sciences, Ashland, OR, USA). In order to determine the effect of 4SC-202 on cancer stem cell (CSC) population, the cells retrieved from the matrices were probed with anti-CD44 antibody conjugated to BV605 fluorophore (400650, Biolegend, San Diego, CA, USA) and anti-CD24 antibody conjugated to PE fluorophore (FAB8547P, R&D Systems, Minneapolis, MN, USA). Fluorescence minus one (FMO) controls were used to determine the appropriate gating strategy. 4T1 CSC cells were identified by gating cells demonstrating CD44^high^ and CD24^low^ signal or by evaluating the expression of CD24 population gated inside the CD44^high^ population [15]. Percentage of 4T1 CSC cells after 4SC-202 treatment was determined and histograms showing the changes in CD24 expression of the CD44^high^ population were also analyzed.

### 2.6. Scaffold Image Analysis

Three-dimensional matrices after drug treatment for 3 days were fixed in 10% neutral buffered formalin and processed on a Leica 300 ASP tissue processor. The 3D matrices were oriented on the slide so that the surface of the scaffold section always faced forward, as previously described [16]. Paraffin-embedded scaffold sections were longitudinally sliced at 10 microns. The BenchMark^®^ XT automated slide staining system (Ventana Medical Systems, Inc., Oro Valley, AZ, USA) and the Ventana iView DAB detection kit was used as the chromogen, and the slides were counterstained with hematoxylin and eosin. H&E images of sectioned matrix slices were imaged using an Aperio VERSA Bright field Fluorescence and FISH Digital Pathology Scanner (Leica Biosystems, Deer Park, IL, USA). 

### 2.7. Mouse Studies

A total of 40 6-week-old female BALB/c mice (Jackson Laboratory) were maintained as previously described [11]. 4T1 cells (2.5 × 10^4^) were injected subcutaneously into the hind limb of female BALB/c mice. In the first in vivo experiment examining how 4SC-202 affected tumor growth and metastasis (Experiment 1), the experimental groups received 4SC-202 delivered at two different concentrations (50 mg/kg; 75 mg/kg), and the control group received 0.001% DMSO vehicle saline. Doses were determined at consultation with colleagues at 4SC AG based on company data. Thirty mice were randomly divided into the following groups: 4SC-202 (50 mg/kg), Vorinostat (50 mg/kg), and control vehicle saline (0.001% DMSO). When tumors became palpable, mice were randomly divided into groups, with group size determined based on power analysis and prior animal studies [17]. Intraperitoneal (i.p.) injections with 4SC-202, Vorinostat, and vehicle saline were performed three times a week. After 21 days, mice were euthanized with 5% CO_2_. At the time of sacrifice, lungs were injected with India Ink. All organs were flash frozen with liquid nitrogen or fixed in 10% formalin.

In the second in vivo 4T1 experiment (Experiment 2), 2.5 × 10^4^ 4T1 cells were subcutaneously injected into 30 BALB/c mice, the mice were then divided into the following groups (10 mice each): (1) control vehicle, (2) 4SC-202 (50 mg/kg), and (3) Vorinostat (50 mg/kg) delivered intraperitoneally (i.p.). When tumors became palpable (4 days), the groups received the following treatments in weeks 1–3: Control group: Vehicle (0.9% Sodium Chloride saline containing 0.001% DMSO), 4SC-202 (50 mg/kg), and Vorinostat (50 mg/kg). Tumors were measured twice per week with calipers. The 4SC-202 and Vehicle injections were delivered 3 times per week. All mice were housed in the animal facility of the Sanford Center at Sanford Research, and experiments were performed according to the approved Institutional Animal Care and Use Committee (IACUC) protocol #157-03-223. 

### 2.8. RNA-Sequencing

RNA from two independent animal experiments was sequenced (Experiment 1, Experiment 2). In the first experiment, two tumors from each group were flash-frozen with liquid nitrogen. Then, RNA was extracted using the Qiagen RNeasy mini kit according to the manufacturer’s protocol. RNA quality was analyzed using the Agilent 2100 Bioanalyzer (Supplementary File S6) and the sequencing was performed by the Sequencing Core at South Dakota State University (Brookings, SD, USA) using the NextSeq 500 (paired-end, 75 base pairs). For the second experiment, RNA was sequenced from tumors from the following groups: three vehicle control, three 4SC-202 (50 mg/kg) treated, and four Vorinostat (50 mg/kg) treated. RNA was extracted from the tumors, which were flash frozen with liquid nitrogen following removal from the animal. RNA concentrations were assessed using a NanoDrop spectrophotometer (ThermoFisher). RNA quality was determined using an Agilent 2100 Bioanalyzer and Agilent RNA 6000 Pico kit (Santa Clara, CA, USA) and sequencing was performed at the Sanford Burnham Prebys Sequencing Core using the NextSeq 500 (paired-end, 75 base pairs). 

### 2.9. Genomic Data Commons Analysis

Basal-like breast cancer samples were identified in the Genomic Data Commons (GDC) database based on the PAM50 classification from a published table of GDC metadata [18] and normal breast tissue samples were selected based on cell classification data. HTSeq-Count files and metadata files were downloaded from the GDC on June 5, 2019. Sample type was verified as “tumor” or “normal” (tissue from metastases was not included) leaving *n* = 18 normal breast tissue samples and *n* = 165 basal-like breast tumor samples. Differential expression was run using DESeq2 (1.24.0) [19], and log fold shrinkage was applied using apeglm (1.6.0) [20]. For visualization of gene expression values, data were transformed using the variance stabilizing transformation implemented in DESeq2 and then z-scores were calculated for each row. *P*-values were adjusted by the Benjamini–Hochberg (BH) method.

### 2.10. Bioinformatic Analysis

Raw sequencing reads from both Experiment 1 and Experiment 2 were checked for read quality using FASTQC and analyzed using three different workflows to account for method specific variation in mapping to GRChm38 and calling of differentially expressed genes (DEGs) (Supplementary Figure S1). A final consensus set of DEGs was identified as those genes that are found to be DE in all three approaches at an adjusted *p*-value of 0.1, to serve as self-validation of the DEGs. Data from Experiment 2 were used to compare differences between Vorinostat and 4SC-202. For Workflow 1 (also referred to as WF1), we used STAR for mapping and DESeq2 for differential expression (DE) analysis with biological experiments included as covariates, when appropriate. For Workflow 2 (also referred to as WF2), Kallisto was used for pseudoalignment followed by differential expression with limma (meta-analysis) or DESeq2 (individual analyses) using NetworkAnalyst (2020). In the 4SC-202 versus control comparison, Fisher’s method was used to combine datasets. For Workflow 3 (also referred to as WF3), HISAT2 was used for alignment and DESeq2 for differential expression [19]. Where appropriate, surrogate vectors were included as covariates to account for batch effects. Enrichment analyses were conducted using the goseq R package, clusterProfiler R package, and Ingenuity Pathway Analysis; functional annotation clustering was performed using the Database for Annotation, Visualization and Integrated Discovery (DAVID). Further details are available in the Supplementary Bioinformatic Methods (Appendix A).

### 2.11. Statistics

Unless otherwise noted, data are presented as the mean ± SEM. In one-factor experiments with two levels, equal variance is tested for using a two-sample F-test (*p* < 0.05 significance threshold) and an appropriate paired-end *t*-test is then used for hypothesis testing. Otherwise, Analysis of Variance (ANOVA) is utilized followed by a post-hoc Dunnett’s or Tukey’s test, as appropriate. In these tests, *p* < 0.05 (*), *p* < 0.01 (**), and *p* < 0.001 (***) was considered statistically significant. Statistical testing and thresholding in bioinformatic analyses are detailed above.

## 3. Results

### 3.1. 4SC-202 Targets Are Overexpressed in Human Basal-like Tumors

In order to establish whether or not 4SC-202 would be a good candidate drug for the treatment of TNBC, we evaluated the transcriptomic profile of 4SC-202 targets in basal-like breast tumor tissue. While TNBC and basal-like breast cancer classifications are not synonymous, there is a strong overlap between the classifications [21,22]. The Prediction Analysis of Microarray 50 (PAM50) basal-like classification has the advantage of being gene expression-based. Candidate basal-like breast tumors from the Genomic Data Commons (GDC) were selected on the basis of a published PAM50 classification [18] and normal samples were selected based on GDC metadata, resulting in *n* = 18 normal breast tissue samples and *n* = 165 primary basal-like breast tumors samples.

**Figure 1:**
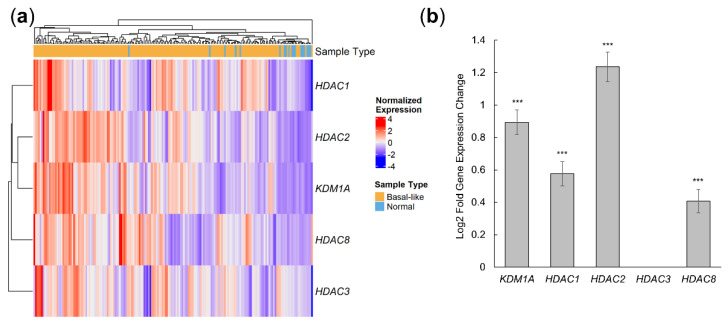
4SC-202 targets are upregulated in human basal-like breast cancer tumors relative to normal tissue. Genomic Data Commons RNA-seq data for normal breast tissue (*n* = 18) and basal-like breast tumor tissue (*n* = 165) were analyzed for differential expression using DESeq2. (**a**) Heatmap of row z-scores for variance-stabilized gene expression values across normal and basal-like samples. Sample type is annotated at the top. (**b**) Log2 fold expression changes of basal-like tumors versus normal breast tissue. Error bars indicate the standard error of the mean of the log fold change; FDR < 0.001 (***).

Gene expression levels were evaluated across tumors and between normal and basal-like breast tumor samples using GDC RNA-sequencing results. Since 4SC-202 is a dual epigenetic modulator for Class I HDAC and LSD1, we investigated the expression pattern for Class I HDACS, including *HDAC 1, 2, 3*, and *8*, and the lysine demethylase *KDM1A* (LSD1). If these targets are overexpressed in basal-like breast tumor samples, the likelihood of 4SC-202 being an effective therapeutic is improved. These epigenetic targets of 4SC-202 are sufficient to cluster basal-like and normal samples, with basal-like samples exhibiting higher overall expression (Figure 1a). Quantification of these differences show that *KDM1A*, *HDAC1*, *HDAC2*, and *HDAC8* have significantly higher expression levels (Figure 1b). These results indicate that targets of 4SC-202 are overexpressed in human breast cancer with a basal-like molecular signature.

### 3.2. 4SC-202 Treatment Is Cytotoxic and Cytostatic to TNBC

**Figure 2:**
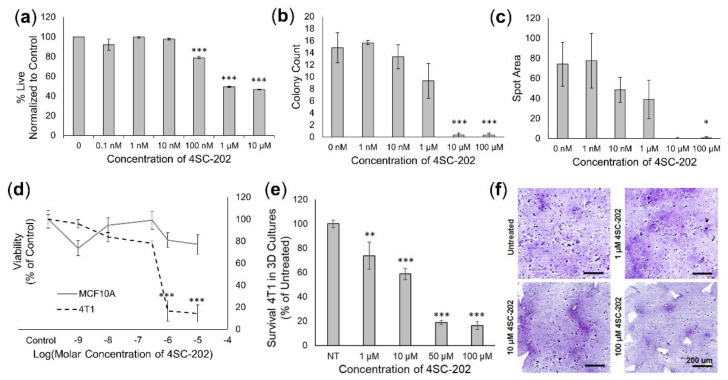
4SC-202 is cytotoxic and cytostatic in metastatic TNBC murine cell line 4T1. (**a**) Viability of 4T1 cells measured by a Sytox Green live fluorescence assay indicates 4SC-202 is significantly cytotoxic at concentrations ranging from 100 nM to10 µM, measured 72 h after treatment for *n* = 8 biological replicates. (**b**) 4SC-202 is cytotoxic to colony number formation after 6 days exposure at concentrations ranging from 10 to 100 µM, and (**c**) significantly reduces the area of colonies at 100 µM indicating a cytostatic effect (*n* = 3 biological replicates except 0, 100 µM where *n* = 6 biological replicates). (**d**) While significantly cytotoxic to 4T1 spheroids at 1-10 µM, 4SC-202 is not cytotoxic to normal breast epithelial MCF10A spheroids for *n* = 6 replicates. (**e**) Survivability of 4T1 in a three-dimensional scaffold model as measured by flow cytometry indicates 4SC-202 is significantly cytotoxic to 4T1 at concentrations ranging from 1 to 100 µM for *n* = 3 scaffold experiments with 3 technical replicates. (**f**) H and E-stained images of sectioned 3D scaffold models indicates a reduced number of nuclei following exposure of 4T1 to 4SC-202 at concentrations ranging from 1 µM to 100 µM. Significance relative to control group was determined using ANOVA with a post hoc Dunnett’s test where adj *p* < 0.05 (*); adj *p* < 0.01(**); adj *p* < 0.001(***). Error bars indicate the standard error of the mean (SEM).

In cell culture, 4SC-202 has both cytotoxic and cytostatic effects on 4T1 cells. Cell viability experiments using the Sytox Green Fluorescence assay indicate 4SC-202 is significantly cytotoxic at concentrations ranging from 0.1 µM to 10 µM (Figure 2a) following 72 h of exposure. Colony assays similarly indicate that 4SC-202 significantly reduces the number of colonies formed after 6 days, indicating cytotoxicity at concentrations ranging from 10 to 100 µM (Figure 2b), and significantly reduces the area of colony formation (mean spot area) at 100 µM (Figure 2c), indicating that the drug may exert a cytostatic effect. In 3D spheroid models, viability assays indicate that while 4SC-202 is cytotoxic to breast 4T1 cancer cells at concentrations ranging from 1 to 10 µM, it is not cytotoxic to the non-cancer breast epithelial cell line MCF10A (Figure 2d). Similarly, 4T1 cells grown within a 3D scaffold exhibited significantly reduced dose-dependent survivability when treated with increasing concentrations of 4SC-202 after 72 h of exposure (Figure 2e), with a reduced number of hematoxylin stained 4T1 cell nuclei observed with increasing concentrations of 4SC-202 (Figure 2f). 

**Figure 3:**
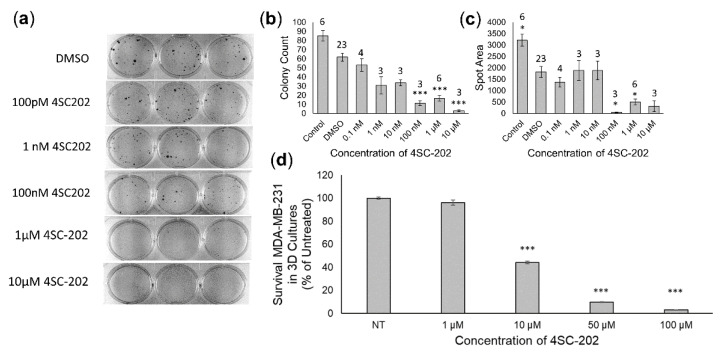
4SC-202 is cytotoxic to human TNBC MDA-MB-231. (**a**,**b**) Concentrations of 4SC-202 ranging from 100 nM to 10 µM significantly reduced the number of colonies and (**c**) concentrations ranging from 0.1 to1 µM significantly reduced the area of colonies indicating both a cytotoxic and cytostatic effect. Number of replicates is indicated above each bar. (**d**) Using a 3D-scaffold for MD-MB-231, the survival of cells decreased significantly (10 µM, 50 µM, 100 µM) as the concentration of 4SC-202 was increased for *n* = 3 scaffold experiments with 3 technical replicates. Hypothesis testing was conducted using ANOVA followed by a post hoc Dunnett’s test. Significance differences are indicated relative to DMSO vehicle (**b**,**c**) or untreated (NT) (**d**) groups (adj *p* < 0.05 *, adj *p* < 0.001 ***). Error bars are the standard error of the mean (SEM).

Moreover, cytotoxic and cytostatic effects are also observed when human TNBC cell line MDA-MB-231 is treated with 4SC-202. Colony assays of MDA-MB-231 indicate a significantly reduced number of colonies at concentrations of 100 nM–10 µM (Figure 3a,b) as well as decreased area of colonies from 0.1 µM to1 µM (Figure 3c). Human MDA-MB-231 cells grown within the 3D scaffold also exhibited a significant dose-dependent reduced survivability when treated with increasing concentrations of 4SC-202 for 3 days (Figure 3d). For these scaffolds, repeated measures ANOVA with a post-hoc Dunnett’s test indicate that for concentrations over 10 µM, there is a significant decrease in survival with adjusted *p* < 2 × 10^−16^.

### 3.3. 4SC-202 Treatment Inhibits Cancer Cell Migration in Culture

**Figure 4:**
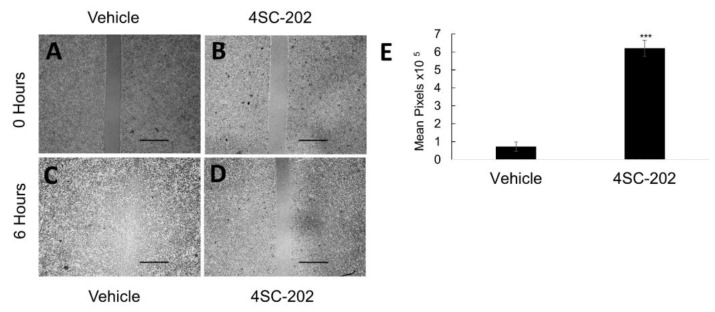
4SC-202 significantly decreases 4T1 cell migration in scratch assays. Scratch assays were performed on confluent cultures of 4T1 that had either been treated with DMSO (**A**,**C**) or 4SC-202 (**B**,**D**). Cultures were photographed at 0 h (**A**,**B**) and 6 h (**C**,**D**) after scratch removal. (**A**–**D**) is imaged with the EVOS XL, scale bar = 2 mm. (**E**) Mean area of each replicate within the scratch region after 6 h (*n* = 3). Significance is based on a two-tailed *t*-test assuming equal variance (adj *p* < 0.001 ***).

Since it is of paramount importance that potential anti-cancer therapeutics block the ability of cancer cells to migrate in order to interfere with metastasis, we examined the in vitro effect of 4SC-202 on cell migration. In vitro scratch assays (Figure 4) show a significant decrease in cell migration in the 4SC-202-treated 4T1 cell culture as compared to the vehicle DMSO control. Cells were photographed at 0 and 6 h after scratching. Measurement at 6 h was taken to differentiate between cell migration and proliferation. The measurements within the scratch region were recorded and the mean area was calculated. There was increased mean area after scratching (*p* = 0.0004) in the 4SC-202-treated, as compared to the vehicle, suggesting a significant decrease in cell migration as early as 6 h after the scratch. Time-dependent proliferation experiments in 4T1 indicate that after 6 h no significant changes in cell proliferation between the control and 4SC-202 treated samples were observed, according to a two-tailed *t*-test, (*p* = 0.44) (Supplementary Figure S2). In addition, based on the doubling time and growth curves of 4T1 cells, no significant change in proliferation was observed after 6 h; thus, the pronounced scratch visible at 6 h in the 4SC-202 treated samples must be due to a lack of migration. 

### 3.4. 4SC-202 Treatment Reduces Tumor Growth and Metastasis in a Murine Model of TNBC

**Figure 5:**
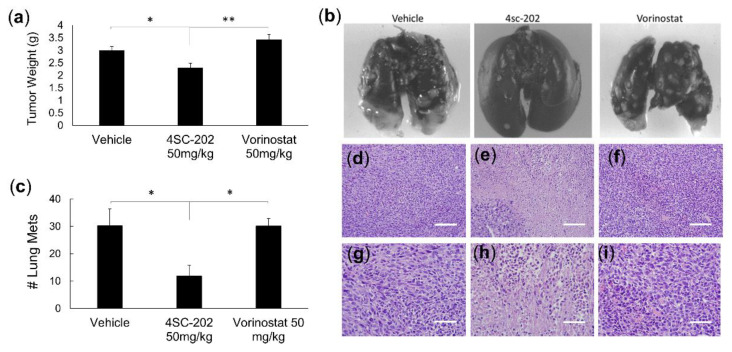
4SC-202 significantly reduces both tumor weight and lung metastasis. (**a**) 4SC-202 (50 mg/kg) significantly reduces tumor weight after 2 weeks of treatment compared to Vorinostat (50 mg/kg) and the vehicle control treatment (ANOVA *p* = 0.005). (**b**) Fewer metastatic nodules in the lungs are visible in 4SC-202 treated mice as compared to Vorinostat- and vehicle-treated mice. (**c**) Quantification of lung metastases indicates 4SC-202 significantly reduces the number of lung metastases as compared to Vorinostat and the vehicle treatment (ANOVA *p* = 0.02). For A and C, hypothesis testing was conducted using single factor ANOVA with a post-hoc Tukey’s test. Significance between groups is indicated *p* < 0.05 (*), *p* < 0.01 (**). (**d**–**i**) H&E staining of tumor sections from mice treated with 4SC-202 (**e**,**h**)**,** Vorinostat (**f**,**i**) or vehicle control (**d**,**g**) indicates 4SC-202 induces marked dissolution of chromatin or karyolysis (**e**,**h**). All data were collected from mice in Experiment 2. Images are representative of 12 tumors analyzed by H&E. Magnification in (**d**–**f**) is 100× with scale bar = 25 microns., and in (**g**–**i**) is 200× with scale bar = 50 microns.

Since the in vitro data indicated that 4SC-202 was both cytotoxic and cytostatic to TNBC cell lines but had no effect on the growth of healthy epithelial breast cells and significantly reduced the ability of the cancer cells to migrate, the next question to be addressed was how the drug affected tumor growth and metastasis in vivo, which more accurately represents the tumor physiology and microenvironment existing in human TNBC patients. To evaluate the effect of the drug in vivo, we investigated how TNBC tumor bearing mice responded to drug treatment in terms of tumor growth and metastasis. We used the highly metastatic 4T1 breast cancer mouse model, which closely mimics human TNBC [23]. We compared the effect of 4SC-202 to Vorinostat, a broad spectrum HDAC inhibitor which is currently used to treat cutaneous T-cell lymphoma, on tumor growth and metastasis. Here we demonstrate that treatment of mice carrying 4T1 tumors with 4SC-202 (50 mg/kg) reduced tumor growth as compared to the vehicle control, but there was a not a significant difference, according to a two-tailed *t*-test assuming unequal variances with *n* = 10 in each group (Supplementary Figure S3, *p* = 0.08). It is feasible that with a higher n or a longer study this difference in tumor volume would be significant. When tumors were excised and weighed, 4SC-202 did significantly reduce the size of the tumors (Figure 5a) as compared to the vehicle control (*p* = 0.03) and Vorinostat (50 mg/kg) treatment (adj *p* = 0.006) according to a post-hoc Tukey’s test (Figure 5a). Thus, 4SC-202 significantly reduces TNBC tumor burden, while Vorinostat has no significant effect.

In addition to significantly reducing the tumor burden in the highly metastatic 4T1 model, 4SC-202 significantly reduced metastasis of the cancer to the lungs (Figure 5b,c). After treatment of 4SC-202, lungs from BALB/c mice carrying 4T1 tumors showed a quantifiably significant reduction in the number of lung metastases relative to the vehicle (*p* = 0.03) and Vorinostat (*p* = 0.02) according to a post-hoc Tukey’s test. Histologically, 4SC-202 had a marked effect on cell health. Hematoxylin and Eosin (H&E) staining of tumor treated with the vehicle, 4SC-202, or Vorinostat indicates that only the 4SC-202-treated tissue has marked dissolution of chromatin or karyolysis (Figure 5d–i). Overall 4SC-202 significantly reduces tumor burden and metastasis and causes marked disruption to the tumor architecture.

### 3.5. 4SC-202 Treatment Decreases 4T1 Population with Cancer Stem Cell Characteristics in 3D Cell Culture

**Figure 6:**
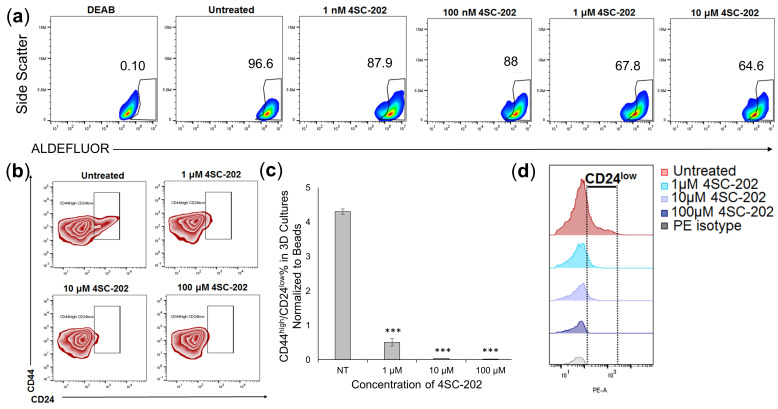
4SC-202 reduces ALDH^high^ and CD44^high^/CD24^low^ cell populations. (**a**) 4T1 cells were treated with 4SC-202 at the indicated concentrations in the 3D scaffolds and analyzed by flow cytometry for a CD44^high^/CD24^low^ cancer stem cell (CSC) profile. (**b**) Contour plot representing gating strategy, and (**c**) quantification of CD44^high^/CD24^low^ CSC population normalized to beads. Significance is tested using ANOVA followed by a Dunnett’s test for *n* = 3 scaffold experiments (*p* < 0.001 ***). (**d**) Histogram plot revealing loss of CD24 expression with increased treatment.

Because our past studies on other cancer types show potentially specific effects of 4SC-202 on cancer stem-like cells [4], the effect of 4SC-202 treatment on particular populations of cells was examined. The effect of 4SC-202 on 4T1 Cancer Stem Cells (CSCs) was investigated by identifying markers of CSCs. In breast cancer, a marker for cancer stem cells is ALDH fluorescence, with high ALDH levels indicative of the cancer cell population [24]. In untreated control cells, nearly 97% of the cell population was ALDH positive. Higher concentrations of 4SC-202 reduced the ALDH^high^ population, as assessed by flow cytometry (Figure 6a). Approximately 5% of the total 4T1 population grown in the 3D scaffolds revealed a distinct Cancer Stem Cell (CSC) profile (CD44^high^/CD24^low^) [25,26], which significantly decreased after 4SC-202 treatment (Figure 6b,c). CD24 expression in the CD44^high^ population is reduced with increasing concentrations of 4SC-202 (Figure 6d). These findings suggest that 4SC-202 significantly reduces the CSC population.

### 3.6. 4SC-202 Treatment Modifies Biological Processes Related to Cancer and Cellular Movement

In order to clarify the molecular origins of the phenotypic changes observed in models of TNBC with respect to cell proliferation and migration, we conducted a bioinformatic analysis of the genes differentially expressed in 4SC-202-treated tumors, relative to control and Vorinostat-treated tumors. Our experimental approach is outlined in Supplementary Figure S1 and is designed to identify 4SC-202-induced perturbations in the transcriptomic landscape that are robust to changes in the experimental dosing scheme, differential expression pipeline, and downstream analysis technique. In brief, RNA-sequencing libraries were built from 4T1 mouse tumors from two separate biological experiments (Experiments 1 and 2). Each of these two experiments were run through three separate bioinformatic workflows (WF 1, 2, and 3). These three different pipelines were run to account for method specific variation in calling DEGs [27,28]. A consensus differentially expressed gene (DEG) set from different pipelines is known to minimize the total number of DEGs; however, this approach ensures higher specificity and hence fewer false positives [27]. For the 4SC-202 versus Vorinostat comparison, this approach identified 33 consensus DEGs; the direction and value of the log fold change is consistent across all three workflows, and about equally split between overexpressed and underexpressed genes (Supplementary Figure S4A,B). 

**Figure 7:**
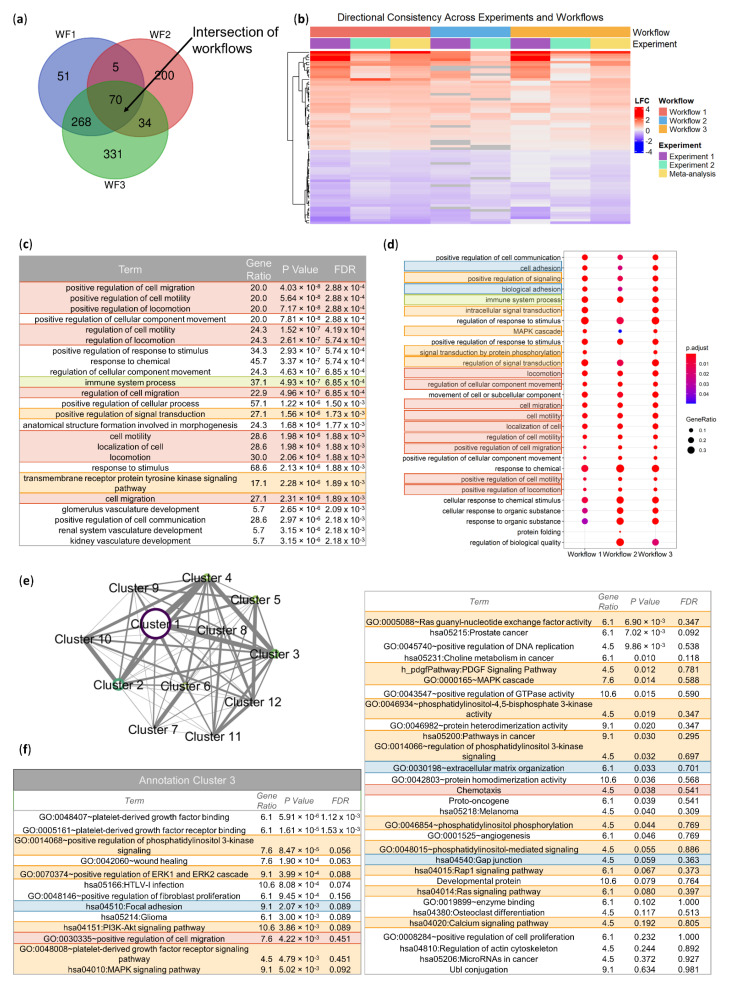
4SC-202-induced differentially expressed genes are implicated in biological processes related to cell adhesion (blue), movement (red), immune processes (green), and cancer-related signaling pathways (orange). (**a**) Venn diagram of the intersection of the differentially expressed genes (DEGs) between workflows. (**b**) Directional consistency heatmap of the log2 fold change (LFC) between 4SC-202-treated tumors and vehicle-treated tumors for consensus DEGs across workflows. Annotations along the top of the heatmap indicate the workflow and experiment of origin. (**c**) Table of top 25 overrepresented GO biological processes for the consensus DEGs corrected for gene length using goseq. (**d**) Dotplot of the consensus enriched pathways across the three workflows. Adjusted *p*-value is indicated by dot color and the ratio of genes mapped to the pathway relative to (approximately) the total number of significant genes is indicated by the size of the dot. (**e**) Visualization of the DAVID functional annotation clusters of the pathways enriched in the 70 consensus DEGs. Node size and color is indicative of the cumulative enrichment score for the cluster, with larger nodes indicating a higher enrichment score. Edge weight is indicative of the extent to which the gene lists of the enriched terms overlap for the gene list of interest. More similar enrichment clusters (nodes) have a higher edge weight than poorly related clusters. (**f**) Enriched terms in the third DAVID functional annotation cluster. Gene ratio is expressed as a percent. FDR indicates the false discovery rate. Coloring of enriched terms on (**c**,**d**,**f**) indicates manually annotated groups of terms of interest.

Each pipeline also utilized a consistent data integration strategy for the 4SC-202 versus control contrast across biological experiments to improve the statistical power of the analysis without losing robustness. In the intersection of these three DEG sets, 70 genes were identified as consensus DEGs (Figure 7a). From the 70 consensus genes, the directionality of the log fold change (LFC) is highly consistent between biological experiments 1, 2, and the meta-analyses (Figure 7b). The magnitude of the log fold change varies between biological experiments and bioinformatic workflows, though there are still visible patterns, particularly in top underexpressed and overexpressed genes (Supplementary Figure S5). Interestingly, among the consensus differentially expressed genes, there were no HDAC-family genes including the genes corresponding to 4SC-202 targets. Furthermore, these genes were not differentially expressed in any of the bioinformatic workflows across the two biological experiments and meta-analyses (Supplementary Figure S6). The gene set analysis of these 70 consensus genes reveals a modulation of the transcriptomic and systems biology landscape, providing several clues to the drug’s mechanism of action. 

Similar to the DEG identification approach, three enrichment tools with unique strengths were used to identify robustly overrepresented biological processes across the datasets: goseq (Figure 7c), clusterProfiler (Figure 7d), and DAVID functional annotation clustering (Figure 7e,f). Goseq was selected to adjust for length bias intrinsic to RNA-sequencing data DEG results when testing for Gene Ontology (GO) biological processes term overrepresentation in the 70 consensus DEGs [29]. Of the 25 most significantly enriched terms, a plurality was related to cell movement (red), though an immune processes term (green) and signaling pathway terms (orange) also appeared (Figure 7c). 

Additionally, the tool clusterProfiler was used to examine the consensus among GO biological process enrichment terms across bioinformatic meta-analysis workflow DEG lists. Instead of testing the consensus DEGs, clusterProfiler tests enrichment for the full set of DEGs for each workflow and then visualizes the consistency between workflows at the biological process level. These enrichment results indicate that there is consistency between the workflows not only at the gene level, but also at the biological process level (Figure 7d). Despite the differences in the size and content of the DEG sets across the bioinformatic workflows, the enrichment profile is quite similar in terms of both the enriched terms and their significance. In this enrichment analysis, terms related to cell movement also figure prominently (red), though terms related to the extracellular matrix (blue), immune processes (green), and MAPK signaling (orange) are also present.

Finally, DAVID functional annotation clustering was used to identify functionally related pathways, processes, and cellular components among others across ontologies, rather than examining just one ontology. Included terms have a more generous *p*-value cutoff than allowed for in other analyses to promote the formation of richer annotation clusters. For the consensus 70 DEG set, DAVID identified 11 clusters of enriched terms (Supplementary File S2). Of these clusters, the DEGs driving the enrichment are closely related, especially for Clusters 1–4 (Figure 7e). These first annotation clusters contain terms related to glycosylation and disulfide bonds (Cluster 1), immunoglobulin (Cluster 2), and transmembrane and plasma membrane regions (Cluster 4) (Supplementary File S2). Annotation Cluster 3 includes a variety of terms, including those related to the extracellular matrix (blue) and cell movement (red). Additionally, Cluster 3 prominently includes signaling pathways, including those commonly implicated in cancer (orange). 

The resulting enrichment analyses showed notable consistency across multiple overrepresentation analysis methods, DEG sets, and enrichment databases. Specifically, terms related to cell movement (red), the extracellular matrix (blue), immune process (green), and signaling networks (orange) were consistently enriched across methods. Of these, terms related to cell movement were the most regularly enriched. Genes annotated to cell motility and two daughter processes (cell migration and positive regulation of cell migration) included genes that had positive and negative log fold changes for the 4SC-202 versus control contrast (Supplementary Figure S7), but the genes in these terms were insufficiently different to separate out drug-treated and control clusters. Collectively, these results suggest that there are biological process terms related to cell motility, the extracellular matrix, immune processes, and cancer signaling networks that are robustly overrepresented in the consensus 4SC-202-induced DEGs, but the data do not support that all genes in the investigated pathways significantly contribute to these changes nor do they conclusively indicate the directionality of the changes. 

Complementary to these GO biological process overrepresentation analyses, a core Ingenuity Pathway Analysis was run to identify enriched canonical pathways and diseases and functions (Supplementary File S4). Among the predicted toxicological functions, there were some significant terms related to cardiotoxicity, hepatoxicity, and nephrotoxicity. The Hepatic Fibrosis/Hepatic Stellate Cell Activation canonical pathway was enriched (*p* = 1.32 × 10^−6^) as well as morphology-related disease and biological functions. For a majority of these terms, the directionality of the change is ambiguous. 

Additionally, the top diseases and biological function terms again included many related to cell movement and migration. Significantly, the Metastasis term appears in the top 20 terms with an enrichment *p*-value of 1.55 × 10^−8^ and a z-score −0.759. While the absolute value of the z-score of activation does not meet the traditional IPA cutoff off two to indicate significance, the direction indicates that the cumulative evidence tends towards a negative effect on metastasis. The genes motivating this negative z-score include overexpression of *PDGRFB*, *CLEC6A*, *TGFBR3* and *SOD3*, and the underexpression of *FOXC2*, *NBEAL2*, *IL23A*, *ID3*, and *IBSP*. The key evidence opposing this negative trend (indicating an increase in metastatic activity) is an overexpression of *PDGRFA*, *S100A4*, *TLR2* and *IL1R1*, and the underexpression of *EPHB6*. These key genes also appear in the top three IPA networks, which are implicated in processes including cancer, cellular movement, and organismal injury and abnormalities (Figures S9–S11). 

**Figure 8:**
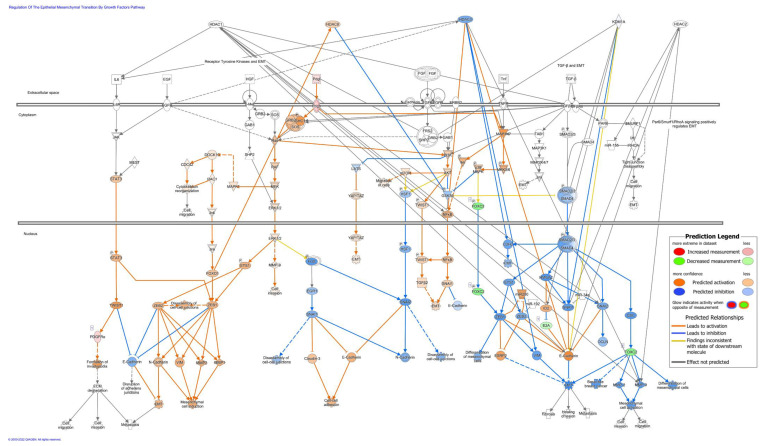
IPA-predicted activation and inhibition of the enriched regulation of epithelial mesenchymal transition by the growth factors pathway based on 4SC-202-induced consensus DEGs. The pathway is built in Ingenuity Pathway Analysis (IPA) and illustrates the known molecular relationships. The molecules are the nodes, and the edges represent biological relationships between these nodes supported by at least one reference from literature, a textbook, or canonical information in the Ingenuity Knowledge Base. 4SC-202-induced consensus differentially expressed genes (DEGs) driving the predicted changes are filled red (overexpressed) or green (underexpressed) with intensity based on the log fold change from Workflow 1. Molecular Activity Predictor (MAP)-based activations are shown in orange and inhibitions in blue. Molecules with no color have no predicted activity or significant differential expression. HDAC1, HDAC2, HDAC3, HDAC8, and KDM1A were added to the network, and their known interactions based on the Ingenuity Knowledge Base are indicated by arrows. Shapes of nodes represent the functional class of the gene product. A more detailed legend for node shapes and relationships can be found at https://qiagen.secure.force.com/KnowledgeBase/articles/BasicTechnicalQA/Legend, accessed on 23 March 2022.

In the top canonical pathways, the regulation of the epithelial mesenchymal transition by the growth factors pathway (Supplementary File S4, Canonical Pathways tab) is also enriched with a *p*-value of 1.78 × 10^−5^. An IPA-based prediction of the molecular activity of this pathway dependent on the directionality of the log fold change of the 4SC-202-induced DEGs suggests that the activity of this signaling pathway may be altered in 4SC-202-treated mice relative to the control (Figure 8). In both the EMT-related pathway (Figure 8) and the cancer- and cell movement-related networks (Supplementary Figures S9–S11), activity prediction indicates a mixture of activation and deactivation of subnetworks based on the 4SC-202-induced gene expression changes. Interestingly, the predicted deactivation of the EMT-related pathway appears to be principally supported by the underexpression of *FOXC2* (Supplementary Figures S5 and S8). This underexpression is consistent between Experiments 1 and 2 (Supplementary Figure S5) showing reproducibility. Additionally, when HDAC 1, 2, 3, and 8 and KDM1A were added to the EMT-related signaling pathway, a majority of the known connections between the targets of 4SC-202 and the signaling network elements were localized in areas of the signaling network that had decreased predicted activity.

To assess the differential effect of 4SC-202 compared to an approved HDACi with a broader set of molecular targets, a similar enrichment analysis approach was taken with respect to the consensus differentially expressed genes for the 4SC-202 versus Vorinostat contrast (Supplementary Figure S4). For this set of genes, there was not as clear of a relationship between the three overrepresentation analyses. While goseq returned some enriched terms, the FDR on all the terms were high. ClusterProfiler was unable to identify any biological processes that were consistently enriched across workflows, and the DAVID functional annotation cluster scores were low (Supplementary Figure S4D, Supplementary File S3). Despite these limitations, there were a few consistencies between the goseq and the DAVID results with respect to terms related to carbohydrate metabolism and mRNA splicing.

An IPA analysis of the consensus genes in the comparison between 4SC-202 and Vorinostat resulted in enrichment of terms related to development, metabolism, and a family of terms related to cell death and survival (*p*-value range 2.30 × 10^−2^–8.48 × 10^−5^) (Supplementary File S5). Interestingly, the first IPA network of densely connected genes is implicated in cellular movement pathways (Supplementary Figure S12). In this network, SOX2 activity is predicted to be decreased. This decrease is principally supported by the underexpression of FAM107B and by the overexpression of LYZ via an inhibitory relationship between SOX2 and ETS2 and ELF3. While the consistency of the enrichment evidence is not as clear for the comparison of 4SC-202 versus Vorinostat, the data may support differences in cell death and survival, and cellular movement.

## 4. Discussion

Here, we demonstrate that the dual HDAC/LSD1 inhibitor 4SC-202 is significantly cytotoxic and cytostatic to TNBC, including in the human TNBC MDA-MB-231, but not to healthy non-cancer breast cells. In addition, the drug inhibits the ability of the TNBC cells to migrate, and significantly reduces the metastasis of the cancer cells to the lungs. On the cellular level, 4SC-202 appears to reduce the Cancer Stem Cell (CSC) population and disrupt the cellular architecture by inducing karyolysis. On the transcriptomic level, 4SC-202 disrupts several cellular processes related to cancer cell migration, but also has pleiotropic effects on non-cancer-related pathways, which could potentially lead to concerning side effects. More rigorous preclinical safety studies need to be conducted in order to consider whether 4SC-202 might be used as a potential adjuvant for TNBC.

Other studies have also demonstrated that 4SC-202 reduces cell viability, induces apoptosis and causes G2/M cell cycle arrest in a wide variety of conditions, including myelodysplastic syndrome (MDS) [30], urothelial carcinoma [6,31], and colorectal cancer [5]. In addition, other studies have demonstrated reduced migration and invasion in vivo in oral squamous cell carcinoma (OSCC) [32], as well as a wide variety of different cancer cell lines, including urothelial carcinoma [6], colorectal cancer cells [5] and highly aggressive pancreatic cancer [8]. On the clinical side, studies have demonstrated administration of 4SC-202 was safe and well tolerated with potential antitumor activity such as in hematological malignancies [33]. Common dysregulation of HDACs occurs in cancers. HDACs are involved in both cell survival and cell proliferation. In the past several decades, there have been a plethora of studies investigating the effect of HDAC inhibitors (HDACi) on tumor growth in a number of different cancers [34]. In addition to influencing gene expression, HDAC enzymes are part of large multi-subunit complexes which have both histone and nonhistone targets affecting signaling in multiple different cellular and system pathways, resulting in a variety of cell-type specific effects [35]. Since HDACs are ubiquitously expressed, inhibitors have pleiotropic cellular effects, such as inducing expression of pro-apoptotic genes and proteins, cellular differentiation, and causing cell cycle arrest [36]. The potential side effects of broad spectrum HDAC inhibition has remained an obstacle in the development of cancer drugs, with numerous side effects observed [37] including nausea/vomiting, diarrhea, and fatigue in thyroid and Cutaneous T-cell lymphoma (CTCL) cancer patients. 4SC-202 differs from clinically available HDAC inhibitors such as Vorinostat (SAHA) by specifically targeting Class I HDACs including HDAC1, HDAC2, and HDAC3 and the histone demethylase LSD1.

Since 4SC-202 affected a population of cells expressing stem cell markers in the brain cancer Atypical Teratoid Rhabdoid Tumor [4], we expected that the drug would also affect the CSC population in breast cancer. In ATRT, it was shown that 4SC-202 decreases a population of cells that overexpress stem cell markers, including *SOX2* and *FOXM1* [4]. 

Likewise, in osteosarcoma, RNA-sequencing data suggested that *SOX2* is underexpressed in 4SC-202-treated cells [38]. Here, in a 3D-scaffold model [13], the population of cells that express stem-like characteristics is small in the 4T1 TNBC model; about 5% of the total 4T1 population grown in the 3D scaffolds revealed a distinct CSC profile (CD44^high^/CD24^low^), which significantly decreased after 4SC-202 treatment (Figure 6b,c). CD24 expression of the CD44^high^ population also showed reduced CD24^low^ expression with increasing concentrations of 4SC-202. Because the population of cells with stem-like characteristics is small relative to the total number of cells, it was unsurprising that stem cell-related genes or pathways did not appear in the enrichment results, especially since the differential expression criteria were so stringent.

Differentially expressed genes were compiled for the comparison of 4SC-202 versus vehicle-only treated mice across three distinct bioinformatic analyses resulting in 70 consensus DEGs. Our focus here was on a highly relevant sub-set of DEGs. This choice was driven by our study’s goal of a preclinical assessment of 4SC-202. The genomic impact of 4SC-202 could involve additional genes, and we provided in our Supplementary Files our full DEGs analysis results for any research work interested in a different genomic signature (Supplementary File S7). 

In this study, the enrichment of terms related to the extracellular matrix and cell movement correspond with the phenotypic changes in cell migration in vitro and total lung metastases in vivo. Other studies have likewise observed a co-occurring enrichment of these cell movement-related terms and phenotypic changes in metastatic activity. In a study of MDA-MB-231, it was demonstrated that GO biological processes associated with cell locomotion, migration, motility, and cellular component movement were enriched for the contrast between the parent cell line and a daughter cell line selected for metastatic potential [39]. Likewise, the GO term “GO:0030335 positive regulation of cell migration” was found to be enriched in the DEGs from a contrast between breast cancer bone metastases and normal samples [40]. Locomotion and cell migration GO terms were also significantly enriched in invasive breast cancer relative to normal tissue, and cell migration was enriched in invasive breast tissue relative to ductal carcinoma in situ [41]. The present study further supports the intuitive connection between the enrichment of these movement-related terms and the invasive and metastatic phenotypes of breast cancer.

Among the pathways in the IPA analysis, the regulation of the epithelial mesenchymal transition by the growth factors pathway and the term metastasis were both enriched. The gene *FOXC2* appears in both terms as a key contributor to a predicted inhibition (negative z-score) of the process and/or downstream elements. In published studies, expression of *FOXC2* has been shown to promote colorectal cancer metastasis [42], is necessary for the full metastatic phenotype of the 4T1 mouse model [43] and is sufficient to promote cancer stem cell-like properties and metastatic activity in transformed human mammary epithelial cells [44]. In our network analyses, there were no direct connections between the targets of 4SC-202 and FOXC2, but FOXC2 is significantly underexpressed (Supplementary Figure S8) and may play a role in the mechanism of action of 4SC-202.

In the IPA network, FOXC2 appears to principally act through interaction with PDGFRB, which is positively regulated by FOXC2 [44]. In our study, PDGFRB and PDGFRA are both significantly overexpressed in 4SC-202-treated tumors. Despite IPA classifying PDGFRB overexpression as support for a negative activation z-score for the metastasis term, there is evidence that overexpression of both PDGFRB [45,46] and PDGFRA [47,48] tends to increase metastasis. The IPA network indicates that PDGFRB and PDGFRA are also implicated in PI3K/AKT/mTOR and Raf/MEK/ERK signaling, which are positively associated with processes including cell growth and proliferation [49]. While the overexpression of PDGRFA/B supports the activation of PI3K/AKT/mTOR and Raf/MEK/ERK, the differential expression of genes upstream and downstream to these pathways such as FOXC2 [50] and JUN [51] indicates an inhibition of these pathways. Because of the broad effects of HDAC inhibitors and this contradictory evidence, it is not clear what the net effect of 4SC-202 is on these key cancer pathways, but it is likely that they are involved in the mechanism of action responsible for the observed cytotoxic and cytostatic effects and decreases in migratory and metastatic activities.

## 5. Conclusions

Increased expression of epigenetic modulators such as *HDAC1* and *LSD1* in human TNBC tumors suggest these may be important targets for therapeutic intervention. As TNBC is highly metastatic, a drug that reduces metastasis would be beneficial for these patients, 4SC-202, a dual selective Class I histone deacetylase (HDAC)/lysine-specific histone demethylase 1A (LSD1) inhibitor, could help prevent relapses that tend to include metastasis. Upon completion of these experiments, 4SC-202 was shown as cytotoxic and cytostatic in vitro in murine TNBC model 4T1, as well as in the human TNBC cell line MDA-MB-231, while showing no effect on the non-cancer breast epithelial cell line MCF10A. Reduction in tumor growth and significant reduction of lung metastasis were shown in vivo in the 4T1 murine model, which closely mimics TNBC in terms of sites of metastasis. When compared to an HDACi that is currently used to treat TNBC patients, Vorinostat, 4SC-202 treatments in vivo reveal a reduction in tumor burden compared to 4T1 mice treated with Vorinostat. 4SC-202 also influences the cancer stem cell population, and the drug’s mechanism of action may involve effects on this population. Approximately 5% of the total 4T1 cell population grown in three-dimensional scaffold had a distinct CD44^high^/CD24^low^ CSC profile which significantly decreased after treatment. A multi-workflow approach with two RNA-sequencing experiments from 4T1 tumors revealed modifications to biological processes related to cancer signaling pathways and cellular movement in 4SC-202-treated tumors. Further studies on the effect of 4SC-202 on these processes may be warranted based on these initial findings. Additionally, since there is evidence that 4SC-202 does modify the cancer stem cell population, more studies that include a more sensitive approach, such as single-cell sequencing, would be beneficial. Preclinical studies of 4SC-202′s efficacy when used in conjunction with a standard of care therapy are also needed.

## Data Availability

The data generated in this study are available upon request. The RNA-sequencing data from the pilot study are publicly available on SRA at PRJNA772749 and the second experiment dataset is available on SRA at PRJNA792996.

## Supplementary Materials

The following supporting information can be downloaded at: https://www.mdpi.com/article/10.3390/cancers14071753/s1 and at https://zenodo.org/record/6392227. File S1 (Figure S1: RNA-sequencing experimental design; Figure S2: Experiments measuring proliferation between drug-treated and control conditions indicate no significant difference 6 hrs. after scratch; Figure S3: Effect of 4SC-202 treatment on 4T1 tumor volume in mice; Figure S4: Differential expression between 4SC-202- and Vorinostat-treated 4T1 tumors; Figure S5: Top underexpressed differentially expressed genes 4SC-202 vs Control; Figure S6: HDACi target genes are not differentially expressed in RNA-sequencing data from 4SC-202-treated mice relative to control mice; Figure S7: Differential expression and expression of genes implicated gene ontology biological processes of interest; Figure S8: IPA visualization of the Regulation of Epithelial Mesenchymal Transition By Growth Factors Pathway emphasizing influence of 4SC-202-induced consensus DEGs; Figure S9: 4SC-202 modulates gene networks related to Cancer, Endocrine System Disorders, and Organismal Injury and Abnormalities; Figure S10: 4SC-202 modulates gene networks related to Cancer, Cellular Movement, and Organismal Injury and Abnormalities; Figure S11: 4SC-202 modulates gene networks related to Cell-mediated Immune Response, Cellular Movement, and Hematological System Development and Function; Figure S12: 4SC-202 differentially modulates gene networks related to Cellular Movement, Hematological System Development and Function, and Immune Cell Trafficking relative to Vorinostat); File S2: DAVID 4SC vs. Control 70DEG results; File S3: DAVID 4SC vs. Vori 33 DEGs results; File S4: IPA 70 All Results; File S5: IPA 33 Summary; File S6: Experiment RIN Numbers; File S7: 4SC vs. Control all DEGs.

## Appendix A

### Supplementary Bioinformatic Methods

For Workflow 1 (WF1), we used STAR (version 2.7.2b) [52] for mapping and DESeq2 (version 1.30.1) [19] for DE analysis in R. To compare the vehicle against 4SC-202 treatment, the two biological experiments were combined and DE analysis was run by controlling for batch effects.

For Workflow 2 (WF2), paired-end FASTQ files were imported into the NetworkAnalyst Galaxy server [53,54]. Mapping and read counting were run with default parameters unless mentioned otherwise. If needed, trimming was of the adapter sequence was performed using Trim Galore! (Galaxy Version 0.4.3.1) proceeded by pseudoalignment using Kallisto quant (Galaxy Version 0.43.1.3) [55] to the human transcriptome index built from GRCh38. The results for each experiment were then put through NetworkAnalyst in the Gene Expression Table (single experiment) or Multiple Gene Expression Tables workflow [53,56,57,58]—the latter included two gene expression tables for meta-analysis. Within the single experiment workflow, DESeq2 was used for differential expression [19]. Within the workflow, autoscaling was used, with no normalization followed by differential expression analysis with limma [59,60]. To combine *p*-values from both experiments Fisher’s method was used with a significance cutoff of adjusted *p*-value of 0.1.

For Workflow 3 (WF3), paired-end FASTQ files from Experimental Runs 1 and 2 were merged (when lanes were retained as individual FASTQ files) and imported into the NetworkAnalyst Galaxy server for mapping and quantification [53,54]. A default workflow was used with default settings except where otherwise noted. Trim Galore! (Galaxy Version 0.4.3.1) [61] was used for adapter sequence trimming (if necessary) followed by HISAT2 (Galaxy Version 2.1.0+galaxy3) [62] for alignment Htseq-count (Galaxy Version 0.9.1) [63] quantified reads per gene_id (Ensembl GRCm38 annotation release 100) and Column Join (Galaxy Version 0.0.3) summarized results. Differential expression was calculated using DESeq2 in R; log fold estimates were shrunk using the apeglm algorithm [19,20]. Prior to the differential expression analysis of integrated datasets from Runs 1 and 2, counts normalized with size factors estimated from the “median ratio method” [19,64] were subjected to surrogate variable analysis using the sva package to identify batch effects between experimental runs. Differential expression was run with surrogate variables 1 and 2 as covariates to the treatment condition since both were significant.

To minimize the effect of gene length on enrichment analysis, goseq (version 1.42.0) [29] was used for conventional KEGG and GO enrichment analysis. Additionally, independent enrichment analysis was conducted on DEGs from each workflow (not consensus lists) using clusterProfiler (3.18.1) [65,66] and visualized as dotplots. For both goseq and clusterProfiler enrichment analyses, all non-zero mapped genes in Workflow 1 were used as the gene universe. Ingenuity Pathway Analysis [67] core analyses on the 70 and 33 consensus DEGs were also used for enrichment analyses and to identify protein-protein interaction networks. All networks were visualized with log fold changes from Workflow 1. IPA analysis was restricted to mammals (human, mouse, and rat). Functional annotation clustering was performed in the Database for Annotation, Visualization and Integrated Discovery (DAVID, 6.8) on the consensus DEG lists mapped to human genes. In total, 66/70 consensus DEGs from the 4SC-202 versus control experiment and 32/32 DEGs from the 4SC-202 versus Vorinostat were successfully mapped (BiomaRt 2.47.7) [68,69]. Clusters were visualized as nodes in a network where node size and color are indicative of the cluster enrichment score and edge weight indicates the overlap in the gene list of the associated terms in Cytoscape (3.7.2) [70]. Heavier weight edges indicate a higher percentage of shared mapped genes between functional annotation clusters. The ComplexHeatmap package (2.0.0) was used to build all heatmaps [71].
