# Supplementary material for: Reduction of Metastasis via Epigenetic Modulation in a Murine Model of Metastatic Triple Negative Breast Cancer (TNBC)
Source: Cancers (Basel). 2022 Mar 30;14(7):1753. doi: 10.3390/cancers14071753 (PMC8996906; doi:10.3390/cancers14071753)
Supplement: Supplementary file 1 [file cancers-14-01753-s001.zip › FileS1_Figures_Proofread.pdf]

cancers MDPI

Supplementary Materials

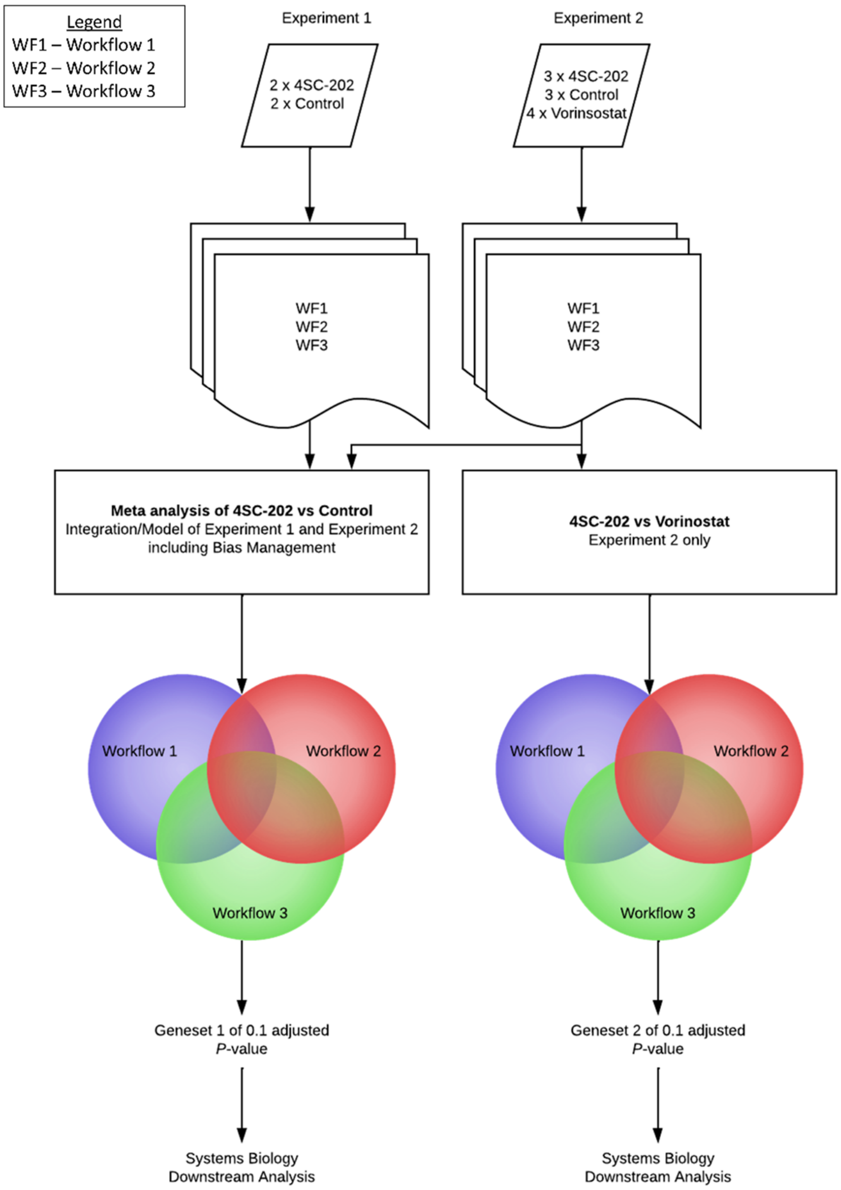

Figure S1. RNA-sequencing experimental design. Two 4T1 *in vivo* experiments were performed for RNAseq analysis. Experiment 1 had four samples, as a pilot study and Experiment 2 had ten. The three pipelines were performed on both experiments (labeled Workflow 1, Workflow 2, and Workflow 3) using a variety of bioinformatics tools. Experiment 1 and 2 were then integrated together separately in each pipeline. DEGs from each pipeline were chosen using significance cutoff of adjusted P-value of 0.1. After curating the DEGs from each pipeline, a Venn diagram was used to find the genes found in all three pipelines. This created a Geneset 1 and Geneset 2. After curation of the Genesets, downstream analysis and systems biology were performed.

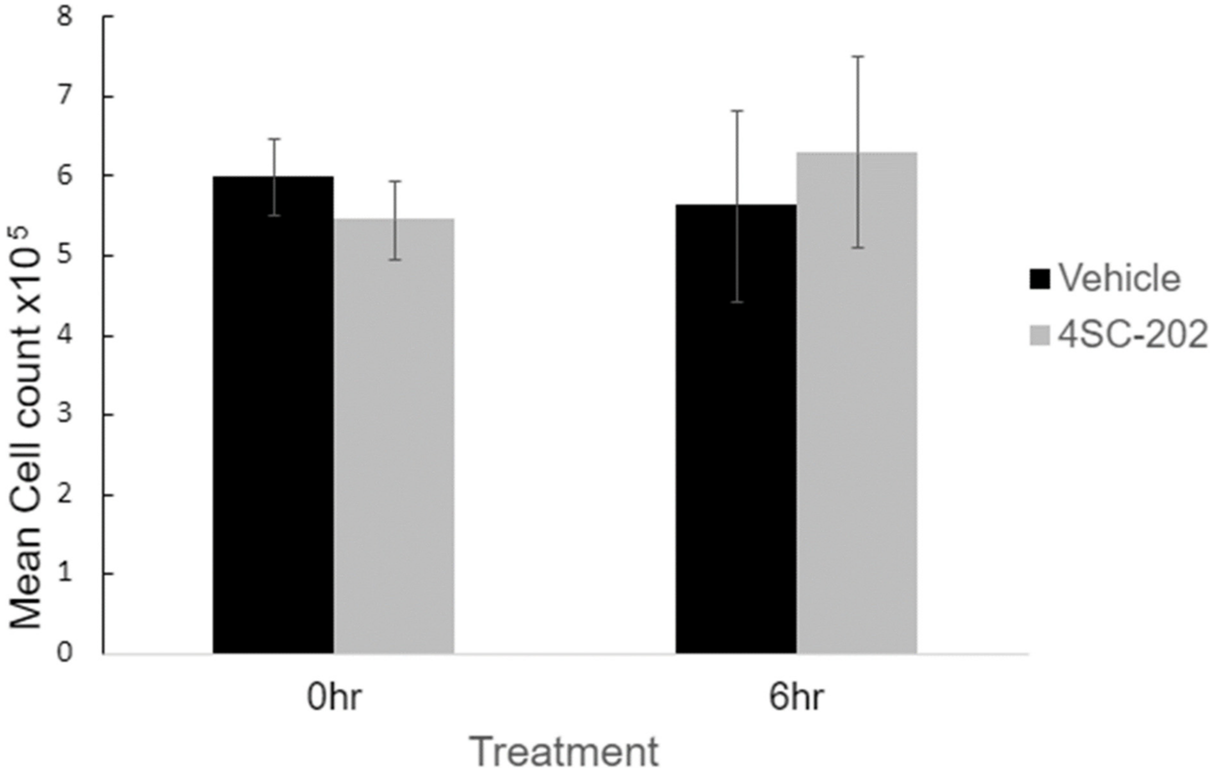

Figure S2. Experiments measuring proliferation between drug-treated and control conditions indicate no significant difference 6 hrs. after scratch. Trypan Blue Cell counting experiments indicate no significant difference in proliferation in cultures with 6 hr. scratch in 4SC-202 as compared to Vehicle at 6hrs. ($p = 0.62$), 0 hr control (p=.21), or 0 hrs. 4SC-202 ($p = -0.09$), according to a paired t-test. Equal amounts of 4T1 were plated on 60 mm dishes. At 0 hr., cell counts with trypan blue to determine live cells were conducted in Vehicle and 0 hr. 4SC-202 treatments, as well as 6 hrs after scratch in Vehicle and 4SC-202 treatment.

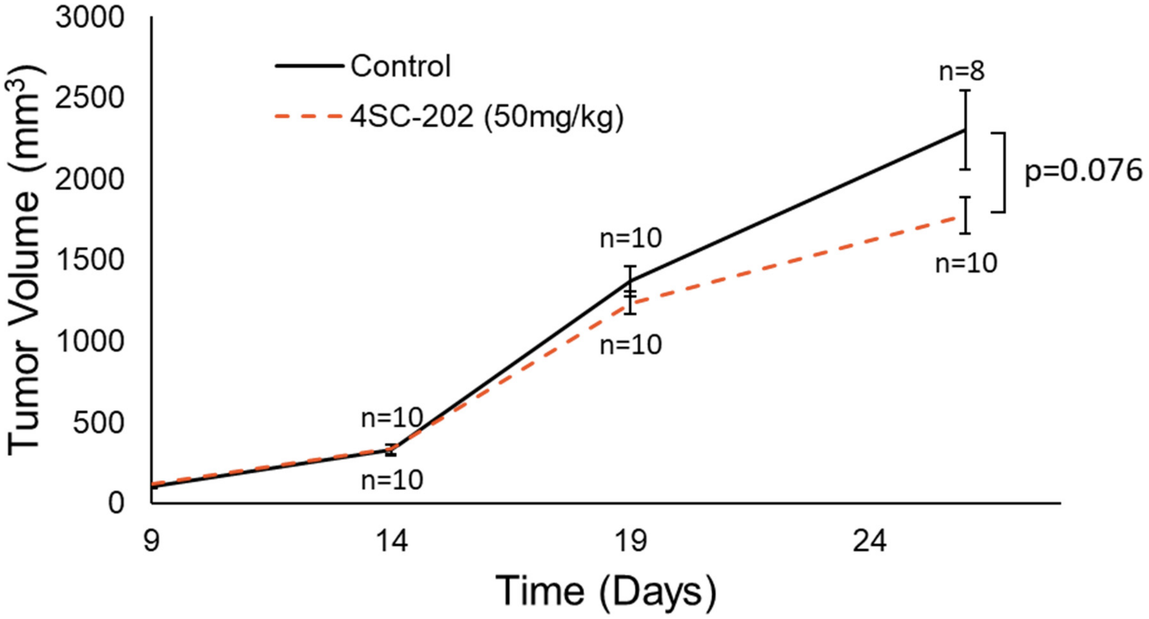

Figure S3. Effect of 4SC-202 treatment on 4T1 tumor volume in mice. There is an increasing difference in the mean tumor volume of 4SC-202-treated and vehicle-treated mice at timepoints 9, 14, 19, and 26 days after tumor seeding (2 weeks of treatment). At 26 days, there is no significant difference between control and 4SC-202-treated group according to a two-tailed t-test, not assuming equal variances (p = 0.076).

A

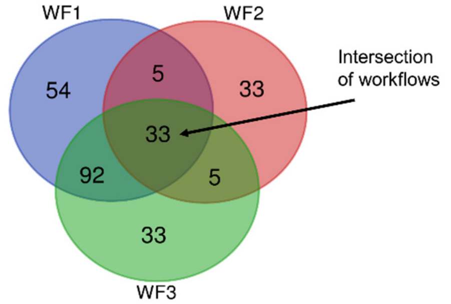

B

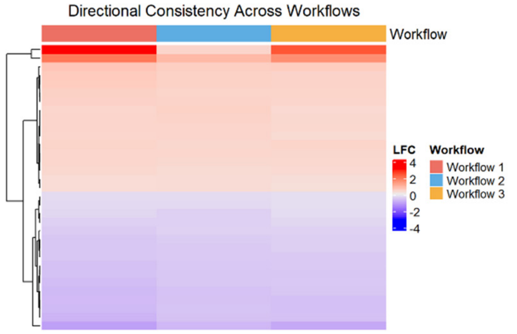

C

Term	Gene Ratio	P Value	FDR
carbohydrate metabolic process	21.2	4.05×10^{-5}	0.895
carbohydrate biosynthetic process	12.1	3.95×10^{-4}	1
RNA splicing	15.2	6.44×10^{-4}	1
epidermal growth factor receptor signaling pathway	9.1	1.14×10^{-3}	1
negative regulation of interleukin-1 production	6.1	1.29×10^{-3}	1
mRNA splice site selection	6.1	1.45×10^{-3}	1
ERBB signaling pathway	9.1	1.47×10^{-3}	1
regulation of epidermal growth factor-activated receptor activity	6.1	1.52×10^{-3}	1
insulin-like growth factor receptor signaling pathway	6.1	2.00×10^{-3}	1
glycogen cell differentiation involved in embryonic placenta development	3.0	2.13×10^{-3}	1

D

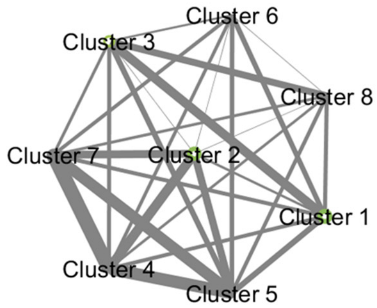

E

Annotation Cluster 1

Term	%	PValue	FDR
Carbohydrate metabolism	12.5	3.78×10^{-4}	0.032
topological domain:Lumenal	15.625	4.31×10^{-3}	0.492
GO:0000139~Golgi membrane	15.625	0.012	0.239
Signal-anchor	12.5	0.031	0.265
Transferase	18.75	0.110	0.718
Golgi apparatus	12.5	0.122	0.736
mutagenesis site	15.625	0.418	1.000

Annotation Cluster 2

Term	%	PValue	FDR
mRNA splicing	12.5	0.007	0.265
mRNA processing	12.5	0.013	0.265
GO:0008380~RNA splicing	9.375	0.027	1.000
GO:0044822~poly(A) RNA binding	12.5	0.250	1.000

Annotation Cluster 3

Term	%	PValue	FDR
GO:0016021~integral component of membrane	46.875	0.012	0.239
transmembrane region	43.75	0.023	1.000
Transmembrane helix	46.875	0.025	0.265
Transmembrane	46.875	0.026	0.265
Membrane	53.125	0.060	0.463
topological domain:Cytoplasmic	31.25	0.060	1.000
glycosylation site:N-linked (GlcNAc...)	31.25	0.165	1.000
Glycoprotein	31.25	0.232	0.966

Figure S4. Differential expression between 4SC-202- and Vorinostat-treated 4T1 tumors. Vorinostat is a current drug therapeutic for breast cancer and is an HDACi, the goal of this experiment was to determine differences between 4SC-202 and Vorinostat based on differentially expressed genes and downstream analysis. RNA-seq libraries were generated from Vorinostat-treated and 4SC-202-treated BALB/c mice with 4T1 tumors. Consensus differentially expressed genes (DEGs) were determined on the basis of three independently run bioinformatics workflows (Workflows 1, 2, and 3). A) Venn diagram of the intersection of the differentially expressed genes between workflows (adjusted p-value 0.1). B) Directional consistency heatmap of the log2 fold change (LFC) between 4SC-202-treated tumors and Vorinostat-treated tumors for consensus DEGs across workflows. Annotations along the top of the heatmap indicate the workflow, all results are from Experiment 2. C) Table of top 10 overrepresented GO biological processes for the 33 consensus DEGs corrected for gene length using goseq. Row colors indicate terms related to DAVID annotation clusters (see E). D) Visualization of the DAVID functional annotation clusters of the pathways enriched in the 33 consensus DEGs. Node size and color is indicative of the cumulative enrichment score for the cluster, with larger nodes indicating a higher enrichment score. Edge weight is indicative of the extent to which the gene lists of the enriched terms overlap for the gene list of interest. More similar enrichment clusters (nodes) have a higher edge weight than poorly related clusters. E) Enriched terms in DAVID functional annotation Clusters 1, 2, and 3. Gene ratio is expressed as a percent. FDR indicates the false discovery rate.

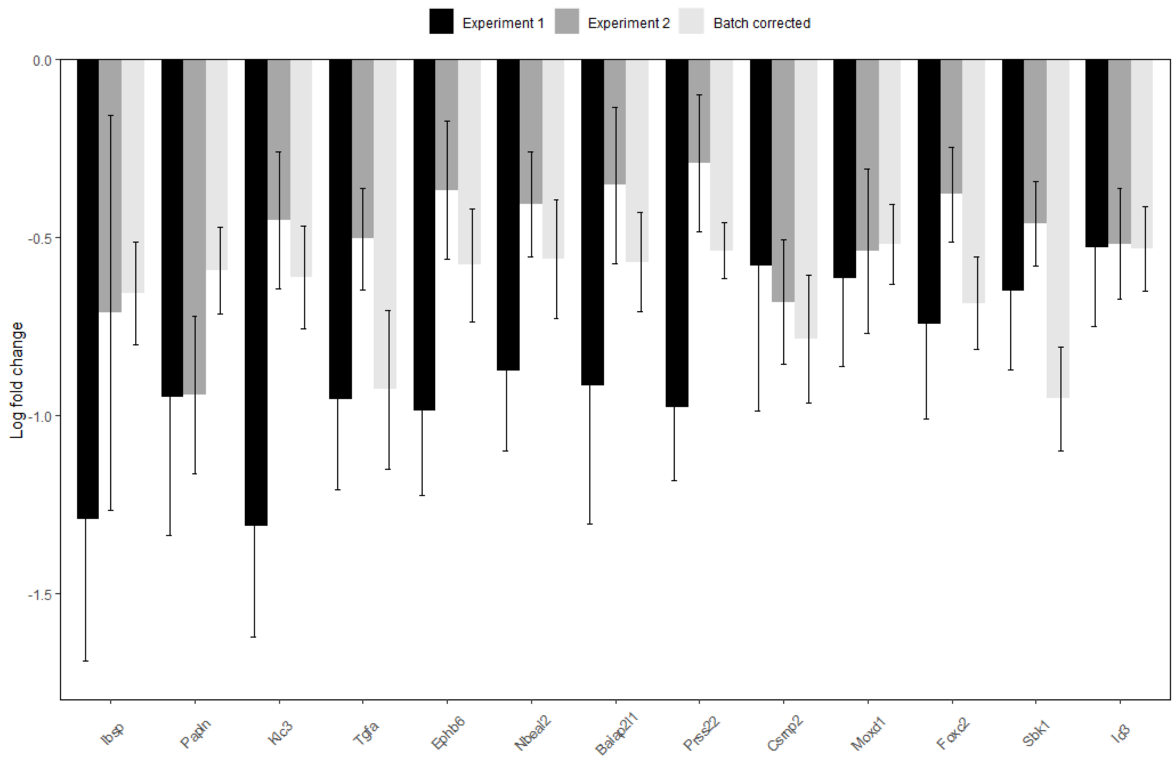

Figure S5. Top underexpressed differentially expressed genes 4SC-202 vs Control**.** Log fold change in Foxc2 and other down regulated genes in the comparison between 4SC-202 and control. Values are estimated log fold change and their standard errors from DESeq2 model fit. Log fold values for Experiment 1 and Experiment 2 are based on separate analysis, while the batch corrected log fold values are based on a DESeq2 model that accounts for batch effects. There is self-validation across the two lab experiments, and it is seen that Foxc2 is underexpressed in 4SC-202 treated tumors.

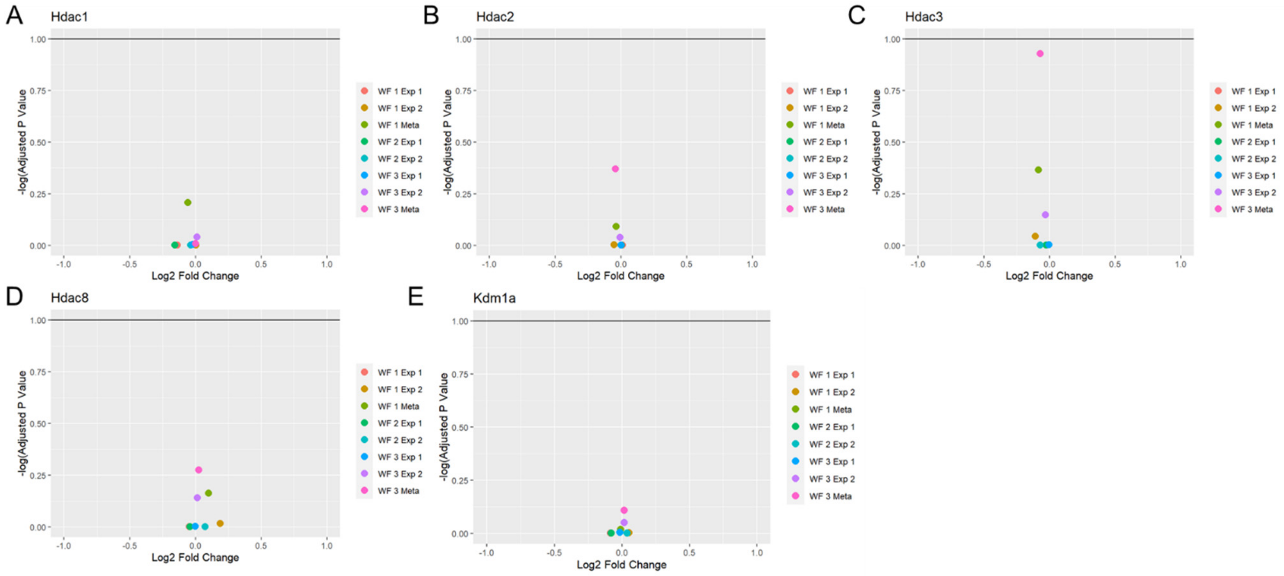

Figure S6. HDACi target genes are not differentially expressed in RNA-sequencing data from 4SC-202-treated mice relative to control mice. Log2 fold change and significance was calculated using Workflows (WF) 1, 2, and 3 for Experiments (Exp) 1, 2, and a meta-analysis of the two experiments for the gene targets of 4SC-202 as described in the Methods section. A-E) Volcano plots of the gene expression of 4SC-202-targets across bioinformatics workflows and biological experiments. Significance threshold (adjusted p = 0.1) is indicated by the solid line at the top of the plot.

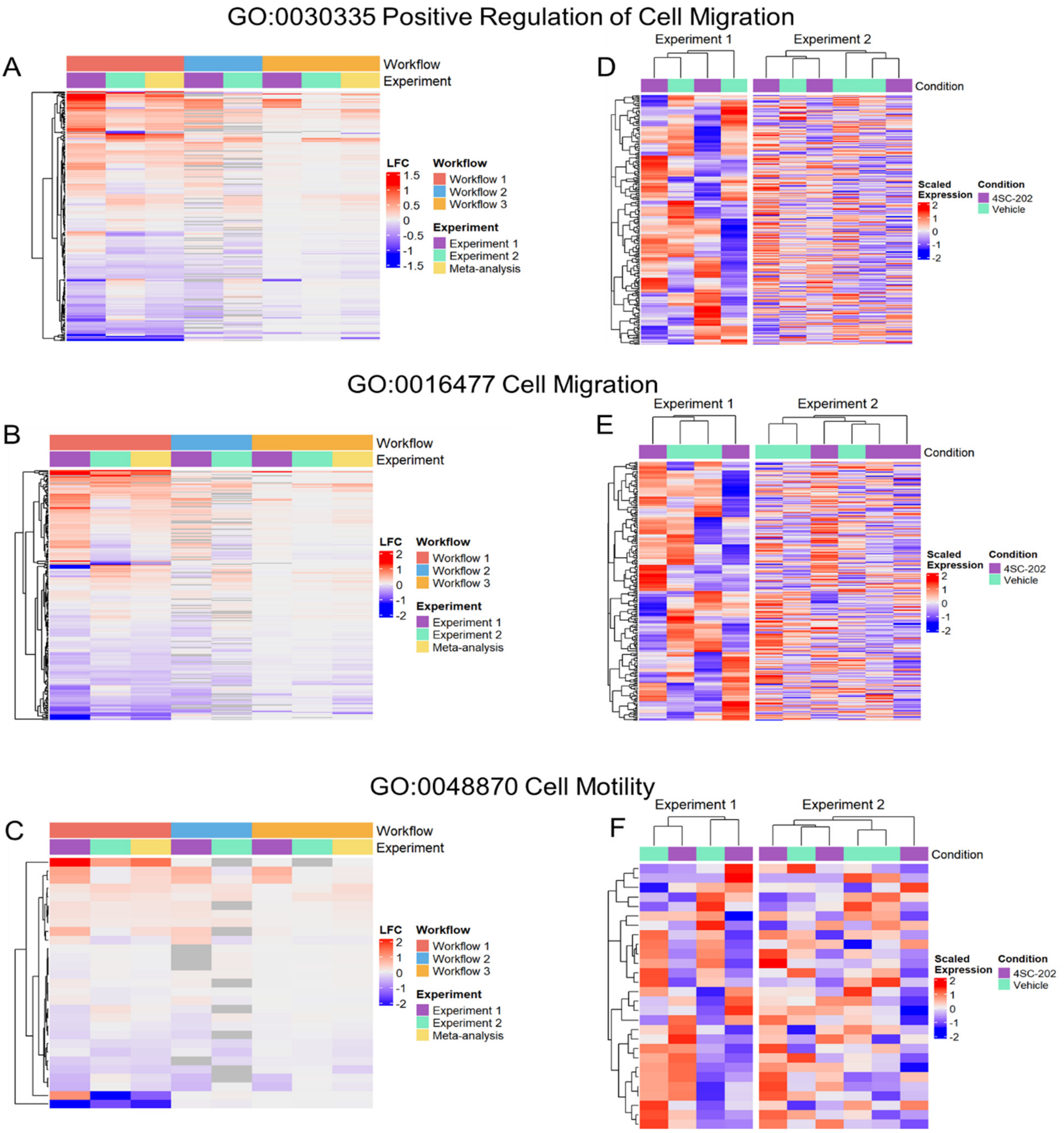

Figure S7. Differential expression and expression of genes implicated gene ontology biological processes of interest. A-C) Gene Ontology heatmap of the log2 fold change (LFC) between 4SC-202-treated tumors and vehicle-treated tumors for genes annotated to indicated GO biological processes. Annotations along the top of the heatmap indicate the workflow and experiment of origin. D-F) Heatmap of normalized gene expression values across Experiments 1 and 2 for GO term of interest. Expression values were mapped and adjusted for library size according to Workflow 3, transformed using variance stabilizing transformation (vst) in DESeq2, and row normalized (standard scaling).

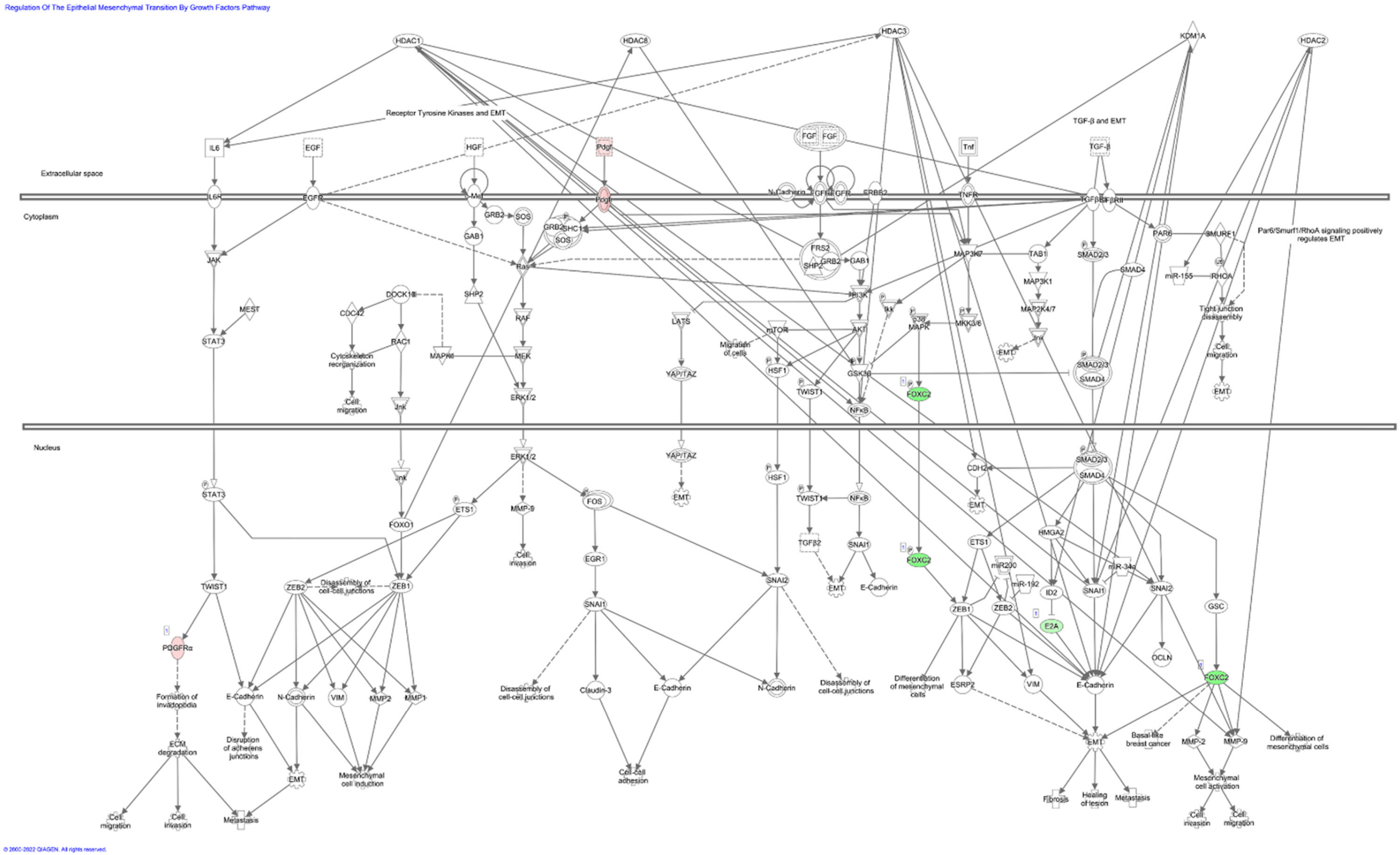

Regulation Of The Epithelial Mesenchymal Transition By Growth Factors Pathway
Extracellular space
Cytoplasm
Nucleus
HDAC1
HDAC8
HDAC3
KDM1A
HDAC2
Receptor Tyrosine Kinases and EMT
TGF-β and EMT
IL6
EGF
HGF
Pdgf
FGF
Tnf
TGF-β
IL6R
EGFR
Met
N-Cadherin
ERBB2
TNFR
TGFβRII
GRB2
SOS
SHC1
GAB1
FRS2
SHP2
JAK
Ras
PI3K
MAP3K7
TAB1
MAP3K1
MAP2K4/7
PAR6
SMURF1
SMAD2/3
SMAD4
miR-155
RHOA
Par6/Smurf1/RhoA signaling positively regulates EMT
Tight junction disassembly
Cell migration
MEST
STAT3
CDC42
DOCK1
RAC1
Cytoskeleton reorganization
MAPK
RAF
MEK
ERK1/2
LATS
YAP/TAZ
mTOR
Migration of cells
AKT
Ikk
p38 MAPK
MKK3/6
Jnk
HSF1
GSK3B
TWIST1
NFκB
FOXC2
EMT
ETS1
FOXO1
MMP-9
Cell invasion
FOS
EGR1
SNAI1
SNAI2
TGFβ2
E-Cadherin
CDH2
HMGA2
miR-200
miR-192
miR-34a
ID2
E2A
ZEB1
ZEB2
GSC
OCLN
TWIST1
PDGFRα
Disassembly of cell-cell junctions
Formation of invadopodia
N-Cadherin
VIM
MMP2
MMP1
ECM degradation
Disruption of adherens junctions
Mesenchymal cell induction
Metastasis
Claudin-3
Cell-cell adhesion
Differentiation of mesenchymal cells
ESRP2
Basal-like breast cancer
MMP-2
Fibrosis
Healing of lesion
Mesenchymal cell activation
Differentiation of mesenchymal cells
© 2000-2022 QIAGEN. All rights reserved.

Figure S8. IPA visualization of the Regulation of Epithelial Mesenchymal Transition By Growth Factors Pathway emphasizing influence of 4SC-202-induced consensus DEGs. The pathway is built in Ingenuity Pathway Analysis (IPA) and illustrates the known molecular relationships. The molecules are the nodes, and the edges represent biological relationships between these nodes supported by at least one reference from literature, a textbook, or canonical information in the Ingenuity Knowledge Base. 4SC-202-induced consensus differentially expressed genes (DEGs) driving the predicted changes are filled red (overexpressed) or green (underexpressed) with intensity based on the log fold change from Workflow 1. Molecules with no color have significant differential expression. HDAC1, HDAC2, HDAC3, HDAC8, and KDM1A were added to the network, and their known interactions based on the Ingenuity Knowledge Base are indicated by arrows. Shapes of nodes represent the functional class of gene product. A more detailed legend for node shapes and relationships can be found at https://qiagen.secure.force.com/KnowledgeBase/articles/Basic_Technical_Q_A/Legend accessed 23 March 2022.

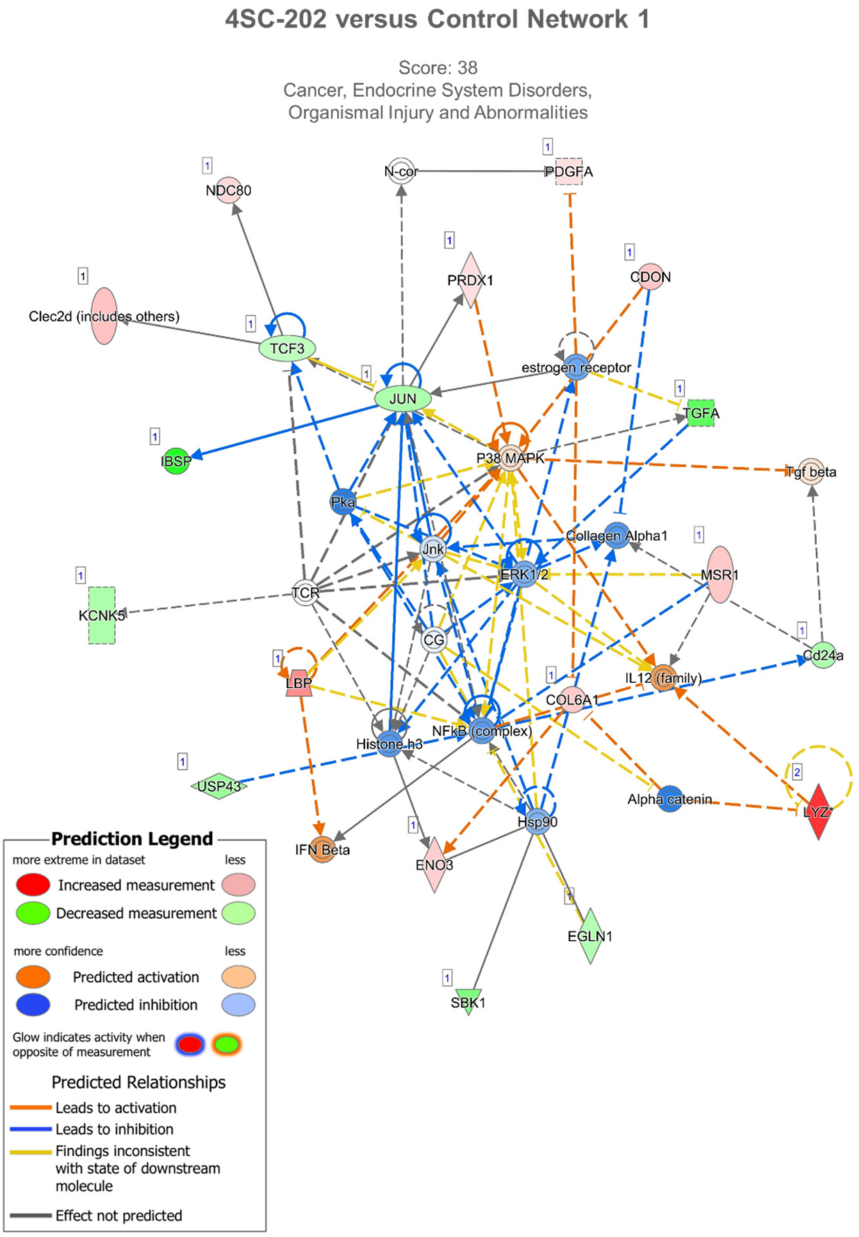

Figure S9. 4SC-202 modulates gene networks related to Cancer, Endocrine System Disorders, and Organismal Injury and Abnormalities. Ingenuity Pathway Analysis (IPA) networks were built from the consensus 4SC-202-induced differentially expressed genes (DEGs). These DEGs were mapped to the IPA Knowledge Base and overlaid onto a global molecular network comprised of known

molecular interactions. Networks were then algorithmically generated based on the density of connectivity. Functionally enriched biological functions and/or diseases most significant to the network were determined to be the three most significantly enriched terms based on a right-tailed Fisher's Exact Test. Network nodes are colored by their log fold change (positive: red, negative: green) or predicted molecular activity (MAP) for non-DEGs (activation: orange, inhibition: blue). Edge colors have similar meanings with the addition of yellow indicating that predicted findings are inconsistent with the state of the downstream molecule. Grey/white nodes and edges indicate no predicted effect.

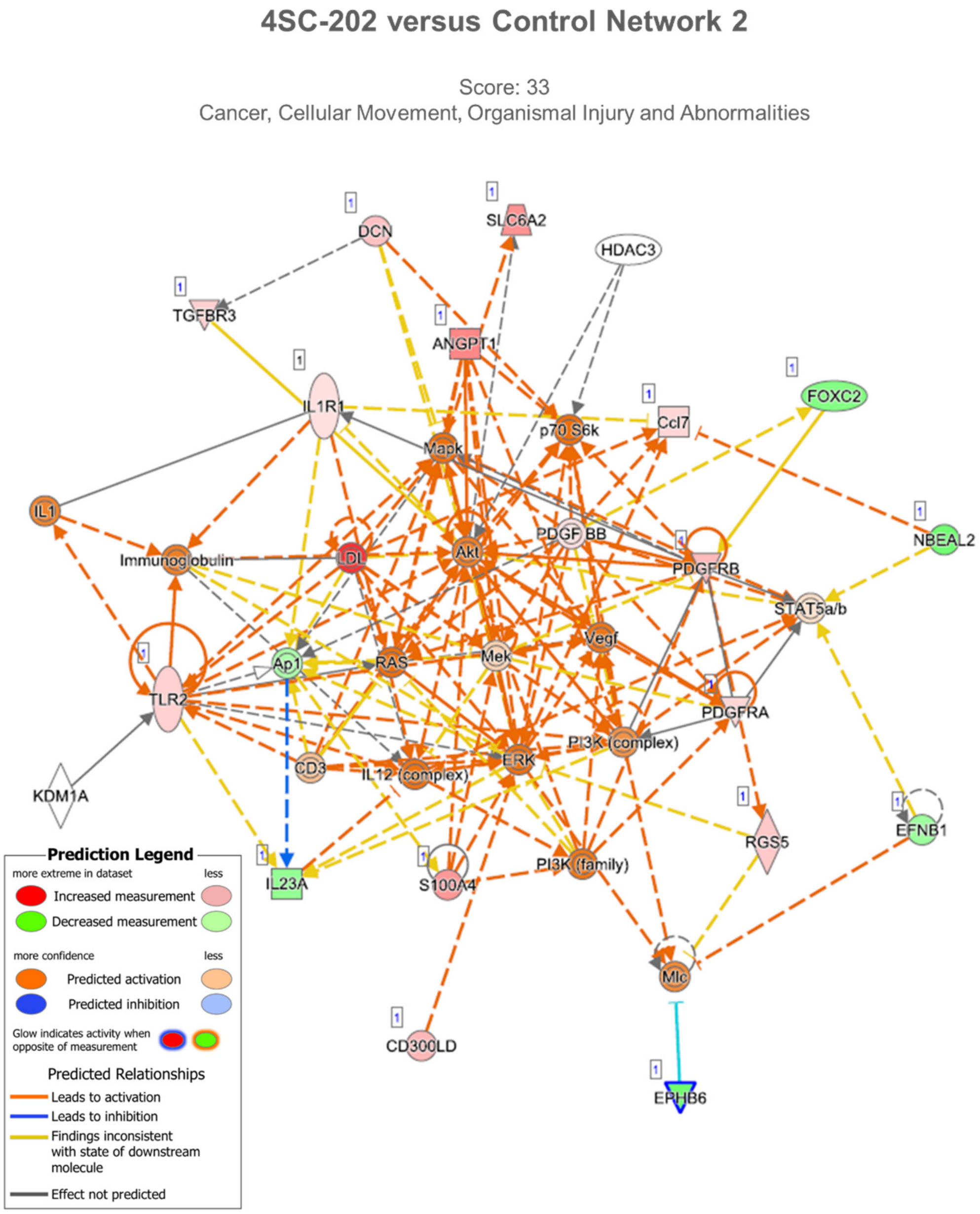

Figure S10. 4SC-202 modulates gene networks related to Cancer, Cellular Movement, and Organismal Injury and Abnormalities. Ingenuity Pathway Analysis (IPA) networks were built from the consensus 4SC-202-induced differentially expressed genes (DEGs). These DEGs were mapped to the IPA Knowledge Base and overlaid onto a global molecular network comprised of known molecular interactions. Networks were then algorithmically generated based on the density of connectivity. Functionally enriched biological functions and/or diseases most significant to the network were determined to be the three most significantly enriched terms based on a right-tailed Fisher's Exact Test. Network nodes are colored by their log fold change (positive: red, negative: green) or predicted molecular activity (MAP) for non-DEGs (activation: orange, inhibition: blue). Edge colors have

similar meanings with the addition of yellow indicating that predicted findings are inconsistent with the state of the downstream molecule. Grey/white nodes and edges indicate no predicted effect.

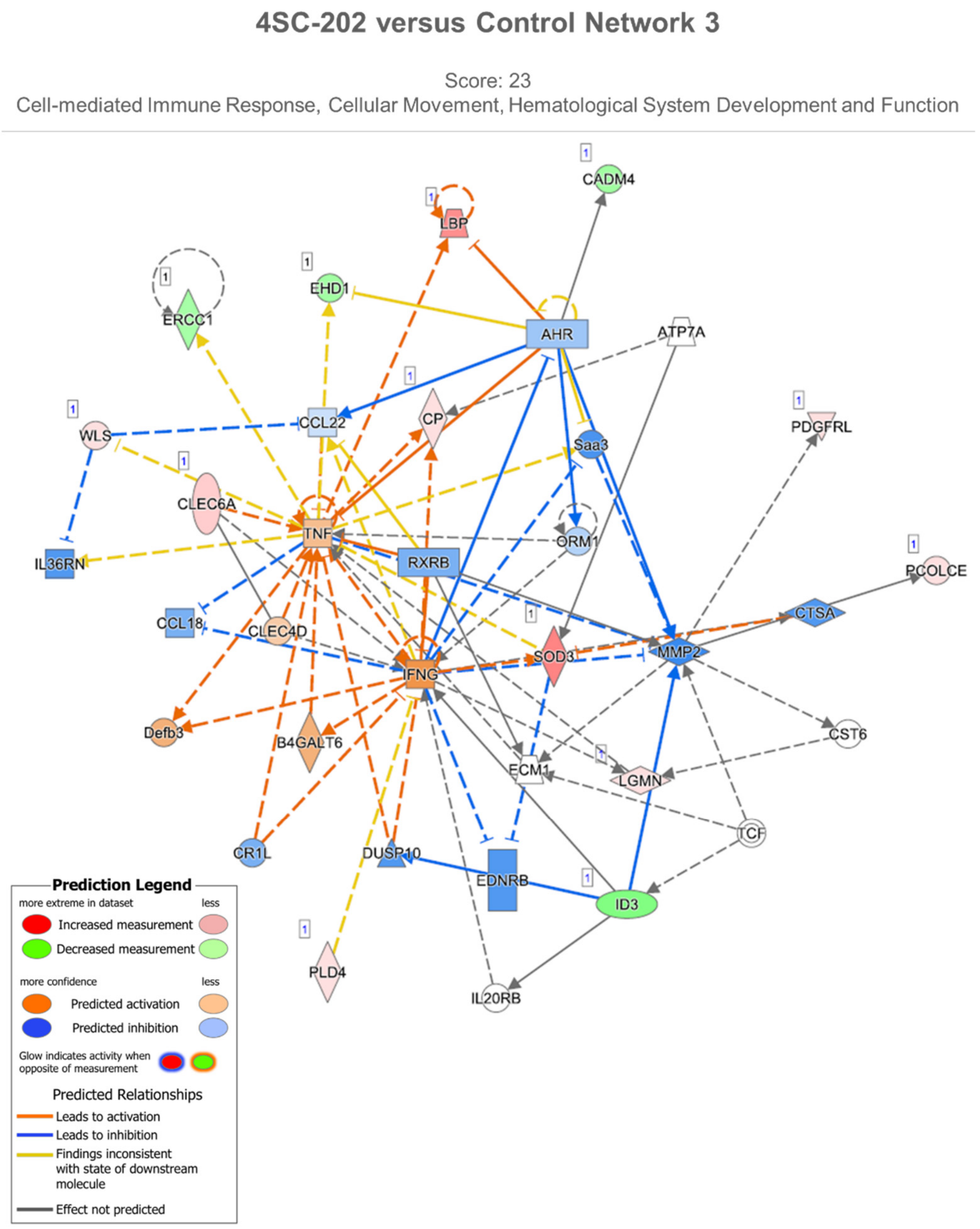

Figure S11. 4SC-202 modulates gene networks related to Cell-mediated Immune Response, Cellular Movement, and Hematological System Development and Function. Ingenuity Pathway Analysis (IPA) networks were built from the consensus 4SC-202-induced differentially expressed genes (DEGs). These DEGs were mapped to the IPA Knowledge Base and overlaid onto a global molecular network comprised of known molecular interactions. Networks were then algorithmically generated based on the density of connectivity. Functionally enriched biological functions and/or diseases most significant to the network were determined to be the three most significantly enriched terms based on a right-tailed Fisher's Exact Test. Network nodes are colored by their log fold change (positive: red, negative: green) or predicted molecular activity (MAP) for non-DEGs (activation: orange, inhibition: blue). Edge colors have similar meanings with the addition of yellow indicating that predicted findings are inconsistent with the state of the downstream molecule. Grey/white nodes and edges indicate no predicted effect.

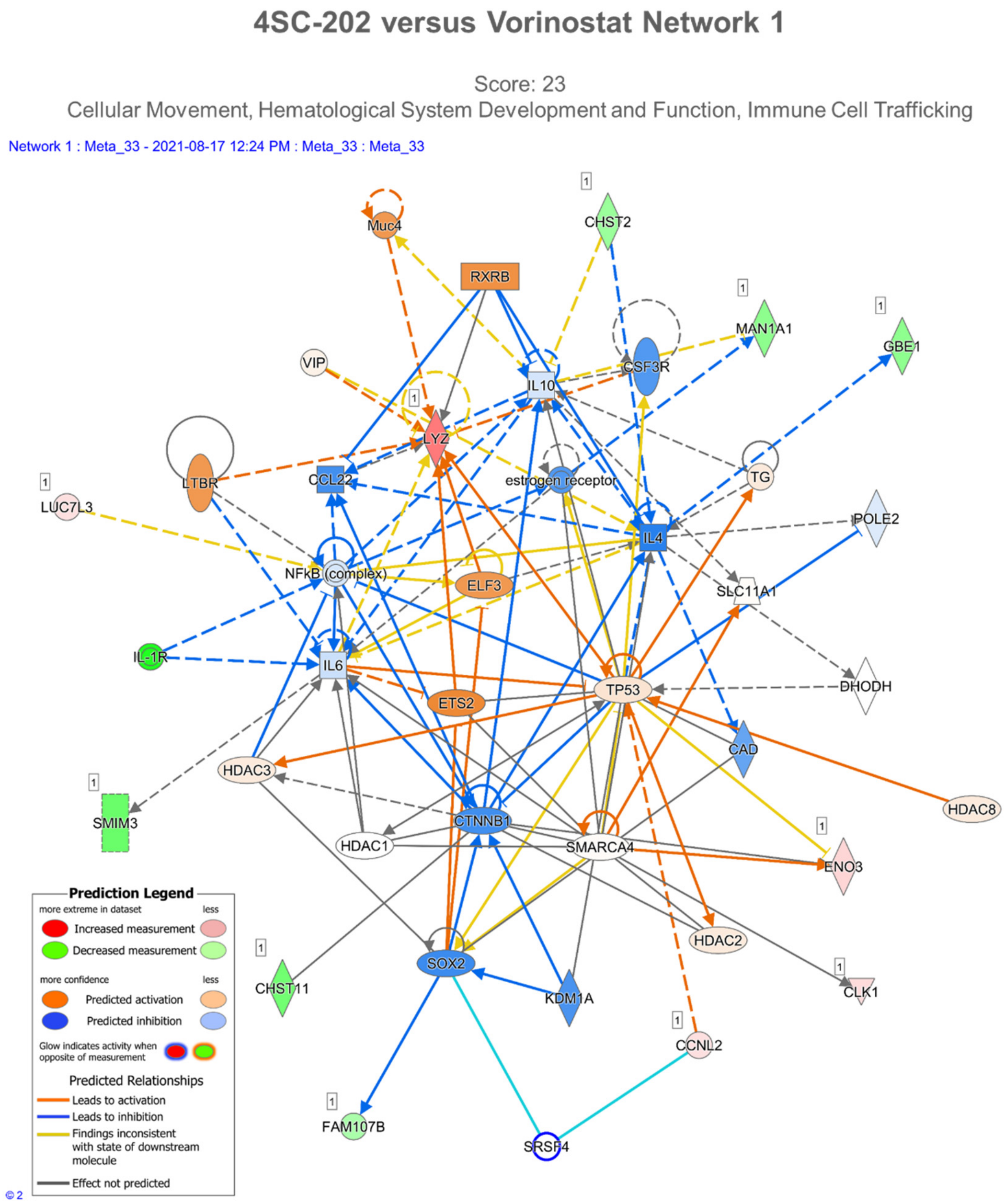

Figure S12. 4SC-202 differentially modulates gene networks related to Cellular Movement, Hematological System Development and Function, and Immune Cell Trafficking relative to Vorinostat. Ingenuity Pathway Analysis (IPA) networks were built from the consensus differentially expressed genes (DEGs) in the 4SC-202 versus Vorinostat contrast. These DEGs were mapped to the IPA Knowledge Base and overlaid onto a global molecular network comprised of known molecular interactions. Networks were then algorithmically generated based on the density of connectivity. Functionally enriched biological functions and/or diseases most significant to the network were determined to be the three most significantly enriched terms based on a right-tailed Fisher's Exact Test. Network nodes are colored by their log fold change (positive: red, negative: green) or predicted molecular activity (MAP) for non-DEGs (activation: orange, inhibition: blue). Edge colors have similar meanings with the addition of yellow indicating that predicted findings are inconsistent with the state of the downstream molecule. Grey/white nodes and edges indicate no predicted effect.
