# Supplementary material for: Reduction of Metastasis via Epigenetic Modulation in a Murine Model of Metastatic Triple Negative Breast Cancer (TNBC)
Source: Cancers (Basel). 2022 Mar 30;14(7):1753. doi: 10.3390/cancers14071753 (PMC8996906; doi:10.3390/cancers14071753)
Supplement: Supplementary file 1 [file cancers-14-01753-s001.zip › FileS5_IPA_33_Summary.pdf]

INGENUITY
PATHWAY ANALYSIS

Analysis Name: Meta_33 - 2021-08-17 12:24 PM
Analysis Creation Date: 2021-08-17
Build version: exported
Content version: 65367011 (Release Date: 2021-06-04)

Experiment Metadata

Name	Value

Analysis Settings

Reference set: Ingenuity Knowledge Base (Genes Only)
Relationship to include: Direct and Indirect
Includes Endogenous Chemicals
Optional Analyses: My Pathways My List

Filter Summary:
Consider only molecules and/or relationships where
(species = Rat OR Human OR Mouse) AND
(confidence = Experimentally Observed) AND
(mol. types = biologic drug OR canonical pathway OR chemical - endogenous mammalian OR chemical - endogenous non-mammalian OR chemical - kinase inhibitor OR chemical - other OR chemical - protease inhibitor OR chemical drug OR chemical reagent OR chemical toxicant OR complex OR cytokine OR disease OR enzyme OR function OR G-protein coupled receptor OR group OR growth factor OR ion channel OR

(c) 2000-2021 QIAGEN. All rights reserved.

kinase OR ligand-dependent nuclear receptor OR mature microRNA OR microRNA OR other OR peptidase OR phosphatase OR transcription regulator OR translation regulator OR transmembrane receptor OR transporter) AND
(data sources = An Open Access Database of Genome-wide Association Results OR BIND OR BioGRID OR Catalogue Of Somatic Mutations In Cancer (COSMIC) OR Chemical Carcinogenesis Research Information System (CCRIS) OR Clinical Genome Resource (ClinGen) OR ClinicalTrials.gov OR ClinVar OR Cognia OR DIP OR DrugBank OR Gene Ontology (GO) OR GVK Biosciences OR Hazardous Substances Data Bank (HSDB) OR HumanCyc OR Ingenuity Expert Findings OR Ingenuity ExpertAssist Findings OR IntAct OR Interactome studies OR MIPS OR miRBase OR miRecords OR Mouse Genome Database (MGD) OR Obesity Gene Map Database OR Online Mendelian Inheritance in Man (OMIM) OR TarBase OR TargetScan Human)

Top Canonical Pathways

Name	p-value	Overlap
**Neuregulin Signaling**	5.82E-04	2.6 % 3/117
**Dermatan Sulfate Biosynthesis (Late Stages)**	1.81E-03	4.4 % 2/45
**Chondroitin Sulfate Biosynthesis (Late Stages)**	1.97E-03	4.3 % 2/47
**Chondroitin Sulfate Biosynthesis**	2.69E-03	3.6 % 2/55
**Dermatan Sulfate Biosynthesis**	2.99E-03	3.4 % 2/58

Top Upstream Regulators

Upstream Regulators

Name	p-value	Predicted Activation

(c) 2000-2021 QIAGEN. All rights reserved.

INGENUITY PATHWAY ANALYSIS

**mir-8**	8.66E-04	
**NDRG1**	1.15E-03	
**Flt1**	1.48E-03	
**BTG3-AS1**	1.48E-03	
**CD38**	1.48E-03	

Causal Network

Name	p-value	Predicted Activation
**GPI**	3.11E-04	
**MAPK3**	4.70E-04	
**mir-8**	8.66E-04	
**NDRG1**	1.15E-03	
**CD19**	1.34E-03	

Top Diseases and Bio Functions

Diseases and Disorders

Name	p-value range	# Molecules
**Developmental Disorder**	1.53E-02 - 5.29E-05	10
**Hereditary Disorder**	2.35E-02 - 5.29E-05	8
**Metabolic Disease**	5.58E-03 - 5.29E-05	5
**Organismal Injury and Abnormalities**	2.35E-02 - 5.29E-05	33
**Skeletal and Muscular Disorders**	1.39E-02 - 5.29E-05	9

(c) 2000-2021 QIAGEN. All rights reserved.

INGENUITY PATHWAY ANALYSIS

Molecular and Cellular Functions

Name	p-value range	# Molecules
Cellular Development	2.41E-02 - 1.14E-05	8
Cellular Growth and Proliferation	2.41E-02 - 1.14E-05	9
Carbohydrate Metabolism	1.80E-02 - 2.09E-05	7
Cell Death and Survival	2.30E-02 - 8.48E-05	8
Cell Signaling	1.30E-02 - 1.24E-04	3

Physiological System Development and Function

Name	p-value range	# Molecules
Cardiovascular System Development and Function	2.35E-02 - 1.14E-05	4
Embryonic Development	2.35E-02 - 1.14E-05	8
Organismal Development	2.35E-02 - 1.14E-05	10
Endocrine System Development and Function	1.39E-02 - 1.97E-04	4
Organ Development	2.41E-02 - 9.17E-04	8

Top Tox Functions

Assays: Clinical Chemistry and Hematology

(c) 2000-2021 QIAGEN. All rights reserved.

INGENUITY PATHWAY ANALYSIS

Name	p-value range	# Molecules
**Increased Levels of Alkaline Phosphatase**	6.98E-03 - 6.98E-03	1
**Increased Levels of Bilirubin**	2.08E-02 - 2.08E-02	1
**Increased Levels of AST**	2.63E-02 - 2.63E-02	1

Cardiotoxicity

Name	p-value range	# Molecules
**Cardiac Necrosis/Cell Death**	2.43E-01 - 1.40E-03	1
**Cardiac Enlargement**	2.18E-01 - 5.58E-03	2
**Cardiac Dysfunction**	4.25E-02 - 4.25E-02	1
**Cardiac Damage**	5.45E-02 - 5.45E-02	1
**Cardiac Arteriopathy**	6.16E-02 - 6.16E-02	2

Hepatotoxicity

Name	p-value range	# Molecules
**Liver Hyperplasia/Hyperproliferation**	1.55E-01 - 1.04E-03	17
**Dysfunction of liver**	6.98E-03 - 6.98E-03	1
**Hepatocellular carcinoma**	1.55E-01 - 1.53E-02	5
**Liver Proliferation**	4.38E-02 - 2.41E-02	2
**Glutathione Depletion In Liver**	2.63E-02 - 2.63E-02	1

Nephrotoxicity

(c) 2000-2021 QIAGEN. All rights reserved.

INGENUITY PATHWAY ANALYSIS

Name	p-value range	# Molecules
**Kidney Failure**	5.58E-03 - 5.58E-03	1
**Renal Necrosis/Cell Death**	3.23E-01 - 9.69E-02	2
**Renal Damage**	1.45E-01 - 1.22E-01	1
**Renal Tubule Injury**	1.22E-01 - 1.22E-01	1
**Renal Proliferation**	2.63E-01 - 2.63E-01	1

Top Regulator Effect Networks

Top Networks

ID	Associated Network Functions	Score
**1**	Cellular Movement, Hematological System Development and Function, Immune Cell Trafficking	23
**2**	Inflammatory Response, Drug Metabolism, Endocrine System Development and Function	21
**3**	Molecular Transport, Amino Acid Metabolism, Small Molecule Biochemistry	3

(c) 2000-2021 QIAGEN. All rights reserved.

**4**	Cancer, Hematological Disease, Immunological Disease	3
**5**	Cancer, Cell Death and Survival, Developmental Disorder	3

Top Tox Lists

Name	p-value	Overlap
**LPS/IL-1 Mediated Inhibition of RXR Function**	5.24E-03	1.2 % 3/253
**LXR/RXR Activation**	1.28E-02	1.6 % 2/123
**Persistent Renal Ischemia-Reperfusion Injury (Mouse)**	4.12E-02	3.3 % 1/30
**NF-B Signaling**	4.56E-02	0.5 % 3/574
**Hepatic Stellate Cell Activation**	4.78E-02	2.9 % 1/35

Top My Lists

Top My Pathways

Top Analysis-Ready Molecules

(c) 2000-2021 QIAGEN. All rights reserved.

INGENUITY PATHWAY ANALYSIS

Expr Log Ratio

Molecules	Expr. Value	Chart
TMEM233	↑ 3.021	
LYZ	↑ 1.962	
LMO7	↑ 0.698	
ENO3	↑ 0.606	
C1orf159	↑ 0.577	
CLK1	↑ 0.513	
RRNAD1	↑ 0.484	
LRRC45	↑ 0.459	
PRPF39	↑ 0.447	
LUC7L3	↑ 0.437	

Expr Log Ratio

Molecules	Expr. Value	Chart
IL1R2	↓ -1.106	
LARS2	↓ -0.822	
SMIM3	↓ -0.735	
ATF3	↓ -0.733	
TGFA	↓ -0.727	
CHST11	↓ -0.695	
PCDHB16	↓ -0.635	
B3GLCT	↓ -0.619	
MAN1A1	↓ -0.559	
LRRC8B	↓ -0.553	

(c) 2000-2021 QIAGEN. All rights reserved.

(c) 2000-2021 QIAGEN. All rights reserved.

INGENUITY
PATHWAY ANALYSIS
