# Supplementary material for: Reduction of Metastasis via Epigenetic Modulation in a Murine Model of Metastatic Triple Negative Breast Cancer (TNBC)
Source: Cancers (Basel). 2022 Mar 30;14(7):1753. doi: 10.3390/cancers14071753 (PMC8996906; doi:10.3390/cancers14071753)
Supplement: Supplementary file 1 [file cancers-14-01753-s001.zip › FileS6_ExperimentRINNumbers.pdf]

Assay Class: Eukaryote Total RNA Nano
Data Path: C:\...Eukaryote Total RNA Nano_DE72902546_2018-05-17_12-45-00.xad

Created: 5/17/2018 12:44:59 PM
Modified: 5/17/2018 1:08:50 PM

Electrophoresis File Run Summary

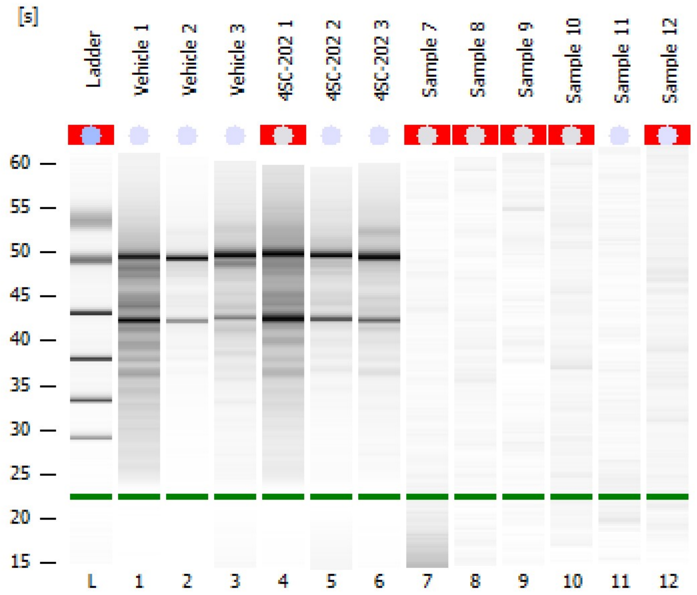

Instrument Information:

Instrument Name: DE72902546 Firmware: C.01.069

Serial#: DE72902546 Type: G2939A

Assay Information:

Assay Origin Path: C:\Program Files (x86)\Agilent\2100 bioanalyzer\2100 expert\assays\RNA\Eukaryote Total RNA Nano Series II.xsy

Assay Class: Eukaryote Total RNA Nano

Version: 2.6

Assay Comments: Total RNA Analysis ng sensitivity (Eukaryote)

© Copyright 2003 - 2009 Agilent Technologies, Inc.

Chip Information:

Chip Lot #:

Reagent Kit Lot #:

Chip Comments:

Vehicle 1

RIN: 4.30

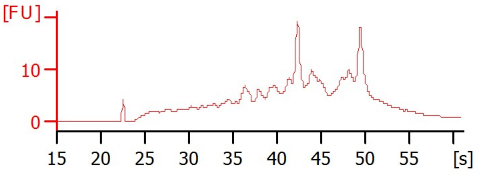

Vehicle 2

RIN: 8.80

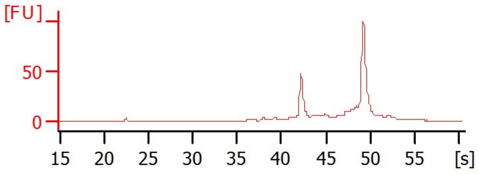

Vehicle 3

RIN: 5.70

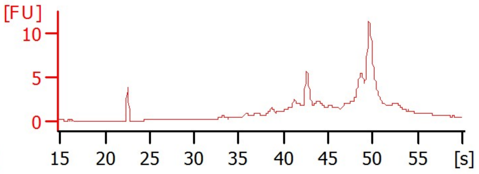

4SC-202 1

RIN N/A

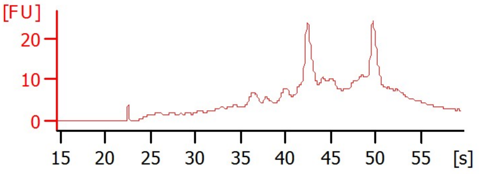

4SC-202 2

RIN: 8.90

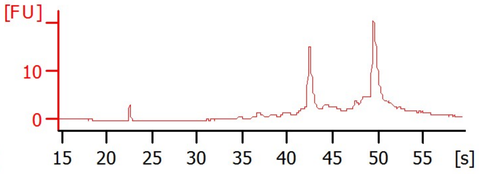

4SC-202 3

RIN:8

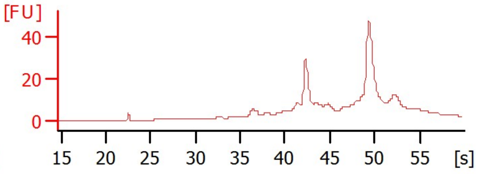

Sample 7

RIN N/A

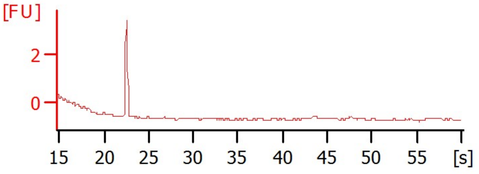

Sample 8

RIN N/A

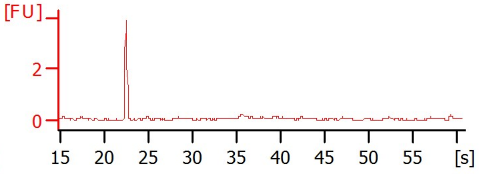

Sample 9

RIN N/A

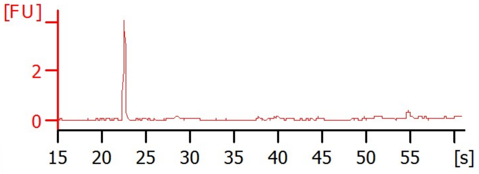

Sample 10

RIN N/A

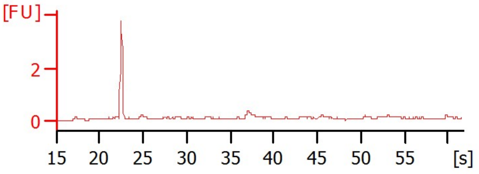

Sample 11

RIN:1

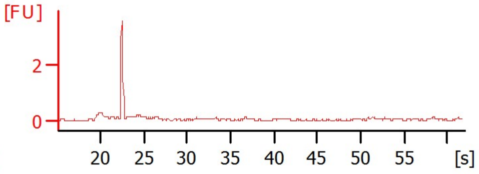

Sample 12

RIN:1

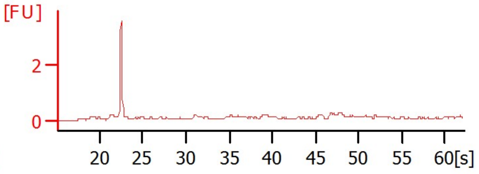

Assay Class: Eukaryote Total RNA Nano
Data Path: C:\...Eukaryote Total RNA Nano_DE72902546_2018-05-17_12-45-00.xad

Created: 5/17/2018 12:44:59 PM
Modified: 5/17/2018 1:08:50 PM

Electrophoresis File Run Summary (Chip Summary)

Sample Name	Sample Comment	Status	Result Label	Result Color
Vehicle 1		✓	RIN: 4.30	
Vehicle 2		✓	RIN: 8.80	
Vehicle 3		✓	RIN: 5.70	
4SC-202 1		✓	RIN N/A	
4SC-202 2		✓	RIN: 8.90	
4SC-202 3		✓	RIN:8	
Sample 7		✓	RIN N/A	
Sample 8		✓	RIN N/A	
Sample 9		✓	RIN N/A	
Sample 10		✗	RIN N/A	
Sample 11		✓	RIN:1	
Sample 12		✗	RIN:1	
Ladder		✗	All Other Samples	

Chip Lot # **Reagent Kit Lot #**

Chip Comments :

© Copyright 2003 - 2009 Agilent Technologies, Inc.

Assay Class: Eukaryote Total RNA Nano
Data Path: C:\...Eukaryote Total RNA Nano_DE72902546_2018-05-17_12-45-00.xad
Created: 5/17/2018 12:44:59 PM
Modified: 5/17/2018 1:08:50 PM

Electrophoresis Assay Details

General Analysis Settings

Number of Available Sample and Ladder Wells (Max.) : 13
Minimum Visible Range [s] : 17
Maximum Visible Range [s] : 70
Start Analysis Time Range [s] : 19
End Analysis Time Range [s] : 69
Ladder Concentration [ng/µl] : 150
Lower Marker Concentration [ng/µl] : 0
Upper Marker Concentration [ng/µl] : 0
Used Lower Marker for Quantitation
Standard Curve Fit is Logarithmic
Show Data Aligned to Lower Marker

Integrator Settings

Integration Start Time [s] : 19
Integration End Time [s] : 69
Slope Threshold : 0.6
Height Threshold [FU] : 0.5
Area Threshold : 0.2
Width Threshold [s] : 0.5
Baseline Plateau [s] : 6

Filter Settings

Filter Width [s] : 0.5
Polynomial Order : 4

Ladder

Ladder Peak	Size
1	25
2	200
3	500
4	1000
5	2000
6	4000

 © Copyright 2003 - 2009 Agilent Technologies, Inc.

Assay Class: Eukaryote Total RNA Nano
Data Path: C:\...Eukaryote Total RNA Nano_DE72902546_2018-05-17_12-45-00.xad
Created: 5/17/2018 12:44:59 PM
Modified: 5/17/2018 1:08:50 PM

Electropherogram Summary

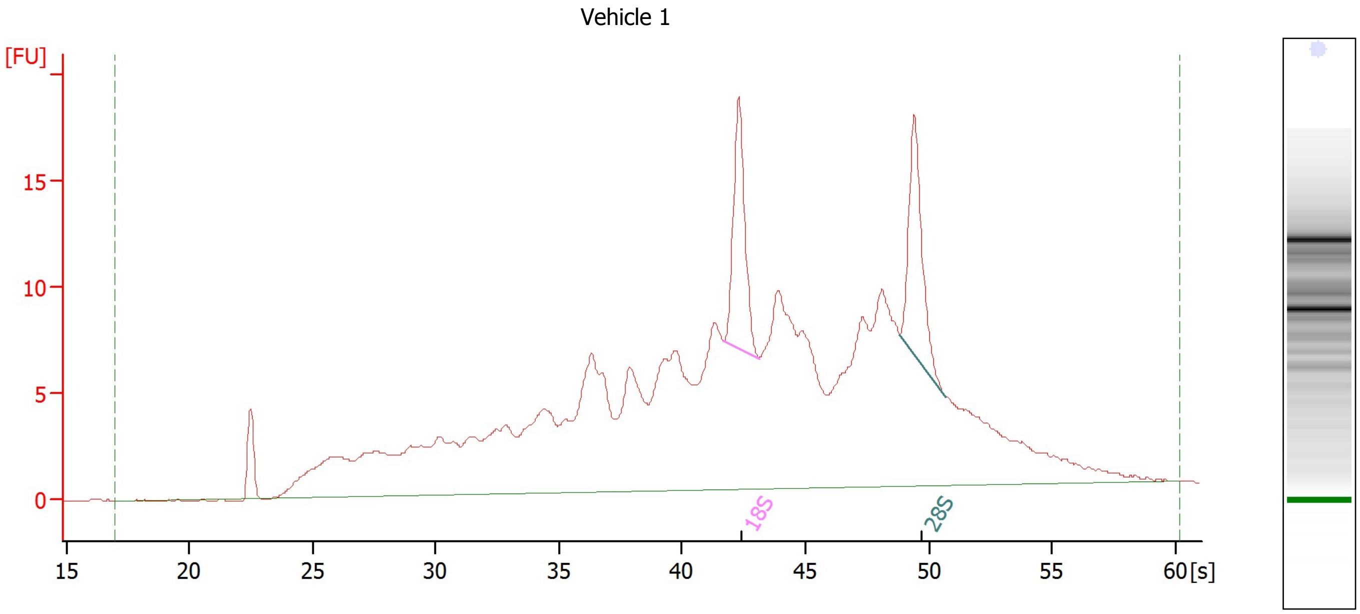

Overall Results for sample 1 : Vehicle 1

RNA Area:	358.2	RNA Integrity Number (RIN):	4.3 (B.02.08)
RNA Concentration:	881 ng/µl	Result Flagging Color:	
rRNA Ratio [28s / 18s]:	0.9	Result Flagging Label:	RIN: 4.30

Fragment table for sample 1 : Vehicle 1

Name	Start Time [s]	End Time [s]	Area	% of total Area
18S	41.69	43.13	14.8	4.1
28S	48.84	50.67	14.0	3.9

© Copyright 2003 - 2009 Agilent Technologies, Inc.

Assay Class: Eukaryote Total RNA Nano
Data Path: C:\...Eukaryote Total RNA Nano_DE72902546_2018-05-17_12-45-00.xad
Created: 5/17/2018 12:44:59 PM
Modified: 5/17/2018 1:08:50 PM

Electropherogram Summary Continued ...

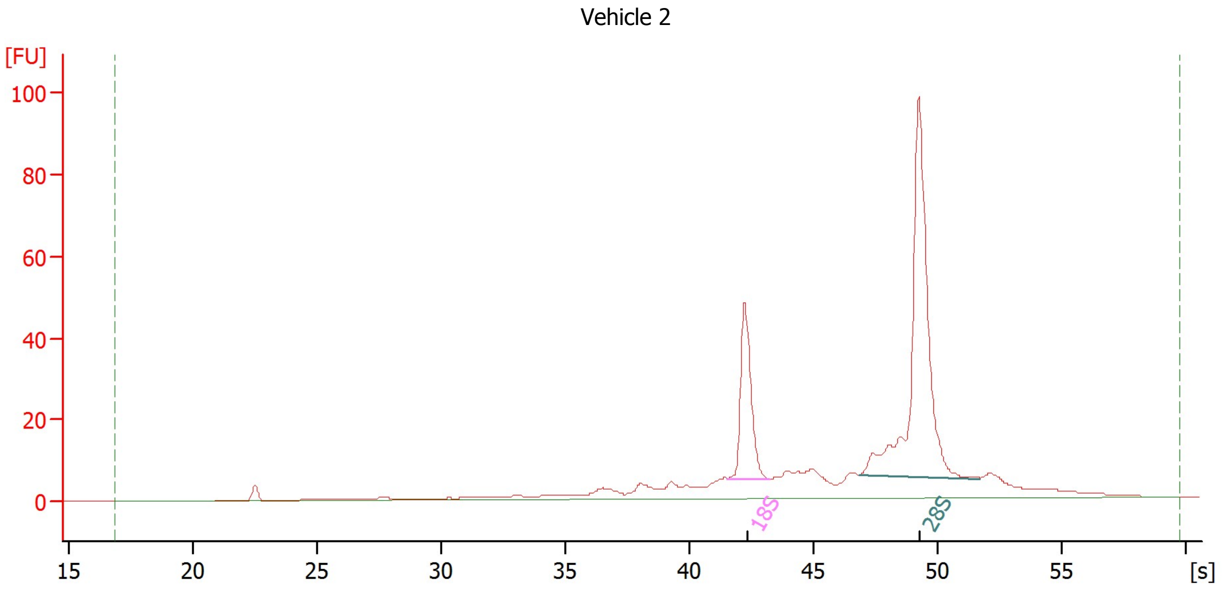

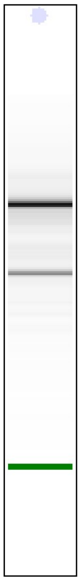

Overall Results for sample 2 : Vehicle 2

RNA Area:	389.5	RNA Integrity Number (RIN):	8.8 (B.02.08)
RNA Concentration:	958 ng/µl	Result Flagging Color:	
rRNA Ratio [28s / 18s]:	2.9	Result Flagging Label:	RIN: 8.80

Fragment table for sample 2 : Vehicle 2

Name	Start Time [s]	End Time [s]	Area	% of total Area
18S	41.54	43.23	48.6	12.5
28S	46.82	51.71	140.8	36.2

© Copyright 2003 - 2009 Agilent Technologies, Inc.

Assay Class: Eukaryote Total RNA Nano | Created: 5/17/2018 12:44:59 PM
Data Path: C:\...Eukaryote Total RNA Nano_DE72902546_2018-05-17_12-45-00.xad | Modified: 5/17/2018 1:08:50 PM

Electropherogram Summary Continued ...

Vehicle 3

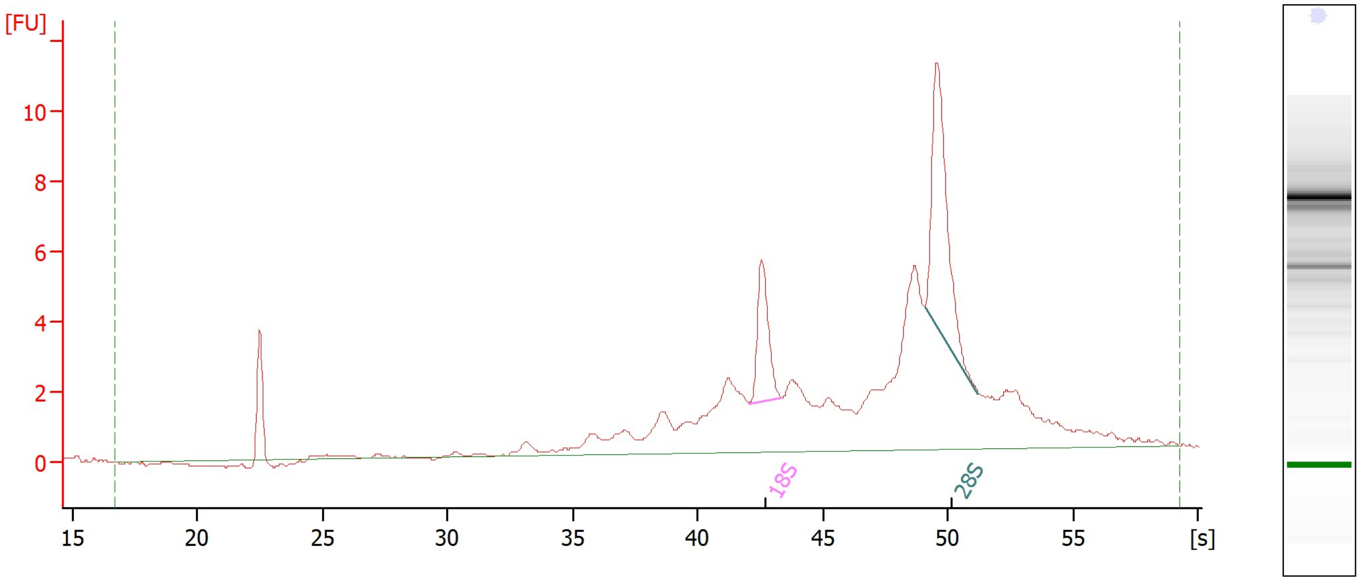

Overall Results for sample 3 : Vehicle 3

RNA Area:	85.1	RNA Integrity Number (RIN):	5.7 (B.02.08)
RNA Concentration:	209 ng/µl	Result Flagging Color:	
rRNA Ratio [28s / 18s]:	2.2	Result Flagging Label:	RIN: 5.70

Fragment table for sample 3 : Vehicle 3

Name	Start Time [s]	End Time [s]	Area	% of total Area
18S	42.04	43.37	4.7	5.5
28S	49.08	51.23	10.5	12.3

 © Copyright 2003 - 2009 Agilent Technologies, Inc.

Assay Class: Eukaryote Total RNA Nano
Data Path: C:\...Eukaryote Total RNA Nano_DE72902546_2018-05-17_12-45-00.xad

Created: 5/17/2018 12:44:59 PM
Modified: 5/17/2018 1:08:50 PM

Electropherogram Summary Continued ...

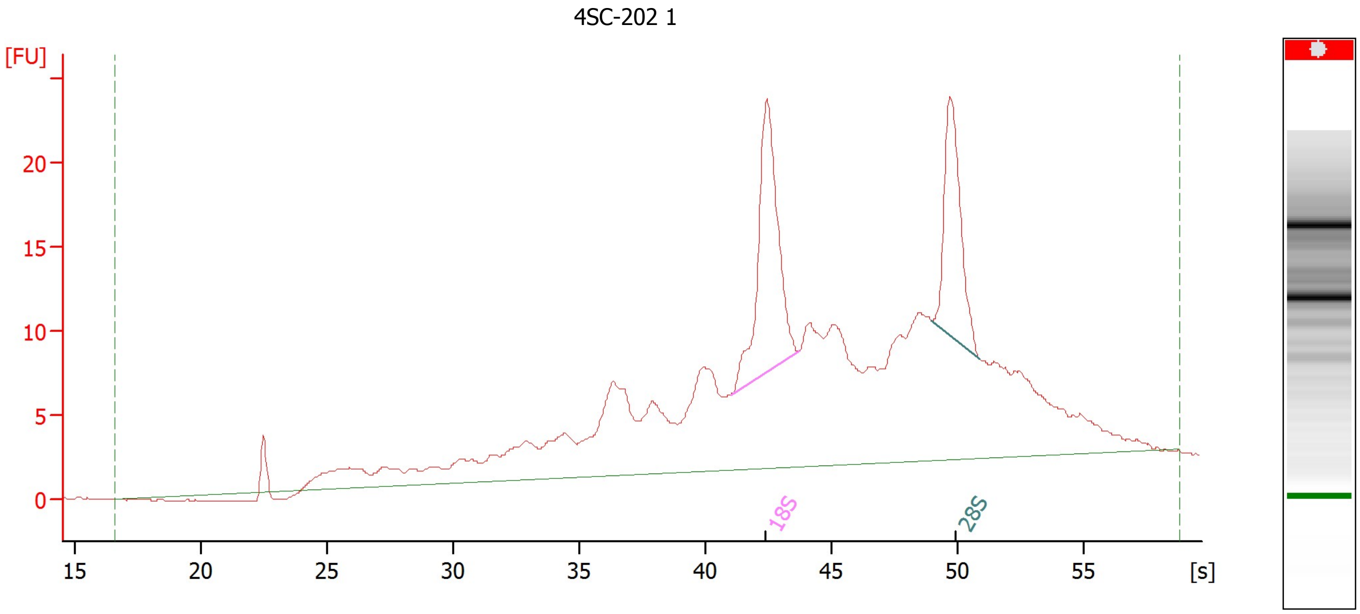

Overall Results for sample 4 : 4SC-202 1

RNA Area:	352.7	RNA Integrity Number (RIN):	N/A (B.02.08)
RNA Concentration:	867 ng/µl	Result Flagging Color:	
rRNA Ratio [28s / 18s]:	0.6	Result Flagging Label:	RIN N/A

Fragment table for sample 4 : 4SC-202 1

Name	Start Time [s]	End Time [s]	Area	% of total Area
18S	41.04	43.72	34.3	9.7
28S	49.01	50.88	21.4	6.1

 © Copyright 2003 - 2009 Agilent Technologies, Inc.

Assay Class: Eukaryote Total RNA Nano
Data Path: C:\...Eukaryote Total RNA Nano_DE72902546_2018-05-17_12-45-00.xad

Created: 5/17/2018 12:44:59 PM
Modified: 5/17/2018 1:08:50 PM

Electropherogram Summary Continued ...

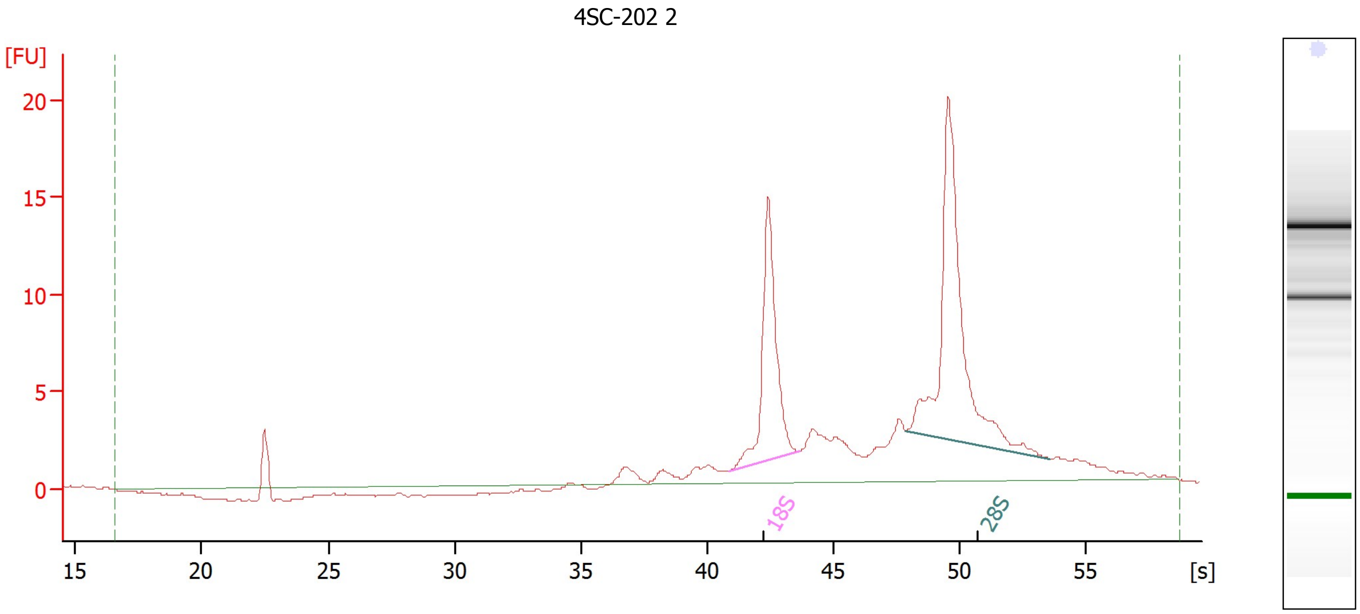

Overall Results for sample 5 : 4SC-202 2

RNA Area:	112.0	RNA Integrity Number (RIN):	8.9 (B.02.08)
RNA Concentration:	275 ng/µl	Result Flagging Color:	
rRNA Ratio [28s / 18s]:	1.7	Result Flagging Label:	RIN: 8.90

Fragment table for sample 5 : 4SC-202 2

Name	Start Time [s]	End Time [s]	Area	% of total Area
18S	40.92	43.64	19.7	17.6
28S	47.89	53.59	32.5	29.1

 © Copyright 2003 - 2009 Agilent Technologies, Inc.

Assay Class:	Eukaryote Total RNA Nano	Created:	5/17/2018 12:44:59 PM
Data Path:	C:\...Eukaryote Total RNA Nano_DE72902546_2018-05-17_12-45-00.xad	Modified:	5/17/2018 1:08:50 PM

Electropherogram Summary Continued ...

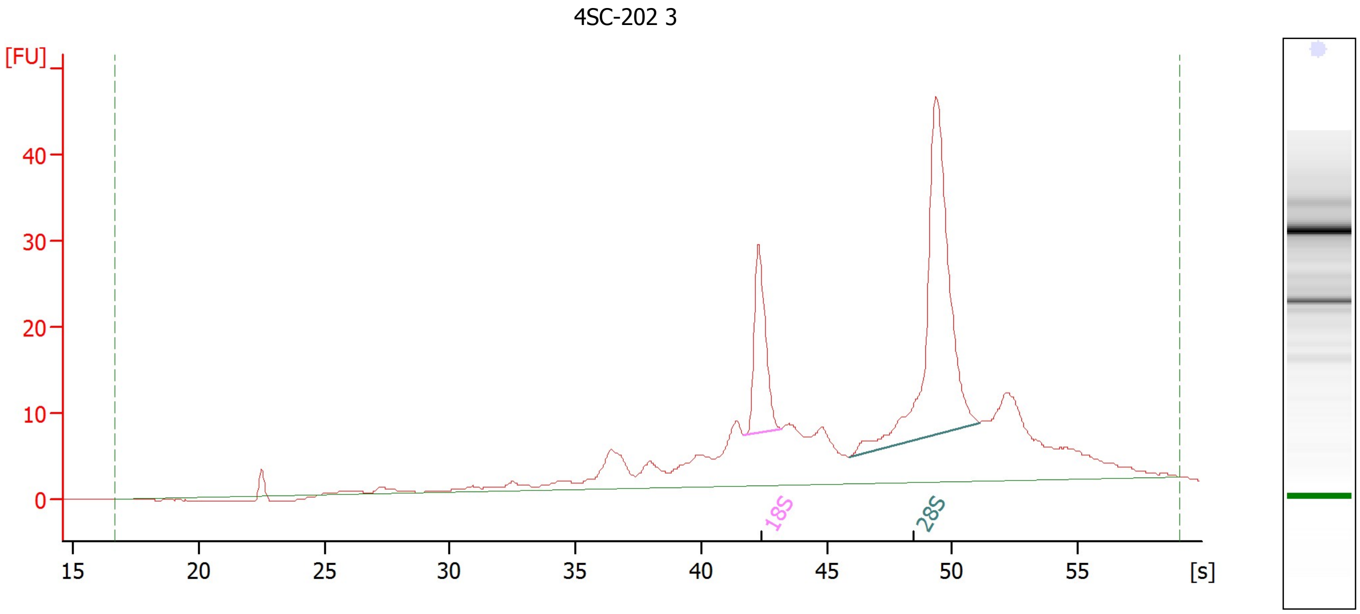

Overall Results for sample 6 : 4SC-202 3

RNA Area:	332.6	RNA Integrity Number (RIN):	8 (B.02.08)
RNA Concentration:	818 ng/µl	Result Flagging Color:	
rRNA Ratio [28s / 18s]:	3.0	Result Flagging Label:	RIN:8

Fragment table for sample 6 : 4SC-202 3

Name	Start Time [s]	End Time [s]	Area	% of total Area
18S	41.71	43.16	26.9	8.1
28S	45.90	51.07	80.9	24.3

 © Copyright 2003 - 2009 Agilent Technologies, Inc.

Assay Class: Eukaryote Total RNA Nano
Data Path: C:\...Eukaryote Total RNA Nano_DE72902546_2018-05-17_12-45-00.xad
Created: 5/17/2018 12:44:59 PM
Modified: 5/17/2018 1:08:50 PM

Electropherogram Summary Continued ...

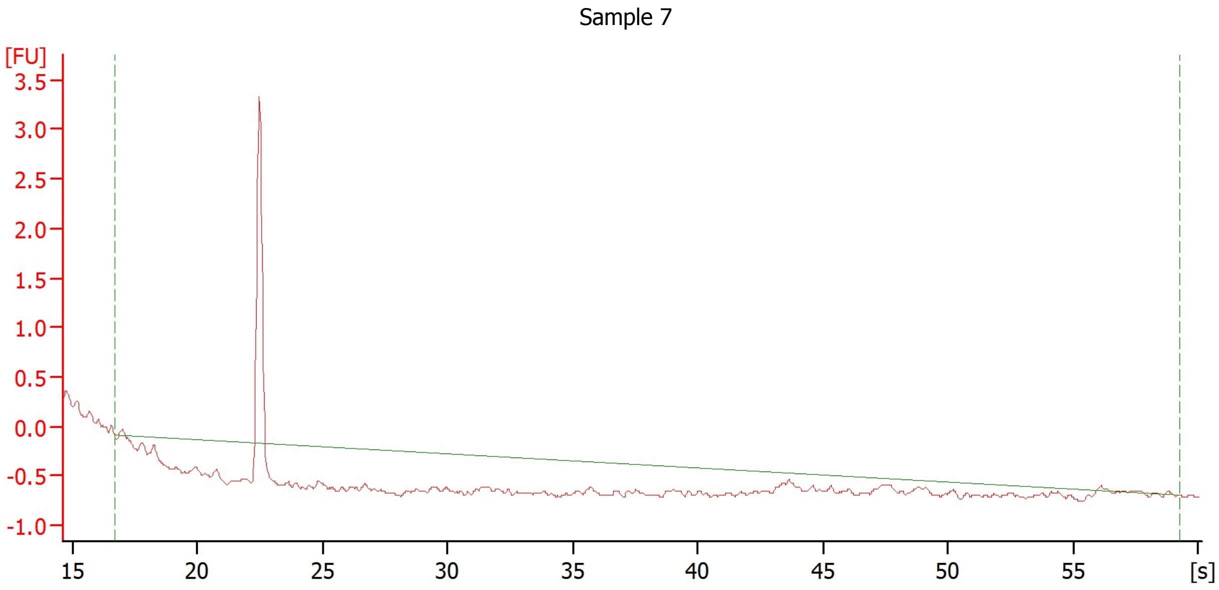

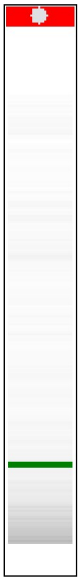

Overall Results for sample 7 : Sample 7

RNA Area:	0.1	RNA Integrity Number (RIN):	N/A (B.02.08)
RNA Concentration:	0 ng/µl	Result Flagging Color:	
rRNA Ratio [28s / 18s]:	0.0	Result Flagging Label:	RIN N/A

 © Copyright 2003 - 2009 Agilent Technologies, Inc.

Assay Class: Eukaryote Total RNA Nano
Data Path: C:\...Eukaryote Total RNA Nano_DE72902546_2018-05-17_12-45-00.xad
Created: 5/17/2018 12:44:59 PM
Modified: 5/17/2018 1:08:50 PM

Electropherogram Summary Continued ...

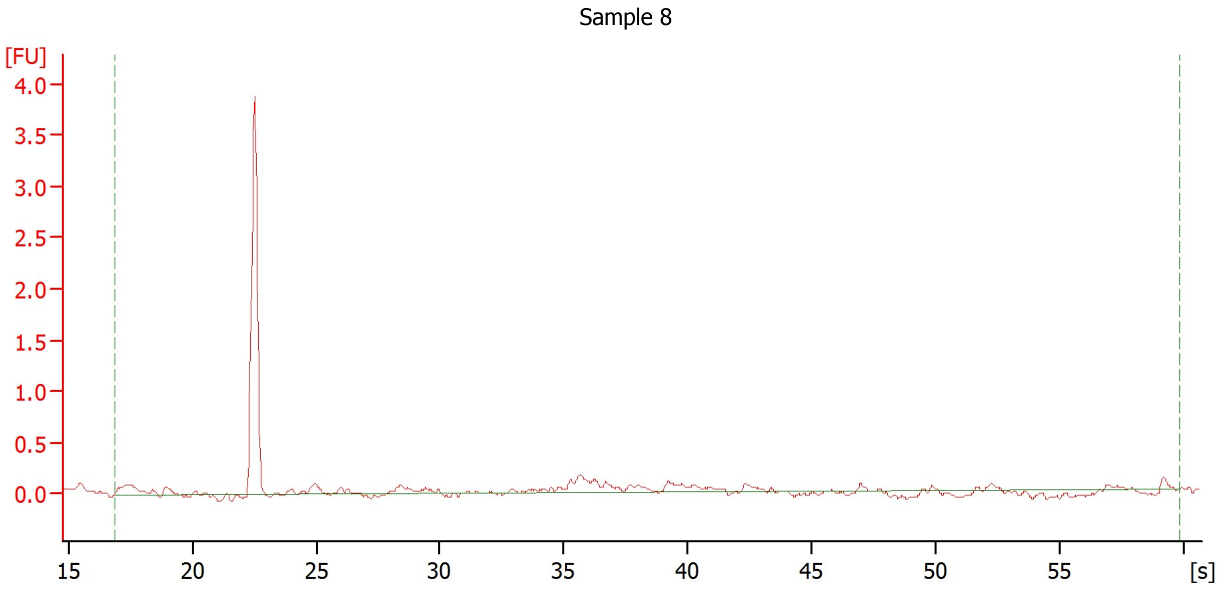

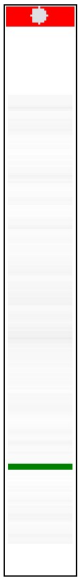

Overall Results for sample 8 : Sample 8

RNA Area:	2.6	RNA Integrity Number (RIN):	N/A (B.02.08)
RNA Concentration:	6 ng/µl	Result Flagging Color:	
rRNA Ratio [28s / 18s]:	0.0	Result Flagging Label:	RIN N/A

 © Copyright 2003 - 2009 Agilent Technologies, Inc.

Assay Class: Eukaryote Total RNA Nano
Data Path: C:\...Eukaryote Total RNA Nano_DE72902546_2018-05-17_12-45-00.xad
Created: 5/17/2018 12:44:59 PM
Modified: 5/17/2018 1:08:50 PM

Electropherogram Summary Continued ...

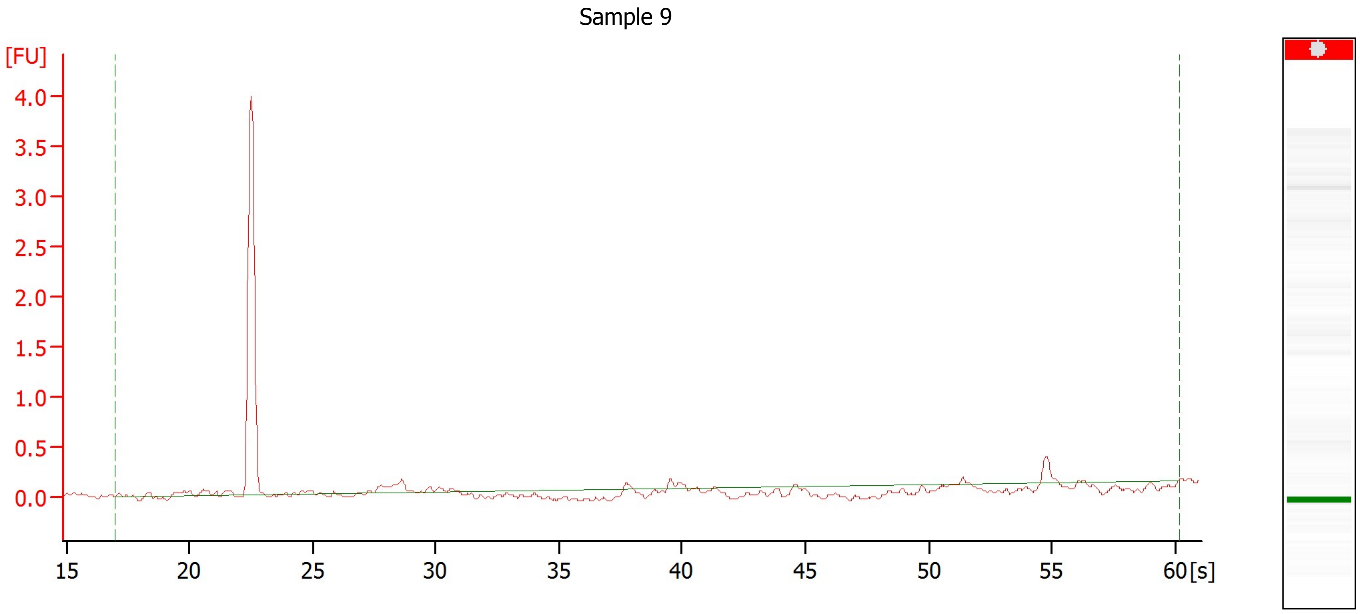

Overall Results for sample 9 : Sample 9

RNA Area:	1.4	RNA Integrity Number (RIN):	N/A (B.02.08)
RNA Concentration:	4 ng/µl	Result Flagging Color:	
rRNA Ratio [28s / 18s]:	0.0	Result Flagging Label:	RIN N/A

© Copyright 2003 - 2009 Agilent Technologies, Inc.

Assay Class: Eukaryote Total RNA Nano
Data Path: C:\...Eukaryote Total RNA Nano_DE72902546_2018-05-17_12-45-00.xad

Created: 5/17/2018 12:44:59 PM
Modified: 5/17/2018 1:08:50 PM

Electropherogram Summary Continued ...

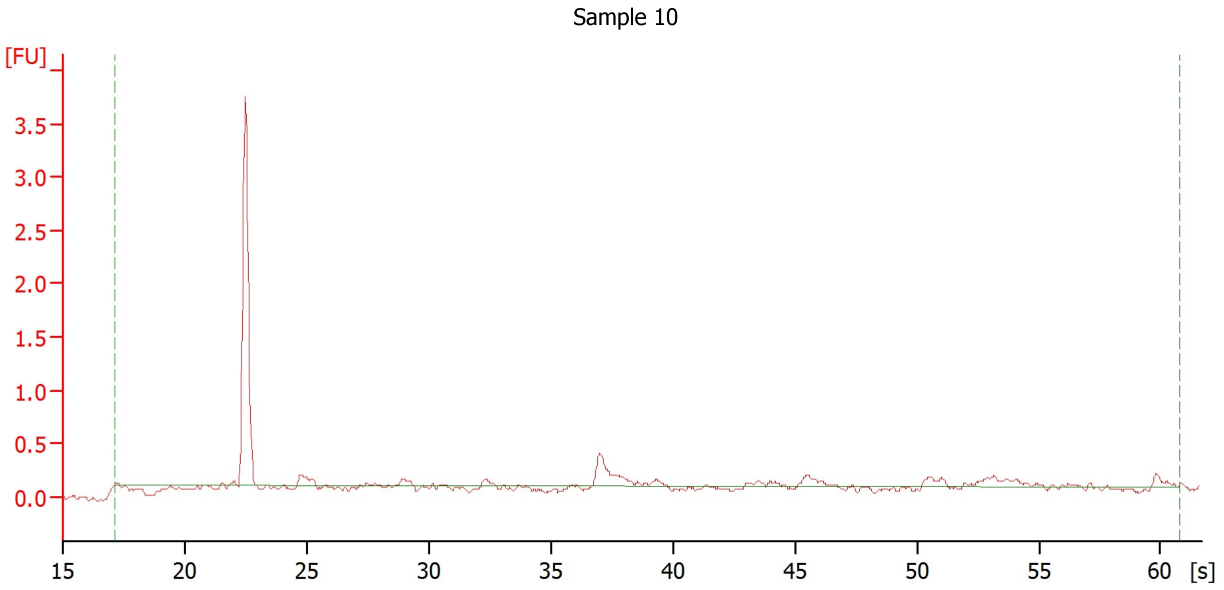

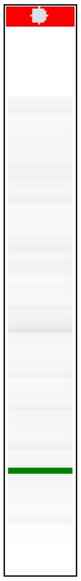

Overall Results for sample 10 : **Sample 10**

RNA Area:	1.8	RNA Integrity Number (RIN):	N/A (B.02.08)
RNA Concentration:	4 ng/µl	Result Flagging Color:	
rRNA Ratio [28s / 18s]:	0.0	Result Flagging Label:	RIN N/A

© Copyright 2003 - 2009 Agilent Technologies, Inc.

Assay Class: Eukaryote Total RNA Nano
Data Path: C:\...Eukaryote Total RNA Nano_DE72902546_2018-05-17_12-45-00.xad
Created: 5/17/2018 12:44:59 PM
Modified: 5/17/2018 1:08:50 PM

Electropherogram Summary Continued ...

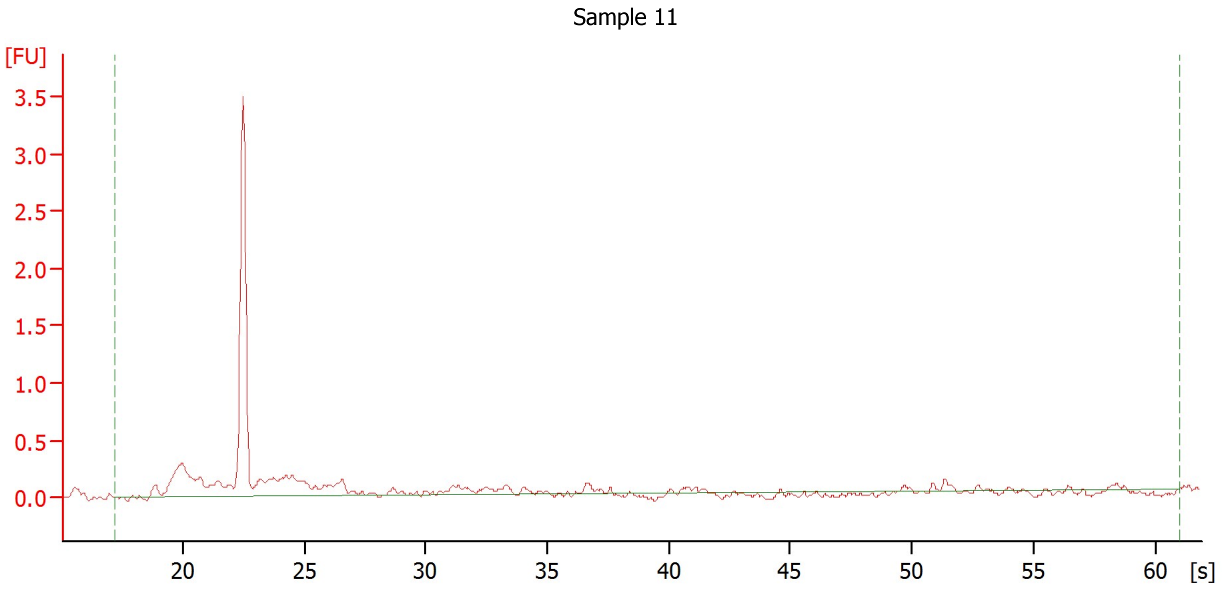

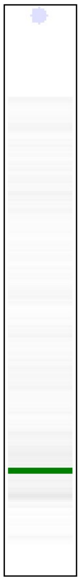

Overall Results for sample 11 : **Sample 11**

RNA Area:	5.4	RNA Integrity Number (RIN):	1 (B.02.08)
RNA Concentration:	13 ng/µl	Result Flagging Color:	
rRNA Ratio [28s / 18s]:	0.0	Result Flagging Label:	RIN:1

 © Copyright 2003 - 2009 Agilent Technologies, Inc.

Assay Class: Eukaryote Total RNA Nano
Data Path: C:\...Eukaryote Total RNA Nano_DE72902546_2018-05-17_12-45-00.xad
Created: 5/17/2018 12:44:59 PM
Modified: 5/17/2018 1:08:50 PM

Electropherogram Summary Continued ...

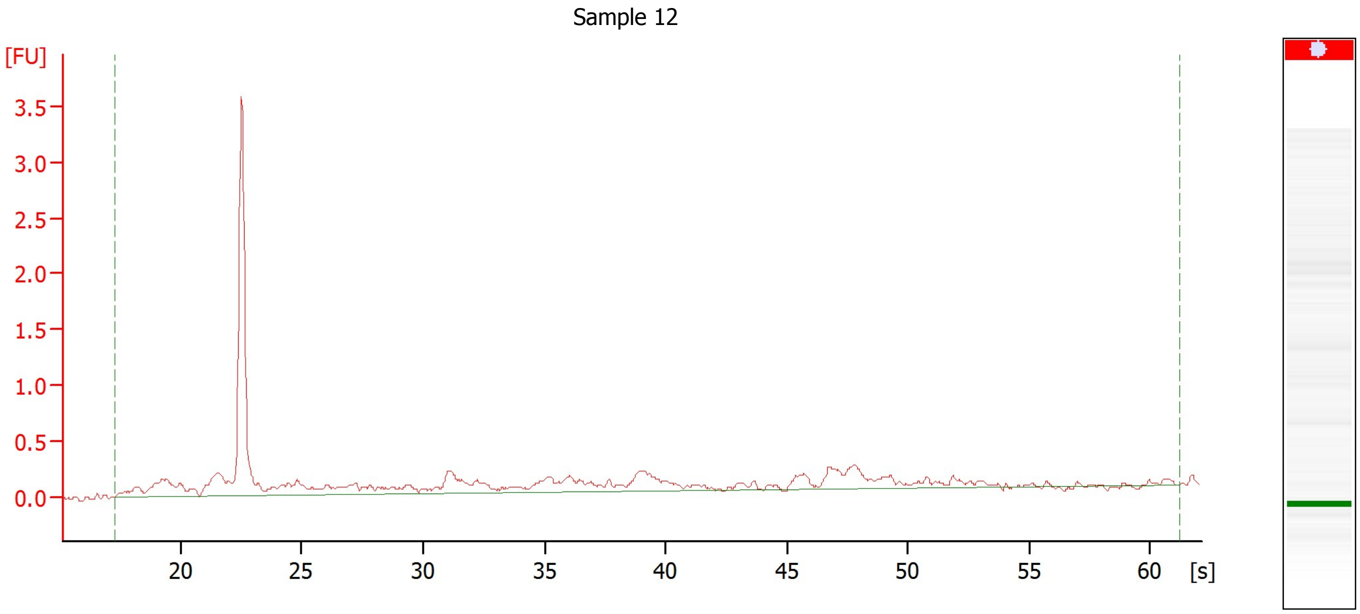

Overall Results for sample 12 : Sample 12

RNA Area:	8.4	RNA Integrity Number (RIN):	1 (B.02.08)
RNA Concentration:	21 ng/µl	Result Flagging Color:	
rRNA Ratio [28s / 18s]:	0.0	Result Flagging Label:	RIN:1

 © Copyright 2003 - 2009 Agilent Technologies, Inc.

Assay Class: Eukaryote Total RNA Nano
Data Path: C:\...Eukaryote Total RNA Nano_DE72902546_2019-11-07_15-38-37.xad

Created: 11/7/2019 3:38:37 PM
Modified: 11/7/2019 4:02:30 PM

Electrophoresis File Run Summary

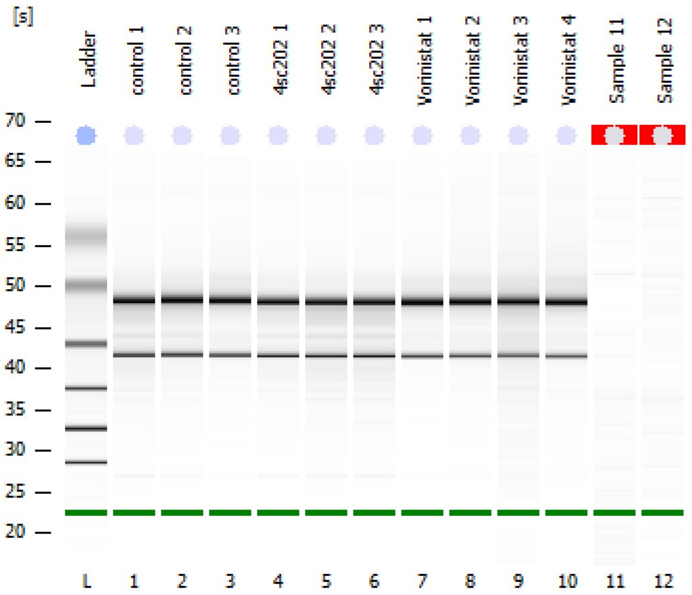

<u>Instrument Information:</u>

Instrument Name: DE72902546
Serial#: DE72902546
Firmware: C.01.069
Type: G2939A

<u>Assay Information:</u>

Assay Origin Path: C:\Program Files (x86)\Agilent\2100 bioanalyzer\2100 expert\assays\RNA\Eukaryote Total RNA Nano Series II.xsy

Assay Class: Eukaryote Total RNA Nano

Version: 2.6

Assay Comments: Total RNA Analysis ng sensitivity (Eukaryote)

© Copyright 2003 - 2009 Agilent Technologies, Inc.

<u>Chip Information:</u>

Chip Lot #:

Reagent Kit Lot #:

Chip Comments:

control 1

RIN: 9.40

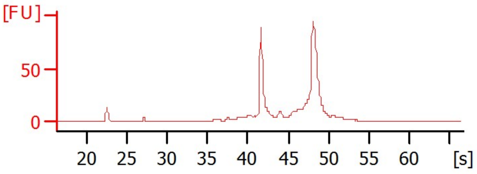

control 2

RIN: 9.90

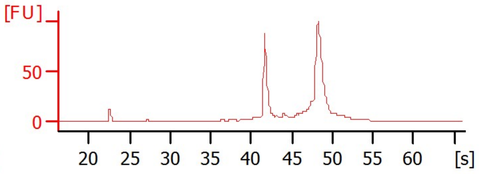

control 3

RIN:10

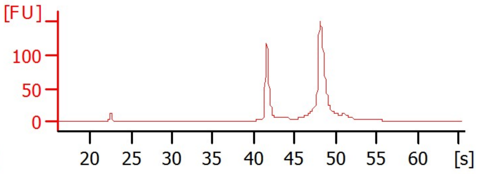

4sc202 1

RIN: 9.50

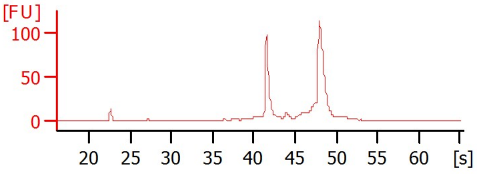

4sc202 2

RIN: 9.10

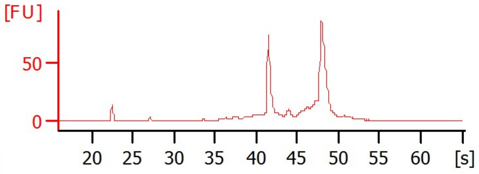

4sc202 3

RIN: 9.10

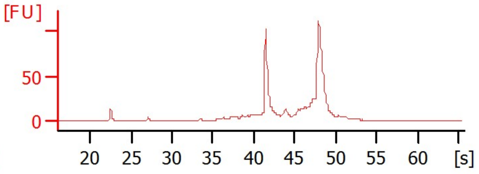

Vorinistat 1

RIN:10

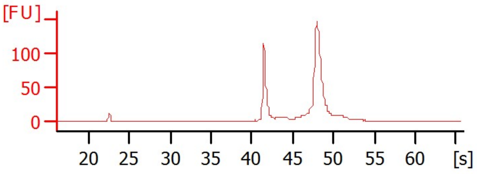

Vorinistat 2

RIN:10

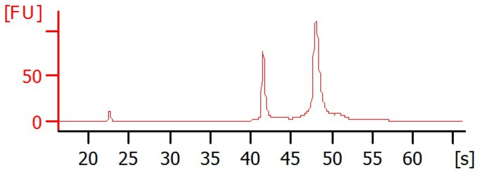

Vorinistat 3

RIN: 9.10

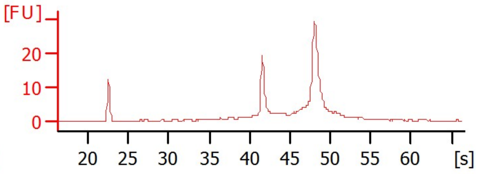

Vorinistat 4

RIN:10

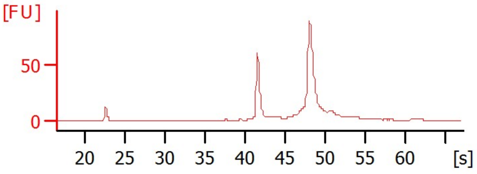

Sample 11

RIN N/A

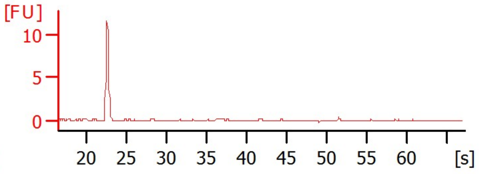

Sample 12

RIN N/A

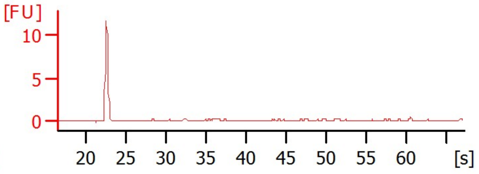

 © Copyright 2003 - 2009 Agilent Technologies, Inc.

Assay Class: Eukaryote Total RNA Nano
Data Path: C:\...Eukaryote Total RNA Nano_DE72902546_2019-11-07_15-38-37.xad
Created: 11/7/2019 3:38:37 PM
Modified: 11/7/2019 4:02:30 PM

Electrophoresis File Run Summary (Chip Summary)

Sample Name	Sample Comment	Status	Result Label	Result Color
control 1		✔	RIN: 9.40	
control 2		✔	RIN: 9.90	
control 3		✔	RIN:10	
4sc202 1		✔	RIN: 9.50	
4sc202 2		✔	RIN: 9.10	
4sc202 3		✔	RIN: 9.10	
Vorinistat 1		✔	RIN:10	
Vorinistat 2		✔	RIN:10	
Vorinistat 3		✔	RIN: 9.10	
Vorinistat 4		✔	RIN:10	
Sample 11		✔	RIN N/A	
Sample 12		✔	RIN N/A	
Ladder		✔	All Other Samples	

Chip Lot # **Reagent Kit Lot #**

Chip Comments :

© Copyright 2003 - 2009 Agilent Technologies, Inc.

Assay Class:	Eukaryote Total RNA Nano	Created:	11/7/2019 3:38:37 PM
Data Path:	C:\...Eukaryote Total RNA Nano_DE72902546_2019-11-07_15-38-37.xad	Modified:	11/7/2019 4:02:30 PM

Electrophoresis Assay Details

General Analysis Settings

Number of Available Sample and Ladder Wells (Max.) : 13
Minimum Visible Range [s] : 17
Maximum Visible Range [s] : 70
Start Analysis Time Range [s] : 19
End Analysis Time Range [s] : 69
Ladder Concentration [ng/µl] : 150
Lower Marker Concentration [ng/µl] : 0
Upper Marker Concentration [ng/µl] : 0
Used Lower Marker for Quantitation
Standard Curve Fit is Logarithmic
Show Data Aligned to Lower Marker

Integrator Settings

Integration Start Time [s] : 19
Integration End Time [s] : 69
Slope Threshold : 0.6
Height Threshold [FU] : 0.5
Area Threshold : 0.2
Width Threshold [s] : 0.5
Baseline Plateau [s] : 6

Filter Settings

Filter Width [s] : 0.5
Polynomial Order : 4

Ladder

Ladder Peak	Size
1	25
2	200
3	500
4	1000
5	2000
6	4000

 © Copyright 2003 - 2009 Agilent Technologies, Inc.

Assay Class:	Eukaryote Total RNA Nano	Created:	11/7/2019 3:38:37 PM
Data Path:	C:\...Eukaryote Total RNA Nano_DE72902546_2019-11-07_15-38-37.xad	Modified:	11/7/2019 4:02:30 PM

Electropherogram Summary

control 1

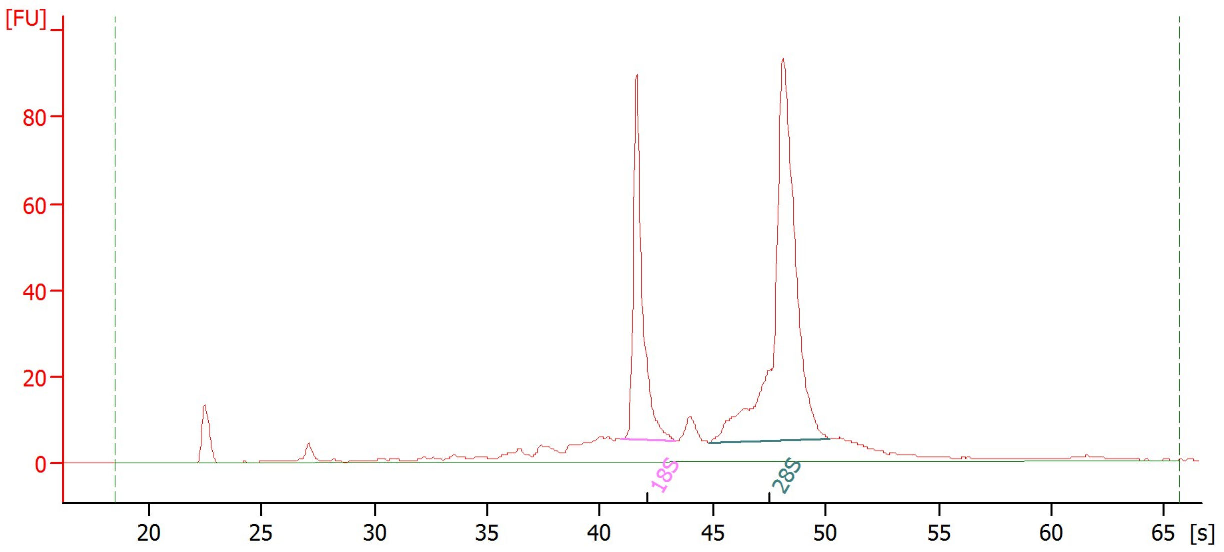

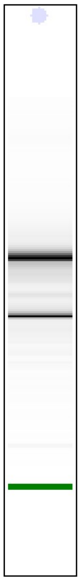

Overall Results for sample 1 : control 1

RNA Area:	505.2	RNA Integrity Number (RIN):	9.4 (B.02.08)
RNA Concentration:	391 ng/µl	Result Flagging Color:	
rRNA Ratio [28s / 18s]:	2.2	Result Flagging Label:	RIN: 9.40

Fragment table for sample 1 : control 1

Name	Start Time [s]	End Time [s]	Area	% of total Area
18S	40.96	43.34	94.7	18.7
28S	44.86	50.18	204.6	40.5

 © Copyright 2003 - 2009 Agilent Technologies, Inc.

Assay Class:	Eukaryote Total RNA Nano	Created:	11/7/2019 3:38:37 PM
Data Path:	C:\...Eukaryote Total RNA Nano_DE72902546_2019-11-07_15-38-37.xad	Modified:	11/7/2019 4:02:30 PM

Electropherogram Summary Continued ...

control 2

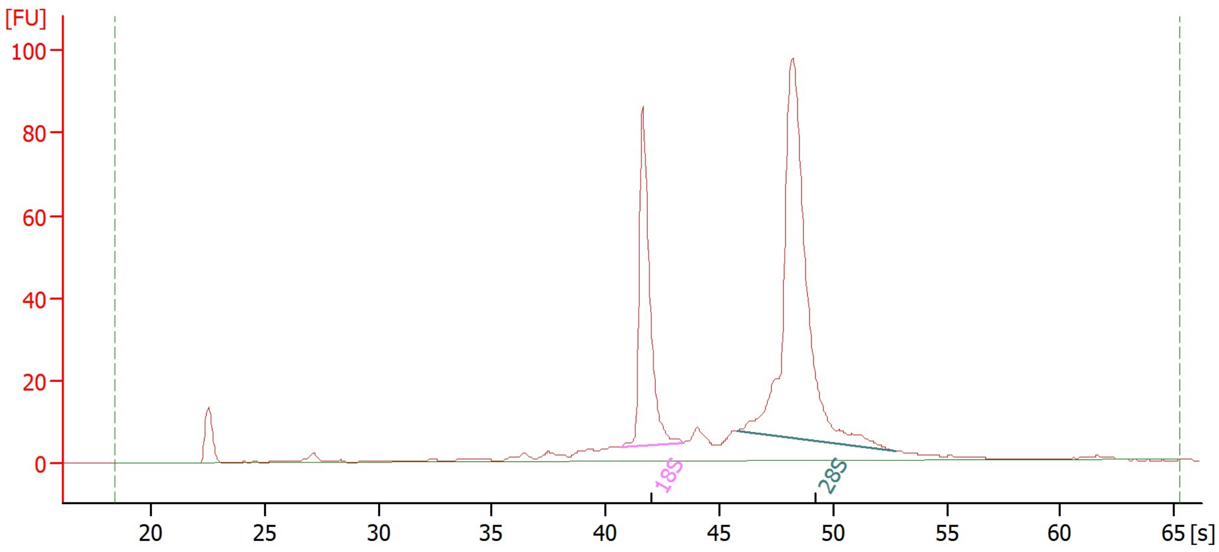

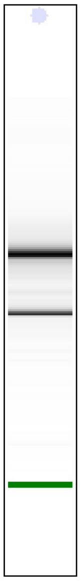

Overall Results for sample 2 : control 2

RNA Area:	505.2	RNA Integrity Number (RIN):	9.9 (B.02.08)
RNA Concentration:	391 ng/µl	Result Flagging Color:	
rRNA Ratio [28s / 18s]:	2.2	Result Flagging Label:	RIN: 9.90

Fragment table for sample 2 : control 2

Name	Start Time [s]	End Time [s]	Area	% of total Area
18S	40.65	43.39	101.8	20.1
28S	45.76	52.75	222.1	44.0

 © Copyright 2003 - 2009 Agilent Technologies, Inc.

Assay Class:	Eukaryote Total RNA Nano	Created:	11/7/2019 3:38:37 PM
Data Path:	C:\...Eukaryote Total RNA Nano_DE72902546_2019-11-07_15-38-37.xad	Modified:	11/7/2019 4:02:30 PM

Electropherogram Summary Continued ...

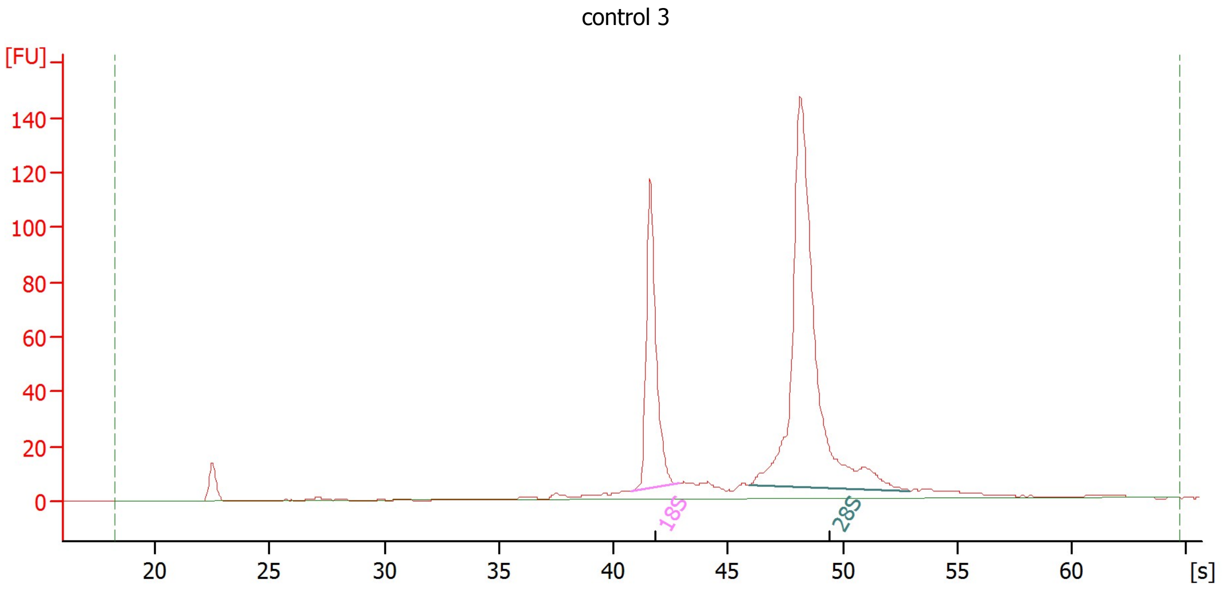

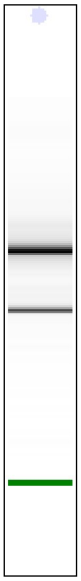

Overall Results for sample 3 : control 3

RNA Area:	634.5	RNA Integrity Number (RIN):	10 (B.02.08)
RNA Concentration:	492 ng/µl	Result Flagging Color:	
rRNA Ratio [28s / 18s]:	2.5	Result Flagging Label:	RIN:10

Fragment table for sample 3 : control 3

Name	Start Time [s]	End Time [s]	Area	% of total Area
18S	40.78	42.84	133.4	21.0
28S	45.94	52.92	333.9	52.6

 © Copyright 2003 - 2009 Agilent Technologies, Inc.

Assay Class: Eukaryote Total RNA Nano
Data Path: C:\...Eukaryote Total RNA Nano_DE72902546_2019-11-07_15-38-37.xad
Created: 11/7/2019 3:38:37 PM
Modified: 11/7/2019 4:02:30 PM

Electropherogram Summary Continued ...

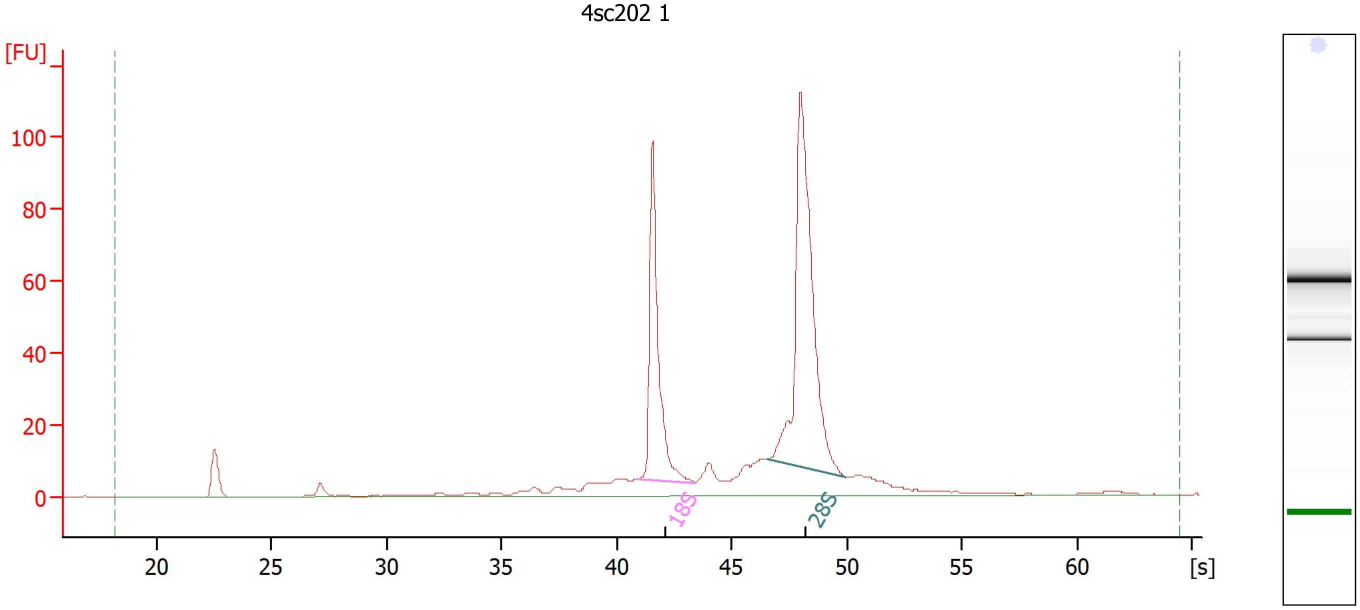

Overall Results for sample 4 : 4sc202 1

RNA Area:	495.2	RNA Integrity Number (RIN):	9.5 (B.02.08)
RNA Concentration:	384 ng/µl	Result Flagging Color:	
rRNA Ratio [28s / 18s]:	1.8	Result Flagging Label:	RIN: 9.50

Fragment table for sample 4 : 4sc202 1

Name	Start Time [s]	End Time [s]	Area	% of total Area
18S	40.89	43.37	101.2	20.4
28S	46.49	49.90	184.6	37.3

 © Copyright 2003 - 2009 Agilent Technologies, Inc.

Assay Class:	Eukaryote Total RNA Nano	Created:	11/7/2019 3:38:37 PM
Data Path:	C:\...Eukaryote Total RNA Nano_DE72902546_2019-11-07_15-38-37.xad	Modified:	11/7/2019 4:02:30 PM

Electropherogram Summary Continued ...

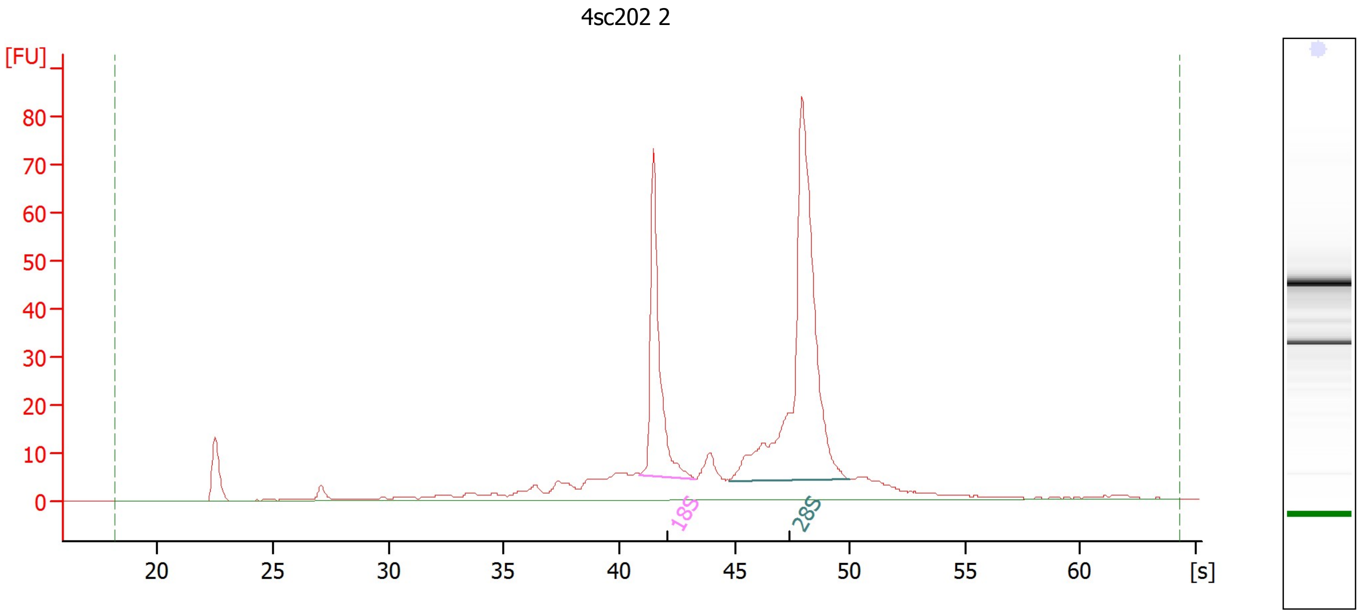

Overall Results for sample 5 : 4sc202 2

RNA Area:	445.4	RNA Integrity Number (RIN):	9.1 (B.02.08)
RNA Concentration:	345 ng/µl	Result Flagging Color:	
rRNA Ratio [28s / 18s]:	2.4	Result Flagging Label:	RIN: 9.10

Fragment table for sample 5 : 4sc202 2

Name	Start Time [s]	End Time [s]	Area	% of total Area
18S	40.90	43.28	73.4	16.5
28S	44.77	49.98	173.0	38.8

Assay Class: Eukaryote Total RNA Nano
Data Path: C:\...Eukaryote Total RNA Nano_DE72902546_2019-11-07_15-38-37.xad
Created: 11/7/2019 3:38:37 PM
Modified: 11/7/2019 4:02:30 PM

Electropherogram Summary Continued ...

4sc202 3

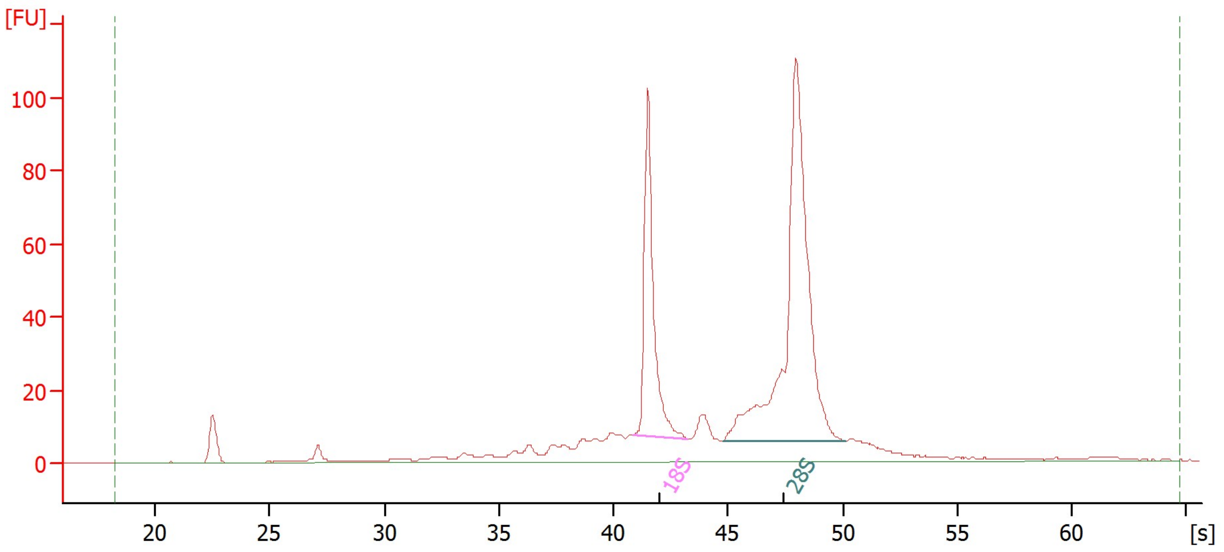

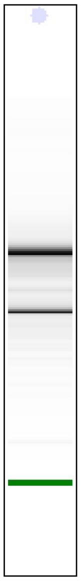

Overall Results for sample 6 : 4sc202 3

RNA Area:	609.4	RNA Integrity Number (RIN):	9.1 (B.02.08)
RNA Concentration:	472 ng/µl	Result Flagging Color:	
rRNA Ratio [28s / 18s]:	2.3	Result Flagging Label:	RIN: 9.10

Fragment table for sample 6 : 4sc202 3

Name	Start Time [s]	End Time [s]	Area	% of total Area
18S	40.88	43.27	101.8	16.7
28S	44.77	50.16	235.9	38.7

 © Copyright 2003 - 2009 Agilent Technologies, Inc.

Assay Class: Eukaryote Total RNA Nano
Data Path: C:\...Eukaryote Total RNA Nano_DE72902546_2019-11-07_15-38-37.xad

Created: 11/7/2019 3:38:37 PM
Modified: 11/7/2019 4:02:30 PM

Electropherogram Summary Continued ...

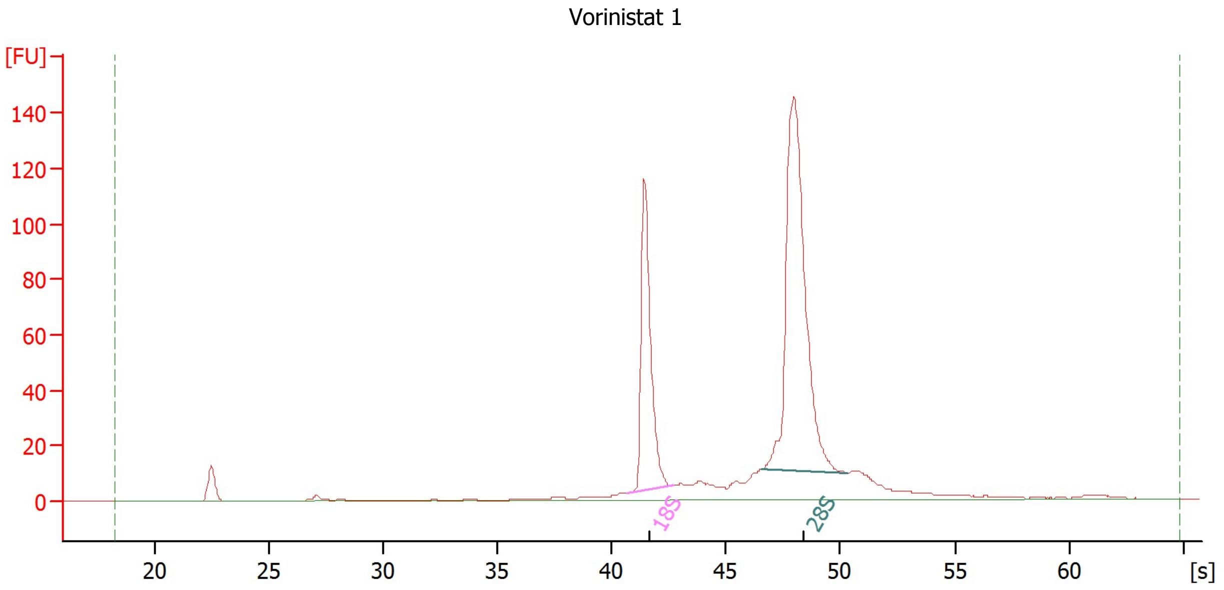

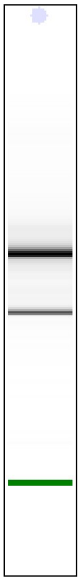

Overall Results for sample 7 : Vorinistat 1

RNA Area:	611.4	RNA Integrity Number (RIN):	10 (B.02.08)
RNA Concentration:	474 ng/µl	Result Flagging Color:	
rRNA Ratio [28s / 18s]:	2.1	Result Flagging Label:	RIN:10

Fragment table for sample 7 : Vorinistat 1

Name	Start Time [s]	End Time [s]	Area	% of total Area
18S	40.73	42.75	126.1	20.6
28S	46.60	50.31	269.6	44.1

 © Copyright 2003 - 2009 Agilent Technologies, Inc.

Assay Class: Eukaryote Total RNA Nano
Data Path: C:\...Eukaryote Total RNA Nano_DE72902546_2019-11-07_15-38-37.xad
Created: 11/7/2019 3:38:37 PM
Modified: 11/7/2019 4:02:30 PM

Electropherogram Summary Continued ...

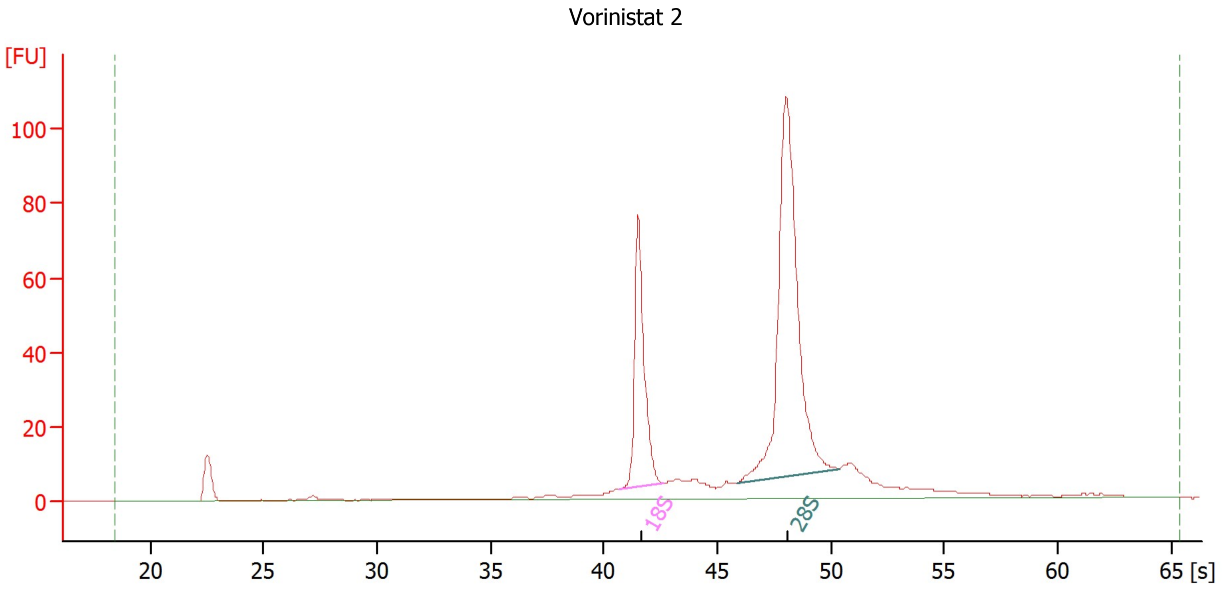

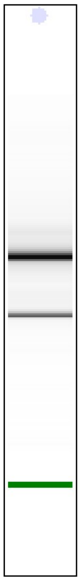

Overall Results for sample 8 : Vorinistat 2

RNA Area:	465.5	RNA Integrity Number (RIN):	10 (B.02.08)
RNA Concentration:	361 ng/µl	Result Flagging Color:	
rRNA Ratio [28s / 18s]:	2.4	Result Flagging Label:	RIN:10

Fragment table for sample 8 : Vorinistat 2

Name	Start Time [s]	End Time [s]	Area	% of total Area
18S	40.69	42.58	85.4	18.3
28S	45.85	50.35	206.7	44.4

© Copyright 2003 - 2009 Agilent Technologies, Inc.

Assay Class:	Eukaryote Total RNA Nano	Created:	11/7/2019 3:38:37 PM
Data Path:	C:\...Eukaryote Total RNA Nano_DE72902546_2019-11-07_15-38-37.xad	Modified:	11/7/2019 4:02:30 PM

Electropherogram Summary Continued ...

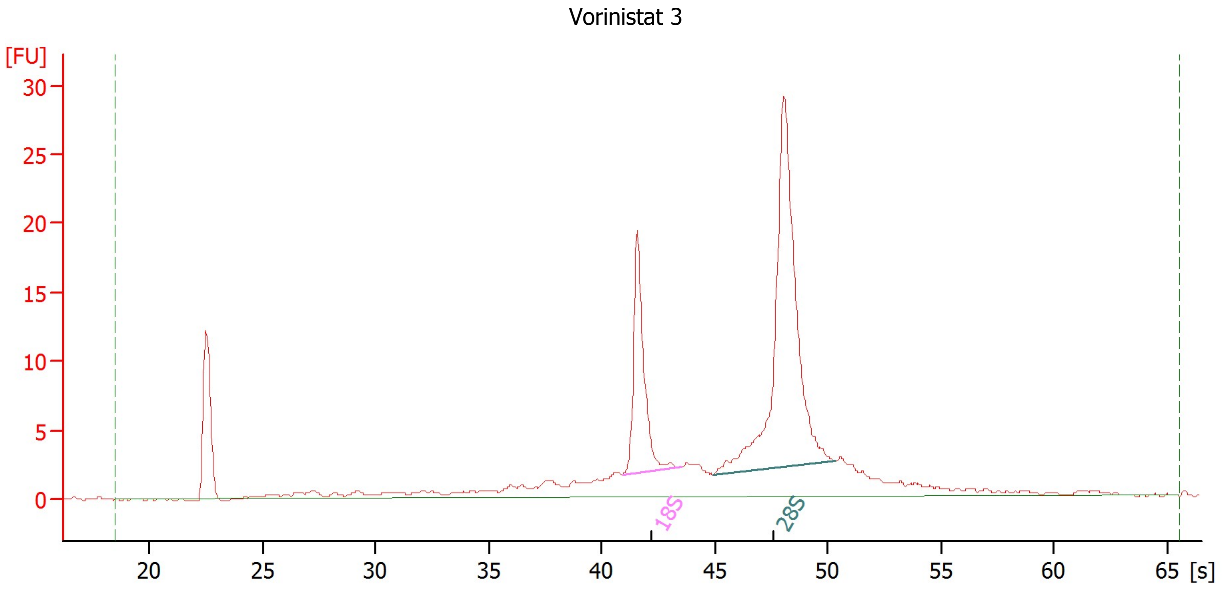

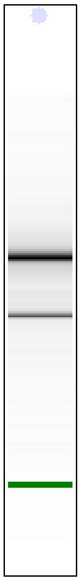

Overall Results for sample 9 : Vorinistat 3

RNA Area:	170.8	RNA Integrity Number (RIN):	9.1 (B.02.08)
RNA Concentration:	132 ng/µl	Result Flagging Color:	
rRNA Ratio [28s / 18s]:	2.7	Result Flagging Label:	RIN: 9.10

Fragment table for sample 9 : Vorinistat 3

Name	Start Time [s]	End Time [s]	Area	% of total Area
18S	40.92	43.53	22.1	12.9
28S	44.91	50.32	59.7	34.9

 © Copyright 2003 - 2009 Agilent Technologies, Inc.

Assay Class:	Eukaryote Total RNA Nano	Created:	11/7/2019 3:38:37 PM
Data Path:	C:\...Eukaryote Total RNA Nano_DE72902546_2019-11-07_15-38-37.xad	Modified:	11/7/2019 4:02:30 PM

Electropherogram Summary Continued ...

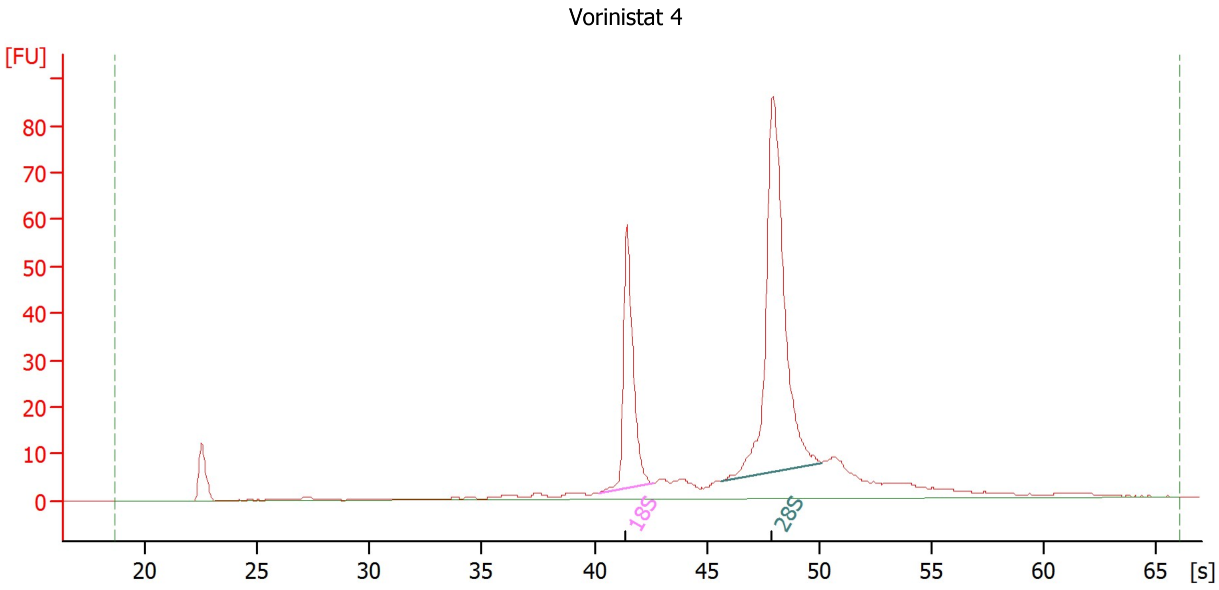

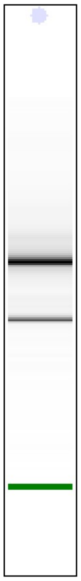

Overall Results for sample 10 : Vorinistat 4

RNA Area:	379.0	RNA Integrity Number (RIN):	10 (B.02.08)
RNA Concentration:	294 ng/µl	Result Flagging Color:	
rRNA Ratio [28s / 18s]:	2.3	Result Flagging Label:	RIN:10

Fragment table for sample 10 : Vorinistat 4

Name	Start Time [s]	End Time [s]	Area	% of total Area
18S	40.21	42.61	66.9	17.7
28S	45.67	50.12	156.7	41.3

 © Copyright 2003 - 2009 Agilent Technologies, Inc.

Assay Class: Eukaryote Total RNA Nano
Data Path: C:\...Eukaryote Total RNA Nano_DE72902546_2019-11-07_15-38-37.xad
Created: 11/7/2019 3:38:37 PM
Modified: 11/7/2019 4:02:30 PM

Electropherogram Summary Continued ...

Sample 11

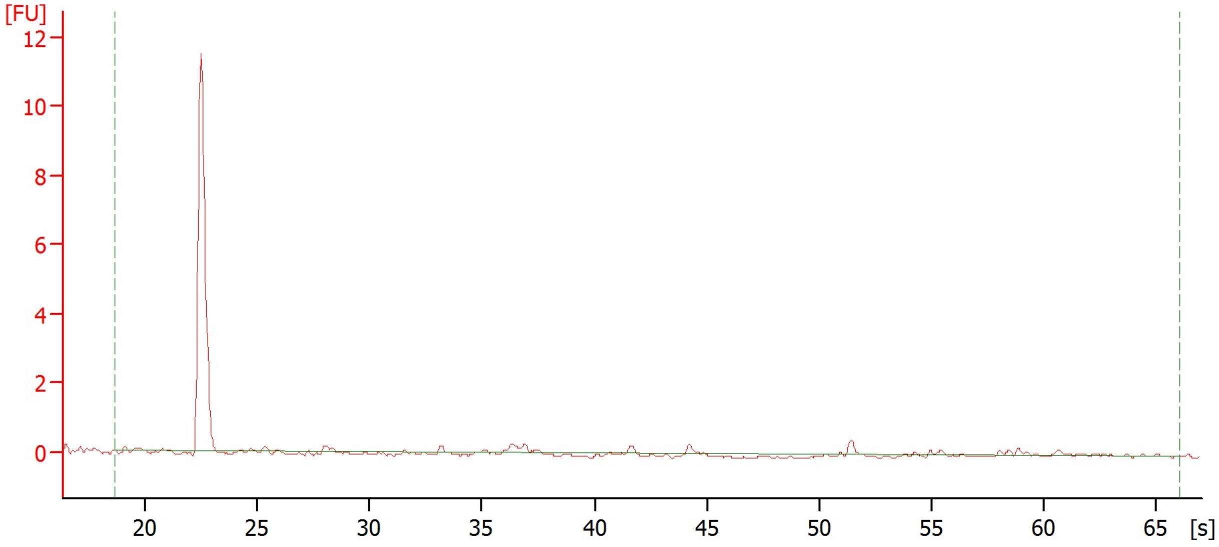

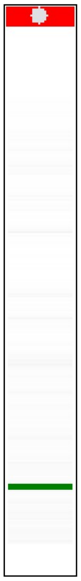

Overall Results for sample 11 : **Sample 11**

RNA Area:	2.4	RNA Integrity Number (RIN):	N/A (B.02.08)
RNA Concentration:	2 ng/µl	Result Flagging Color:	
rRNA Ratio [28s / 18s]:	0.0	Result Flagging Label:	RIN N/A

© Copyright 2003 - 2009 Agilent Technologies, Inc.

Assay Class: Eukaryote Total RNA Nano
Data Path: C:\...Eukaryote Total RNA Nano_DE72902546_2019-11-07_15-38-37.xad
Created: 11/7/2019 3:38:37 PM
Modified: 11/7/2019 4:02:30 PM

Electropherogram Summary Continued ...

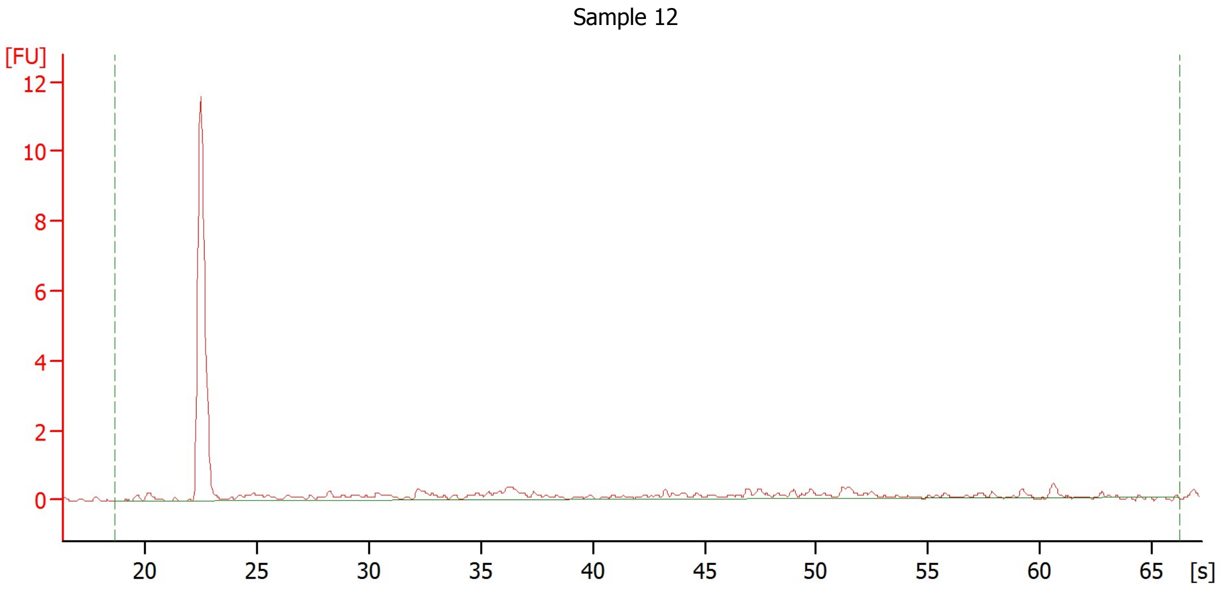

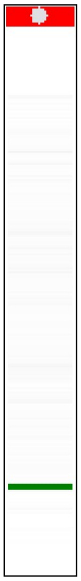

Overall Results for sample 12 : **Sample 12**

RNA Area:	10.7	RNA Integrity Number (RIN):	N/A (B.02.08)
RNA Concentration:	8 ng/µl	Result Flagging Color:	
rRNA Ratio [28s / 18s]:	0.0	Result Flagging Label:	RIN N/A

 © Copyright 2003 - 2009 Agilent Technologies, Inc.

Assay Class: Eukaryote Total RNA Nano
Data Path: C:\...Eukaryote Total RNA Nano_DE72902546_2019-11-07_15-38-37.xad
Created: 11/7/2019 3:38:37 PM
Modified: 11/7/2019 4:02:30 PM

Gel Image

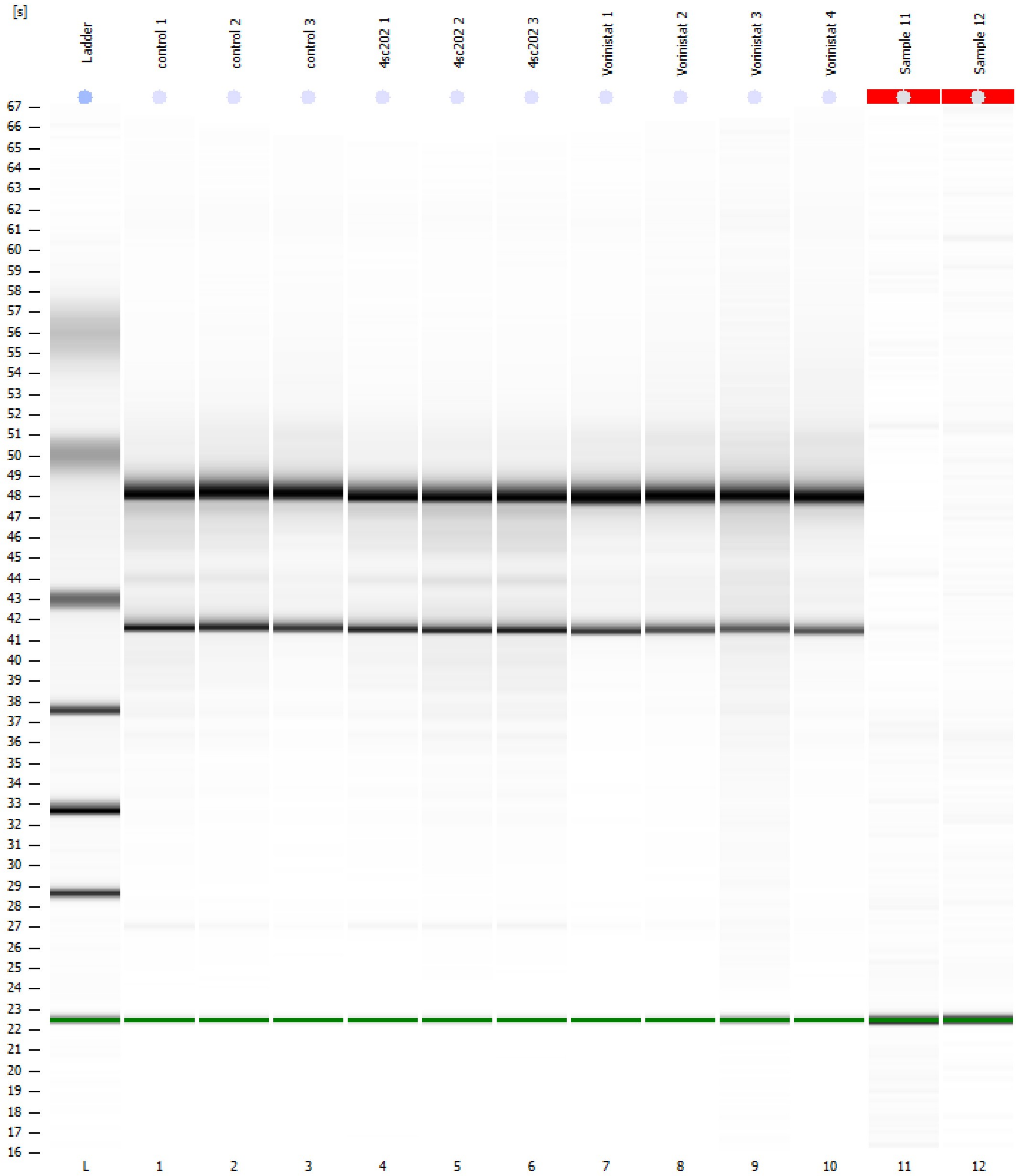

© Copyright 2003 - 2009 Agilent Technologies, Inc.
Printed: 11/7/2019 4:03:34 PM

Assay Class: Eukaryote Total RNA Nano
Data Path: C:\...Eukaryote Total RNA Nano_DE72902546_2019-11-07_15-38-37.xad
Created: 11/7/2019 3:38:37 PM
Modified: 11/7/2019 4:02:30 PM

Run Logbook

Description	Number	Source	Category	Sub Category	Time	Time Zone	User	Host
Run ended on port 5 (Number of wells acquired: 13)		Instrument	Run		11/7/2019 4:02:28 PM	(GMT --06:00) Central Standard Time	mukherjm	SANFORDHEALTH
Run started on port 5 (File: C:\Program Files (x86)\Agilent\2100 bioanalyzer\2100 expert\Data\2019-11-07\2100 expert_Eukaryote Total RNA Nano_DE72902546_2019-11-07_15-38-37.xad)		Instrument	Run		11/7/2019 3:38:43 PM	(GMT --06:00) Central Standard Time	mukherjm	SANFORDHEALTH
Product Number : G2939A		Instrument	Run		11/7/2019 3:38:43 PM	(GMT --06:00) Central Standard Time	mukherjm	SANFORDHEALTH
Name :		Instrument	Run		11/7/2019 3:38:43 PM	(GMT --06:00) Central Standard Time	mukherjm	SANFORDHEALTH
Vendor : Agilent Technologies		Instrument	Run		11/7/2019 3:38:43 PM	(GMT --06:00) Central Standard Time	mukherjm	SANFORDHEALTH
Serial# : DE72902546		Instrument	Run		11/7/2019 3:38:43 PM	(GMT --06:00) Central Standard Time	mukherjm	SANFORDHEALTH
Firmware : C.01.069		Instrument	Run		11/7/2019 3:38:43 PM	(GMT --06:00) Central Standard Time	mukherjm	SANFORDHEALTH
Cartridge : Electrode		Instrument	Run		11/7/2019 3:38:43 PM	(GMT --06:00) Central Standard Time	mukherjm	SANFORDHEALTH

 © Copyright 2003 - 2009 Agilent Technologies, Inc.
